# pipeComp, a general framework for the evaluation of computational pipelines, reveals performant single cell RNA-seq preprocessing tools

**DOI:** 10.1186/s13059-020-02136-7

**Published:** 2020-09-01

**Authors:** Pierre-Luc Germain, Anthony Sonrel, Mark D. Robinson

**Affiliations:** 1 Department of Molecular Life Sciences, University of Zürich, Winterthurerstrasse 190, Zürich, 8057 Switzerland; 2 SIB Swiss Institute of Bioinformatics, Zürich, Switzerland; 3 D-HEST Institute for Neurosciences, ETH Zürich, Winterthurerstrasse 190, Zürich, 8057 Switzerland

**Keywords:** Single-cell RNA sequencing (scRNAseq), Pipeline, Clustering, Normalization, Filtering, Benchmark

## Abstract

We present *pipeComp* (https://github.com/plger/pipeComp), a flexible R framework for pipeline comparison handling interactions between analysis steps and relying on multi-level evaluation metrics. We apply it to the benchmark of single-cell RNA-sequencing analysis pipelines using simulated and real datasets with known cell identities, covering common methods of filtering, doublet detection, normalization, feature selection, denoising, dimensionality reduction, and clustering. *pipeComp* can easily integrate any other step, tool, or evaluation metric, allowing extensible benchmarks and easy applications to other fields, as we demonstrate through a study of the impact of removal of unwanted variation on differential expression analysis.

## Background

Single-cell RNA-sequencing (scRNAseq) and the set of attached analysis methods are evolving fast, with more than 560 software tools available to the community [1], roughly half of which are dedicated to tasks related to data processing such as clustering, ordering, dimension reduction, or normalization. This increase in the number of available tools follows the development of new sequencing technologies and the growing number of reported cells, genes, and cell populations [2]. As data processing is a critical step in any scRNAseq analysis, affecting downstream analysis and interpretation, it is critical to evaluate the available tools.

A number of good comparison and benchmark studies have already been performed on various steps related to scRNAseq processing and analysis and can guide the choice of methodology [3–21]. However, these recommendations need constant updating and often leave open many details of an analysis. Another missing aspect of current benchmarking studies is their limitation to capture all aspects of a scRNAseq processing workflow. Although previous benchmarks already brought valuable recommendations for data processing, some only focused on one aspect of data processing (e.g., [14]), did not evaluate how the tool selection affects downstream analysis (e.g., [17]) or did not tackle all aspects of data processing, such as doublet identification or cell filtering (e.g., [18]). A thorough evaluation of the tools covering all major processing steps is however urgently needed as previous benchmarking studies highlighted that a combination of tools can have a drastic impact on downstream analysis, such as differential expression analysis and cell-type deconvolution[3, 18]. It is then critical to evaluate not only the single effect of a preprocessing method but also its positive or negative interaction with all parts of a workflow.

Here, we develop a flexible R framework for pipeline comparison and apply it to the evaluation of the various steps of analysis leading from an initial count matrix to a cluster assignment, which are critical in a wide range of applications. We collected real datasets of known cell composition (Table 1) and used a variety of evaluation metrics to investigate in a multilevel fashion the impact of various parameters and variations around a core scRNAseq pipeline. Although we use some datasets based on other protocols, our focus is especially on droplet-based datasets that do not include exogenous control RNA (i.e., spike-ins); see Table 1 and Additional File 1: Figure S1 for more details. In addition to previously used benchmark datasets with true cell labels [6, 15], we simulated two datasets with a hierarchical subpopulation structure based on real 10x human and mouse data using *muscat* [22]. Since graph-based clustering [23] was previously shown to consistently perform well across several datasets [6, 15], we used the *Seurat* pipeline as the starting point to perform an integrated investigation of (1) doublet identification, (2) cell filtering, (3) normalization, (4) feature selection, (5) dimension reduction, and (6) clustering. We compared competing approaches and also explored more fine-grained parameters and variations on common methods. Importantly, the success of methods at a certain analytical step might be dependent on choices at other steps. Therefore, instead of evaluating each step in isolation, we developed a general framework for evaluating nested variations on a pipeline and suggest a multilevel panel of metrics. Finally, we evaluate several recent methods and provide practical recommendations.

**Table 1:** Overview of the benchmark datasets

Dataset	# features	# cells	Protocol	Description
Koh	33922	531	SMARTer	9 FACS purified differentiation stages
Kumar	41930	246	SMARTer	Mouse ESC cultured in 3 different conditions
Zhengmix4eq	10434	3994	10x	Mixtures of FACS purified PBMCs
Zhengmix4uneq	11369	6498	10x	Mixtures of FACS purified PBMCs
Zhengmix8eq	10600	3994	10x	Mixtures of FACS purified PBMCs
mixology10x3cl	16208	902	10x	Mixture of 3 cancer cell lines from CellBench
mixology10x5cl	11786	3918	10x	Mixture of 5 cancer cell lines from CellBench
simMix1	3696	2500	10x-based	Simulation of 10 human cell subpopulations
simMix2	8893	3000	10x-based	Simulation of 9 mouse cell subpopulations

# features indicates the number of features detected in at least 10 cells

## Results

### A flexible framework for pipeline evaluation

**Fig. 1:**
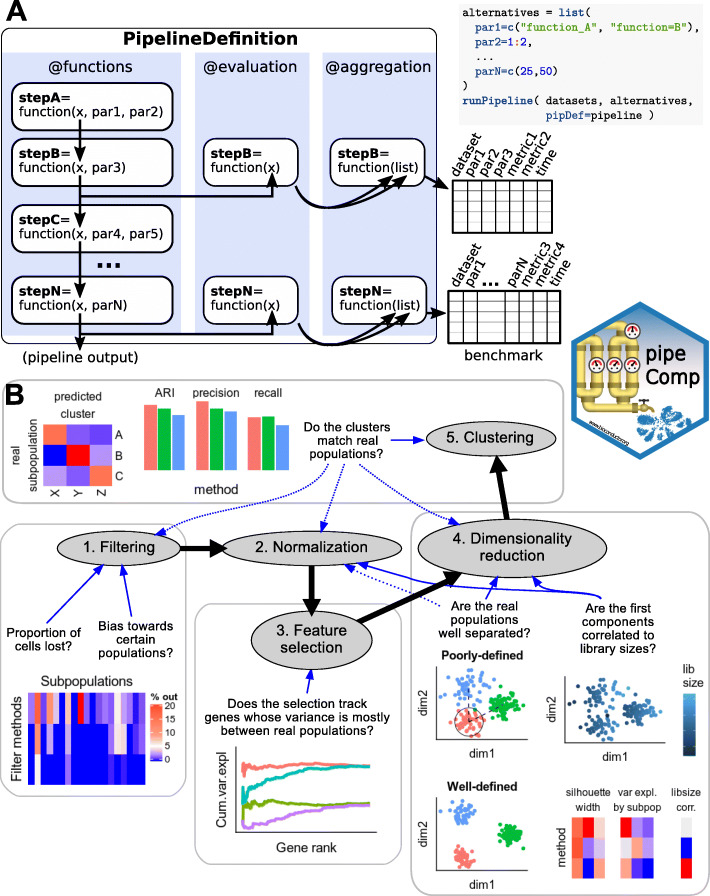
Overview of the *pipeComp* framework and its application to a scRNAseq clustering pipeline. **a** The package is built around a PipelineDefinition class which defines a set of functions to be executed consecutively, as well as optional evaluation functions for each step. Each step can accept a number of parameters whose alternative values are provided as a list. All (or subsets of) combinations of parameters can then be simultaneously ran and evaluated using the runPipeline function (see Additional File 2 for more details). **b** Scheme representing the application of *pipeComp* to evaluate a range of methods that are commonly used in scRNAseq studies and some of the metrics monitored at various steps

The *pipeComp* package defines a pipeline as, minimally, a list of functions executed consecutively on the output of the previous one (Fig. 1a; see also Additional File 2). In addition, optional benchmark functions can be set for each step to provide standardized, multi-layered evaluation metrics. Given such a PipelineDefinition object, a set of alternative parameters (which might include different subroutines calling different methods) and benchmark datasets, the runPipeline function then proceeds through all combinations of arguments (or a desired subset), avoiding recomputing the same step twice without the need to save all alternative intermediates, and compiling evaluations (including run time) on the fly (see Additional File 2 for more details). Variations in a given parameter can be evaluated using all metrics from this point downward in the pipeline. This is especially important because end-point metrics, such as the adjusted Rand index (ARI) [24] for clustering, are not perfect. For example, although the meaning of an ARI score is independent of the number of true subpopulations [25], the number of clusters called is by far the most important determinant of the score: the farther it is from the actual number of subpopulations, the worse the ARI (Additional File 1: Figure S2–3). In this context, one strategy has been to cluster across various resolutions and only consider the results that have the right number of clusters [6]. While this has the virtue of making the results comparable, in practice, the number of subpopulations is typically unknown and tools that operate well in this optimal context might not necessarily be best overall. Clustering results are also very sensitive and might not always capture improvements in earlier steps of the pipeline. In addition, we wanted to assess whether the effect of a given parameter alteration is robust to changes in the rest of the pipeline. We therefore monitored several complementary metrics across multiple steps of the process. We proceeded in a step-wise fashion, first testing a large variety of parameters at the early steps of the pipeline along with only a set of mainstream options downstream, then selecting main alternatives and proceeding to a more detailed benchmark of the next step (Fig. 1b).

### Filtering

#### Doublet detection

Doublets, defined as two cells sequenced under the same cellular barcode (e.g., being captured in the same droplet), are fairly frequent in scRNAseq datasets, with estimates ranging from 1 to 10% depending on the platform and cell concentration used [26, 27]. While doublets of the same cell type are relatively innocuous for most downstream applications due to their conservation of the relative expression between genes, doublets formed from different cell types or states are likely to be misclassified and could potentially distort downstream analysis. In some cases, doublets can be identified through their unusually high number of reads and detected features, but this is not always the case (Additional File 1: Figure S4). A number of methods were developed to identify doublets, in most cases by comparing each cell to artificially created doublets [28–30]. We first evaluated the capacity of these methods to detect doublets using the two 10x datasets with cells of different genetic identity [15], using SNP genotypes as the ground truth. For the sole purpose of this section, we included an additional dataset with SNP information but lacking true cell labels [27]. Of note, SNP-based analyses also call doublets created by cells of the same cell type (but from different individuals) and which are generally described as homotypic (as opposed to neotypic or heterotypic doublets, i.e., doublets from different cell types). These homotypic doublets might not be identifiable from the mere gene counts, and their identification is not generally the primary aim of doublet callers since they are often considered innocuous and, when across individuals, can be identified through other means (e.g., SNPs). We therefore do not expect a perfect accuracy in datasets involving cells of the same type across individuals (as in the demuxlet dataset).

We tested *DoubletFinder* [28] and *scran*’s doubletCells [29], both of which use similarity to artificial doublets, and *scds* [30], which relies on a combination of co-expression and binary classification. *DoubletFinder* integrates a thresholding based on the proportion of expected doublets, while *scran* and *scds* return scores that must be manually thresholded. In these cases, we ensured that the right number of cells would be called doublets. In addition to these methods, we reasoned that an approach such as *DoubletFinder* could be simplified by being applied directly on counts and by using a pre-clustering to create neotypic/heterotypic doublets more efficiently. We therefore also evaluated a simple and fast Bioconductor package implementing this method for doublet detection, *scDblFinder*, with the added advantage of accounting for uncertainty in the expected doublet rate and using meta-cells from the clusters to even include triplets (see the “Methods” section).

**Fig. 2:**
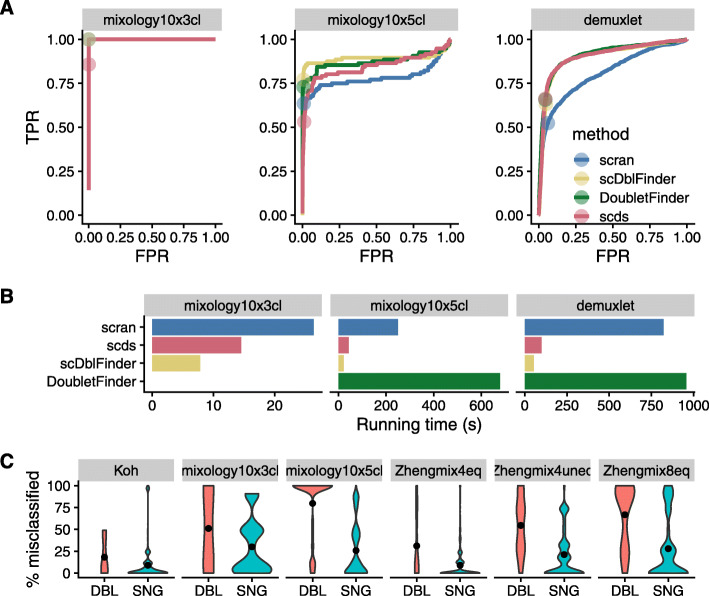
Identification of doublet cells. **a** Receiver operating characteristic (ROC) curves of the tested doublet detection methods for three datasets with SNP-identified doublets. Dots indicate threshold determined by the true number of doublets. **b** Running time of the different methods (*DoubletFinder* failed on one of the datasets). **c** Rate of misclassification of the cells identified by *scDblFinder* as doublets (DBL) or singlets (SNG), across a large range of clustering analysis. Even in datasets which should not have neotypic doublets, the cells identified as such tend to be misclassified

**Fig. 3:**
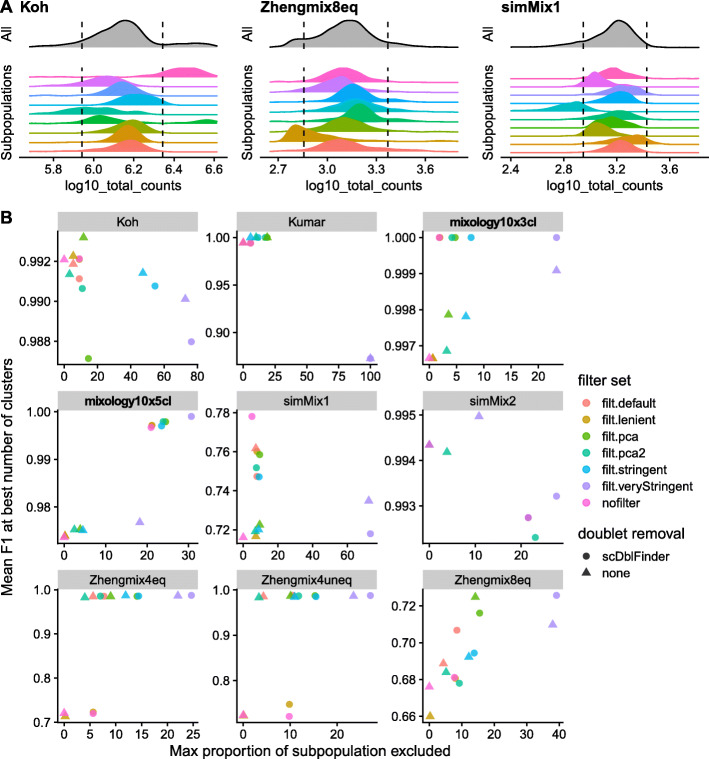
Effect of filtering on cell subpopulation structure and clustering. **a** Filtering on the basis of distance to the whole distribution can lead to strong bias against certain subpopulations. The dashed line indicates a threshold of 2.5 median absolute deviations (MADs) from the median of the overall population. **b** Relationship between the maximum subpopulation exclusion rate and the average clustering accuracy per subpopulation across various filtering strategies.The datasets for which the presence of doublets could be confirmed with SNP genotypes are labeled in bold. Of note, doublet removal appears to have a neutral or positive impact even when, due to the design, there are no heterotypic doublets in the data. The PCA methods refer to multivariate outlier detected as implemented in *scater* (see the “Methods” section for details)

While most methods accurately identified the doublets in the 3 cell lines dataset (mixology10x3cl), the other two datasets proved more difficult (Fig. 2a). *scDblFinder* achieved comparable or better accuracy than top alternatives while being the fastest method (Fig. 2b). Across datasets, cells called as doublets tended to be classified in the wrong cluster more often than other cells (Fig. 2c). We also found that *scDblFinder* clearly improved the accuracy of the clustering across datasets that are expected to have heterotypic doublets (mixology datasets) and even in a simulation (simMix1) that should not contain any (Fig. 3). Across the datasets that, being FACS-sorted, should not contain such doublets, doublet removal tended not to robustly impact clustering accuracy. However, in few datasets (ZhengMix4eq and Zhengmix4uneq, and simMix2 with a very small difference), doublet removal performed worse when combined with lenient or no filtering.

#### Excluding more cells is not necessarily better

Beyond doublets, a dataset might include low-quality cells whose elimination would reduce noise. This has for instance been demonstrated for droplets containing a high content of mitochondrial reads, often as a result of cell degradation and resulting loss of cytoplasmic mRNAs [31]. A common practice is to exclude cells that differ considerably from most other cells on the basis of such properties. This can for instance be performed through the isOutlier function from *scater* that measures, for a given control property, the number of median absolute deviations (MADs) of each cell from the median of all cells. Additional File 1: Figure S5 shows the distributions of some of the typical cell properties commonly used. Of note, these properties tend to be correlated, with some exceptions. For example, while a high proportion of mitochondrial reads is often correlated with a high proportion of the counts in the top features, there can also be other reasons for an over-representation of highly expressed features (Additional File 1: Figure S6), such as an over-amplification in non-UMI datasets. In our experience, 10X datasets also exhibit a very strong correlation between the total counts and the total features even across very different cell types (Additional File 1: Figure S7). We therefore also measure the ratio between the two and treat cells strongly departing from this trend with suspicion.

Reasoning that the cells we wish to exclude are the cells that would be misclassified, we measured the rate of misclassification of each cell in each dataset across a variety of clustering pipelines, correcting for the median misclassification rate of the subpopulation and then evaluated what properties could be predictive of misclassification (Additional File 1: Figure S8–10). We could not identify any property or simple combination thereof that would be consistently predictive of misclassification; the only feature that stood out across more than one dataset (the Zheng datasets) was that cells with very high read counts have a higher chance of being misclassified, although such a pattern was not observed in the other datasets.

We next investigated the impact of several filtering methods (see the “Methods” section for more details): four methods based on deviations to MADs with increasing levels of stringency (named *lenient*, *default*, *stringent*, *veryStringent*) and two methods based on *scater*’s *runPCA* using all (*pca.all*) or selected covariates (*pca.sel*). An important risk of excluding cells on the basis of their distance from the whole distribution of cells on some properties (e.g., library size) is that these properties tend to have different distributions across subpopulations. As a result, thresholds in terms of number of MADs from the whole distribution can lead to strong biases against certain subpopulations (Fig. 3a). We therefore examined the tradeoff between the increased accuracy of filtering and the maximum proportion of cells excluded per subpopulation (Fig. 3b and Additional File 1: Figure S11). Since filtering changes the relative abundance of the different subpopulations, global clustering accuracy metrics such as ARI are not appropriate here. We therefore calculated the per-subpopulation precision and recall using the Hungarian algorithm [32] and monitored the mean F1 score across subpopulations. A first observation was that, although more stringent filtering tended to be associated with an increase in accuracy, it tended to plateau and could also become deleterious. Most of the benefits could be achieved without very stringent filtering and minimizing subpopulation bias (Fig. 3b). Applying the same filtering criteria on individual clusters of cells (identified through scran’s quickCluster method) rather than on whole dataset resulted in nearly no cells being filtered out (Additional File 1: Figure S12A). This suggests that stringent filtering on the global population tends to discard cells of subpopulations with more extreme properties (e.g., high library size), rather than low-quality cells. In contrast, too lenient filtering (or lack thereof) can lead to misclassified cells, as was the case for the Zhengmix4 datasets (Fig. 3b). We found that a relatively mild filtering (*default*—see the “Methods” section) provided a good trade-off, often leading to high clustering performance (e.g., simMix and Zhengmix4 datasets) while retaining most of the cells in each subpopulation (e.g., Koh and Zhengmix8eq). PCA-based selection did not appear superior to feature-wise filters. We found very little overlap between the cells excluded by doublet removal and those excluded by MAD-based filtering (Additional File 1: Figure S12B). Of note, doublet removal in conjunction with filtering tended to improve accuracy not only in datasets that do include heterotypic doublets (mixology datasets), but also some others in which such doublets are unlikely given the experimental design (e.g., FACS-sorted datasets). Indeed, the datasets where doublet removal did not have a clear positive impact (e.g., Koh, Kumar, and the simulations) are those in which heterotypic doublets are not expected. Overall, we therefore recommend the use of doublet removal followed by our *default* filtering (see the “Methods” section) or a similar approach.

#### Filtering features by type

Mitochondrial reads have been associated with cell degradation and there is evidence that ribosomal genes can influence the clustering output, hiding other biological structure in the analysis [7]. We therefore investigated whether excluding one category of features or the other, or using only protein-coding genes, had an impact on the ability to distinguish subpopulations (Additional File 1: Figure S13). Removal of ribosomal genes robustly reduced the quality of the clustering, suggesting that they represent real biological differences between subpopulations. Removing mitochondrial genes and restricting to protein-coding genes had a very mild impact.

### Normalization and scaling

We next investigated the impact of different normalization strategies. Besides the standard log-normalization included in *Seurat*, we tested *scran*’s pooling-based normalization [33], *sctransform*’s variance-stabilizing transformation [34], normalization based on stable genes [35, 36], and *SCnorm* [37] (*scVI*-based normalization [38] was included separately, as it was not meant for this purpose and follows a slightly different flow). In addition to log-normalization, the standard *Seurat* clustering pipeline performs per-feature unit-variance scaling so that the PCA is not too strongly dominated by highly expressed features. We therefore included versions of the different normalization procedures, with or without a subsequent scaling (*sctransform*’s variance-stabilizing transformation involves an approach analogous to scaling). *Seurat*’s scaling function (and *sctransform*) also includes the option to regress out the effect of certain covariates. We tested its performance by using the proportion of mitochondrial counts and the number of detected features as covariates. Finally, it has been proposed that the use of stable genes, in particular cytosolic ribosomal genes, can be used to normalize scRNAseq [36]. We therefore evaluated a simple linear normalization based on the sum of these genes, as well as nuclear genes.

**Fig. 4:**
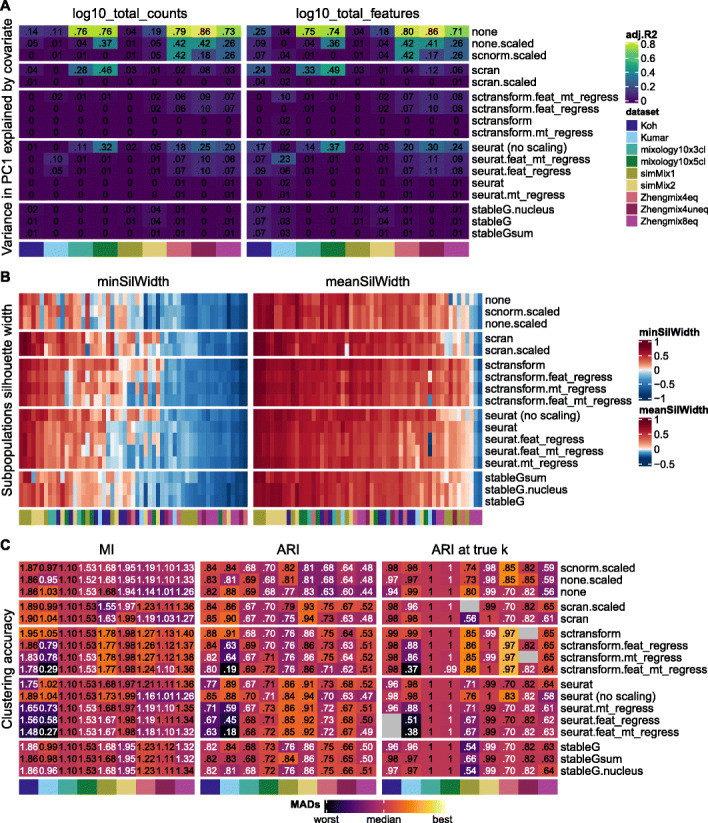
Evaluation of normalization procedures. **a** Variance in PC1 explained by library size (left) and detection rate (right) after accounting for subpopulation differences. **b** Average silhouette width per true subpopulation, where higher silhouette width means a higher separability. **c** Clustering accuracy (*Seurat* clustering), measured by mutual information (MI), Adjusted Rand Index (ARI), and ARI at the true number of cluster. Gray squares indicate that the true number of clusters could not be reached with the corresponding method, dataset, and parameter set. “feat_regress,” “mt_regress,” and “feat_mt_regress” respectively stand for the regressing out of the number of features, the proportion of mitochondrial reads, or both during scaling. Throughout this paper, silhouette plots (**b**) square root transform values for color mapping. Other evaluation metric heatmaps (e.g., **c** here) map colors to the signed square root of the number of (matrix-wise) median absolute deviations from the (column-wise) median. Using this mapping, *differences* in color have the same meaning across datasets, and the color scale is not primarily capturing outliers or baseline differences between datasets. The numbers printed in the cells represent the raw (i.e., unscaled) metric values and are printed in white or black depending on whether they are above or below the median

An important motivation for the development of *sctransform* was the observation that, even after normalization, the first principal components of various datasets tended to correlate with library size, suggesting an inadequate normalization [34]. However, as library size tends to vary across subpopulations, part of this effect can simply reflect biological differences. We therefore assessed to what extent the first principal component still retained a correlation with the library size and the number of detected features, removing the confounding covariation with the subpopulations (Fig. 4a and Additional File 1: Figure S14). Scaling tended to remove much of the correlation with these features, and while most methods were able to remove most of the effect, the correlation was lowest with *sctransform*. Surprisingly, however, regressing out the covariates tended to increase the association with it.

We further evaluated normalization methods by investigating their impact on the separability of the subpopulations. Since clustering accuracy metrics such as the ARI are very strongly influenced by the number of clusters, we complemented it with silhouette width [39] and mutual information (MI, Fig. 4b, c). We found most methods (including no normalization at all) to perform fairly well in most of the subpopulations and that some datasets (e.g., Zhengmix8eq and simMix1) and subpopulations were consistently more challenging across all methods. An exception was *scVI*-based normalization, which performed similarly to other methods on some datasets (Kumar, simMix2, Zhengmix4eq, Zhengmix4uneq), but very poorly on others, in terms of both the subpopulation silhouette and clustering accuracy (Additional File 1: Figure S13; of note, these values were not meant to be used for clustering, and the usage of the *scVI* latent space will be discussed below along with dimensionality reductions). Scaling (e.g., “seurat (no scale)” vs “seurat”) tended to reduce the average silhouette width of some subpopulations and to increase that of some less distinguishable ones and was generally, but not always, beneficial on the accuracy of the final clustering. Regressing out covariates systematically gave equal or poorer performance on MI, ARI, and ARI at true number of clusters, with a strongest negative impact among the Smart-seq datasets (Koh and Kumar). Regressing out covariates using *sctransform* instead tended to have a much milder effect, possibly owing to its regularization. In general, *sctransform* systematically outperformed other methods and, even though it was developed to be applied to data with unique molecular identifiers (UMI), it also performed fairly well with the Smart-seq protocol (Koh and Kumar datasets).

Finally, we monitored whether, under the same downstream clustering analysis, different normalization methods tended to lead to an over- or under-estimation of the number of clusters. Although some methods had a tendency to lead to a higher (e.g., *sctransform*) or lower (e.g., stable genes) number of clusters, the effect was very mild and not entirely systematic (Additional File 1: Figure S16). We also confirmed that, especially in the UMI-based datasets, *sctransform* did in fact successfully stabilize variance across mean counts (Additional File 1: Figure S17), at little apparent cost on computation time (Additional File 1: Figure S18).

### Feature selection

A standard clustering pipeline typically involves a step of highly variable genes selection, which is complicated by the digital nature and the mean-variance relationship of (sc)RNAseq. *Seurat*’s earlier approaches involved the use of dispersion estimates standardized for the mean expression levels, while more recent versions (≥ 3.0) rely on a different measure of variance, again standardized. While adjusting for the mean-variance relationship removes much of the bias towards highly expressed genes, it is plausible that this relationship may in fact sometimes reflects biological relevance and would be helpful in classifying cell types. Another common practice in feature selection is to use those with the highest mean expression. Recently, it was instead suggested to use deviance [40], while *sctransform* provides its own ordering of genes based on transformed variance.

**Fig. 5:**
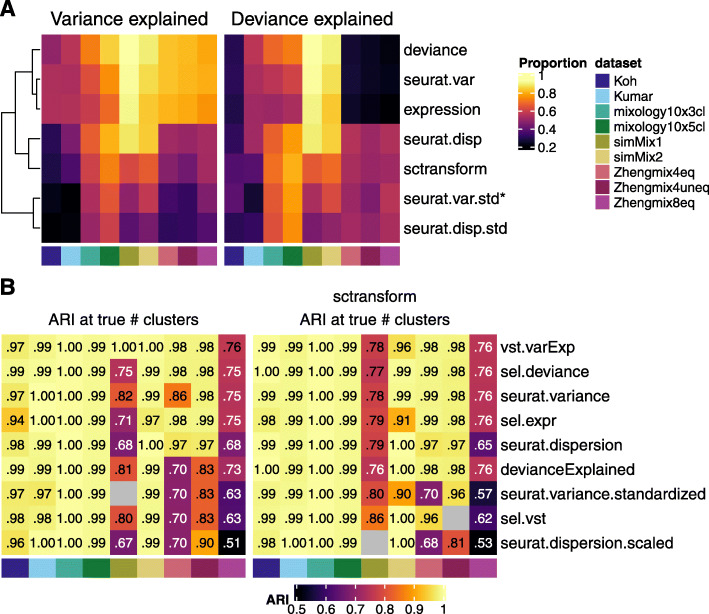
Evaluation of feature selection methods. **a** Ability of different feature ranking methods to capture genes with a high proportion of variance (left) or deviance (right) explained by real subpopulations. The asterisk denotes the default Seurat method. **b** Accuracy of clusterings (at the true number of clusters) when selecting 1000 genes using the given methods. Based on standard *Seurat* normalization (left) or *sctransform* (right). The *vst.varExp* and *devianceExplained* methods correspond to the estimates used in **a** to evaluate the selection methods and were included here only for validation purpose

Reasoning that a selection method should ideally select genes whose variability is higher between subpopulations than within, we first assessed to what extent each method selected genes with a high proportion of variance or deviance explained by the (real) subpopulations. As the proportion of variability in a gene attributable to subpopulations can be measured in various ways, we first compared three approaches: ANOVA on log-normalized count, ANOVA on *sctransform* normalization, and deviance explained. The ANOVAs performed on a standard *Seurat* normalization and on *sctransform* data were highly correlated (Pearson correlation > 0.9, Additional File 1: Figure S19A). These estimates were also in good agreement with the deviance explained, although lowly expressed genes could have a high deviance explained without having much of their variance explained by subpopulation (Additional File 1: Figure S19B–D). We therefore compared the proportion of the cumulative variance/deviance explained by the top X genes that could be retrieved by each gene ranking method (Additional File 1: Figure S20-21). We first focused on the first 1000 genes to highlight the differences between methods, although a higher number of selected genes decreased the differences between methods (Additional File 1: Figure S20-22). The standardized measures of variability were systematically worse than their non-standardized counterparts in selecting genes with a high proportion of variance explained by subpopulation. Regarding the percentage of deviance explained, however, the standardized measures were often superior (Fig. 5a and Additional File 1: Figure S20-21). *Deviance* proved the method of choice to prioritize genes with a high variance explained by subpopulations (with mere expression level proving surprisingly performant) but did not perform so well to select genes with a high deviance explained.

We next evaluated how the use of different feature selection methods affected the clustering accuracy (Fig. 5b). To validate the previous assay on the proportion of variance/deviance explained by real populations, we included genes that maximized these two latter measures. Interestingly, while these selections were on average the top-ranking methods, they were not systematically best for all datasets (Fig. 5b). Non-standardized measures of variability, including mere expression level, tended to outperform more complex metrics. In general, we found deviance and unstandardized estimates of variance to provide the best results across datasets and normalization methods. Increasing the number of features selected also tended to lead to an increase in the accuracy of the clustering, typically plateauing after 4000 features (Additional File 1: Figure S22).

### Dimensionality reduction

Since the various PCA approaches and implementations were recently benchmarked in a similar context [17], we focused on widely used approaches that had not yet been compared: *Seurat*’s PCA, *scran*’s denoisePCA, and GLM-PCA [40]. When relevant, we combined them with *sctransform* normalization. Given that *Seurat*’s default PCA weights the cell embeddings by the variance of each component, we also evaluated the impact of this weighting with each method.

The impact of the dimensionality reduction methods tested was far greater than that of normalization or feature selection (Additional File 1: Figure S23). Overall, weighting the components by their explained variance tended to increase silhouette width (Additional File 1: Figure S23A) and, in most datasets, clustering accuracy (Additional File 1: Figure S23B). GLM-PCA tended to increase the average silhouette width of already well-defined subpopulations, but *Seurat*’s PCA procedure however proved superior on all metrics. Like GLM-PCA, *scVI*’s Linearly Decoded (LD) data [41] and latent space do not explicitly rely on normalized counts. Their performance was however worse than *Seurat*’s PCA in view of the silhouette width (Additional File 1: Figure S15). In terms of clustering accuracy, *scVI*’s latent space performed better that its LD but was nevertheless less accurate than PCA-based alternatives on the datasets with a very low amount of cells (Koh and Kumar) and harboring many subpopulations (simMix1 and Zhengmix8eq).

#### Estimating the number of dimensions

A common step following dimension reduction is the selection of an appropriate number of dimensions to use for downstream analysis. Since Euclidean distance decreases as the number of non-discriminating dimensions increases, there is usually a trade-off between selecting enough dimensions to keep most information and excluding smaller dimensions that may represent technical noise or other unwanted sources of variation. Overall, increasing the number of dimensions robustly led to a decrease in the number of clusters (Additional File 1: Figure S16 and 24). This tended to affect the accuracy of the clustering (Additional File 1: Figure S25), although in both cases (number of clusters and ARI), *Seurat*’s resolution parameter had a much stronger impact.

Different approaches have been proposed to select the appropriate number of dimensions, from the visual identification of an inflexion point in the curve of the variance explained by each component (“elbow” method), to more complex algorithms. We evaluated the performance of dimensionality estimators implemented in the *intrinsicDimension* package [42], as well as common procedures such as the “elbow‘’ method, some scRNAseq-specific methods such as the JackStraw procedure [43] or *scran*’s denoisePCA [29], and the recent application of Fisher Separability analysis [44]. We also included a variation of the molecular cross-validation (MCVR, modified to be applied on normalized data—see the “Methods” section) approach originally developed to select an calibrate denoising methods [45], but which has also been applied to estimate dimensionality [46].

**Fig. 6:**
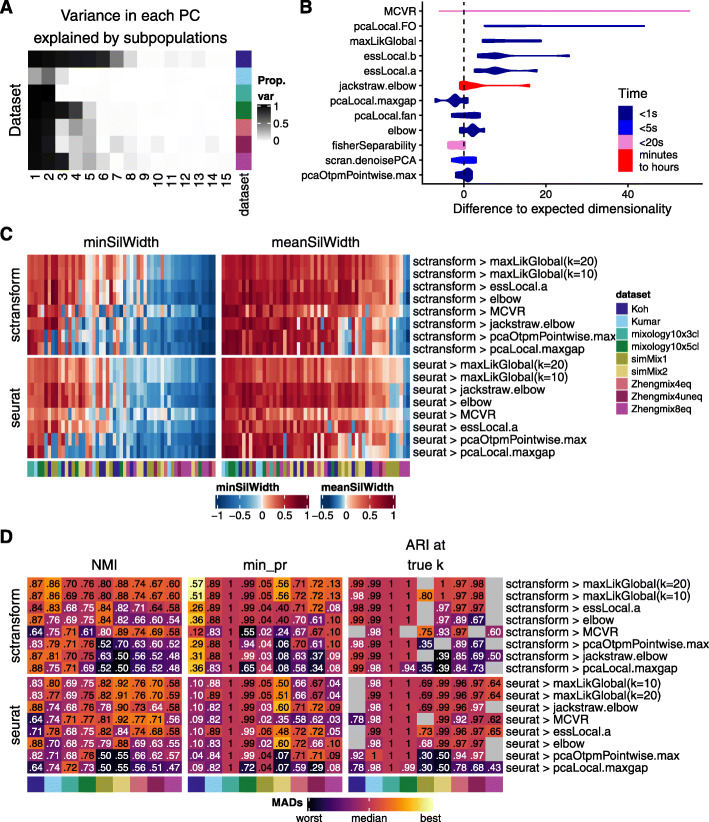
Estimating dimensionality. **a** Estimated dimensionality using the proportion of variance in each component explained by the true subpopulations. **b** Difference between “real” dimensionality (from **a**) and dimensionality estimation methods, along with computing time. **c** Average silhouette width per subpopulation across a selection of methods, combined with *sctransform* or standard *Seurat* normalization. **d** Clustering accuracy across the normalization/dimensionality estimation methods. The color-mapping scheme is the same as in Fig. 4c

We compared the various estimates in their ability to retrieve the intrinsic number of dimensions in a dataset, based on Seurat’s weighted PCA space. As a first approximation of the true dimensionality, we computed the variance in each principal component (based on Seurat’s weighted PCA space) that was explained by the subpopulations, which sharply decreased after the first few components in most datasets (Fig. 6a). These truth-based estimates were then compared to those of the above dimensionality estimation methods (Fig. 6b). Of note, the methods differ widely in compute time (Fig. 6b) and we saw no relationship between the accuracy of the estimates and the complexity of the method. Reasoning that over-estimating the number of dimensions is less problematic than under-estimating it, we kept the former methods for a full analysis of their impact on clustering (Fig. 6c-d), when combined with *sctransform* or standard *Seurat* normalization.

*MCVR* and the global maximum likelihood based on translated Poisson mixture model (*maxLikGlobal*) tended to produce clusterings with smaller deviations to the true number of subpopulations (Additional File 1: Figure S26). Although most methods performed well on the various clustering measures, *maxLikGlobal* (using 10 or 20 nearest neighbors, although estimates were relatively stable across various values of *k*—see Additional File 1: Figure S27) provided the dimensionality estimate that best separated challenging subpopulations (Fig. 6c) and tended to result in the best clustering accuracy (Fig. 6d). *MCVR* did not perform well for the datasets without UMIs (Koh and Kumar), which violates the assumptions of the method, and while it did increase the separability of some subpopulations (Fig. 6c), it was detrimental to the classification of some others. We observed a strong negative correlation (*r* = − 0.995, *p* 1.6e −8) between the number of dimensions called by *MCVR* and the log10 average library size of a dataset, which explained the former considerably better than the number of subpopulations.

Of note, the optimal method in our evaluation (*maxLikGlobal*) systematically selected many more components than were associated with the subpopulations in Fig. 6a, suggesting that although these additional components appear individually uninformative, in combination, they nevertheless contribute to classification. An important implication is that the commonly used elbow method most likely underestimates dimensionality.

### Clustering

The last step evaluated in our pipeline was clustering. Given previous work on the topic [6, 7] and the success of graph-based clustering methods for scRNAseq, we restricted our evaluation to Seurat’s method and variations of *scran*’s graph-based clustering using random walks (*walktrap* method) or the optimization of the modularity score (*fast_greedy*) on various underlying graphs. Specifically, we compared rank-based graphs (*scran*’s default) with graphs using the components’ values directly, SNN vs KNN graphs, and the exact nearest neighbors to the Annoy approximation. Again, the tested methods were combined with *Seurat*’s standard normalization and *sctransform*, otherwise using the parameters found optimal in the previous steps.

**Fig. 7:**
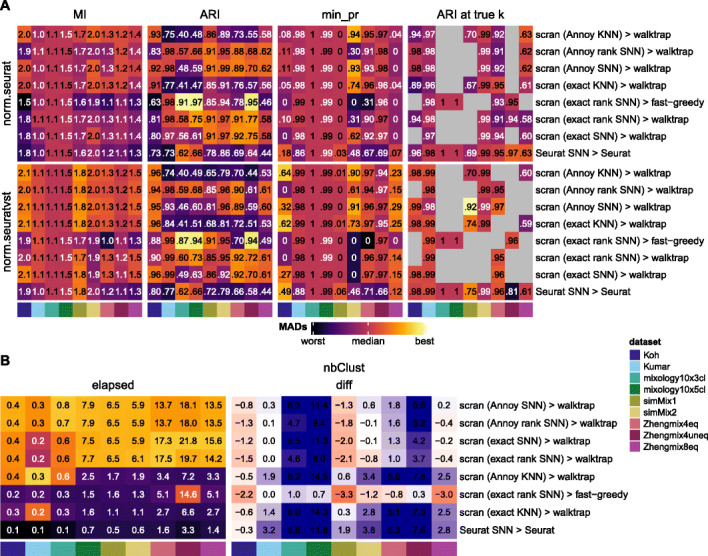
Evaluation of clustering methods. Clustering accuracy (**a**) and run time (**b**) of *scran*- and *Seurat*-based clustering tools in combination with *sctransform* and standard *Seurat*’s normalization. The color-mapping scheme in **a** and the left of **b** is the same as in Fig. 4c

Since ARI is dominated by differences in the number of clusters (Additional File 1: Figure S2–3) and no single metric is perfect, we diversified them (Fig. 7). MI has the virtue of not decreasing when a true subpopulation is split into two clusters, which is arguably less problematic (and might well reflect unknown biological subgroups), but as a consequence, it can be biased towards methods producing higher resolution clustering (Additional File 1: Figure S3). Similarly, precision per true subpopulation is considerably more robust to differences in the number of clusters, and we also tracked the ARI at the true number of clusters.

The MI score, which is largely independent of the estimated number of clusters, was overall higher for the *walktrap* method (Fig. 7a, left) over *Seurat* and the *fast-greedy* method. Depending on what network it was used with, however, it sometimes yielded a lower ARI or minimum precision. The ARI score at the true number of clusters, when available, showed similar performances, especially when using *sctransform* Because *Seurat*’s resolution parameter had a large impact on the number of clusters identified (Additional File 1: Figure S2 and 24), *Seurat* could always be coerced into producing the right number of clusters. Instead, the number of clusters found by *scran* was considerably less influenced by the available parameters (number of nearest neighbors or steps in the random walk—see Additional File 1: Figure S28), and as a result, *scran*-based clustering sometimes never produced a partitioning with the right number of clusters. This observation, along with *scran*’s higher MI score, suggest that *scran* sometimes simply divides a real subpopulation into two clusters (possibly tracking some unknown biological differences) rather than committing misclassification errors. Overall, the *walktrap* method appeared superior to the *fast greedy* algorithm and was generally comparable to *Seurat* clustering, although the latter offered more control over the resolution. A major difference between *walktrap*-based clustering and *Seurat* is the computing time (Fig. 7b). We found that using the Annoy approximation to the nearest neighbors somewhat reduced computing time at no apparent cost to accuracy. It nevertheless remained considerably slower than *Seurat*. For all methods, some poorly distinguishable subpopulations from both the Zhengmix8eq and simMix1 datasets remained very inaccurately classified in regard to all metrics.

### Overview of interactions between steps

**Fig. 8:**
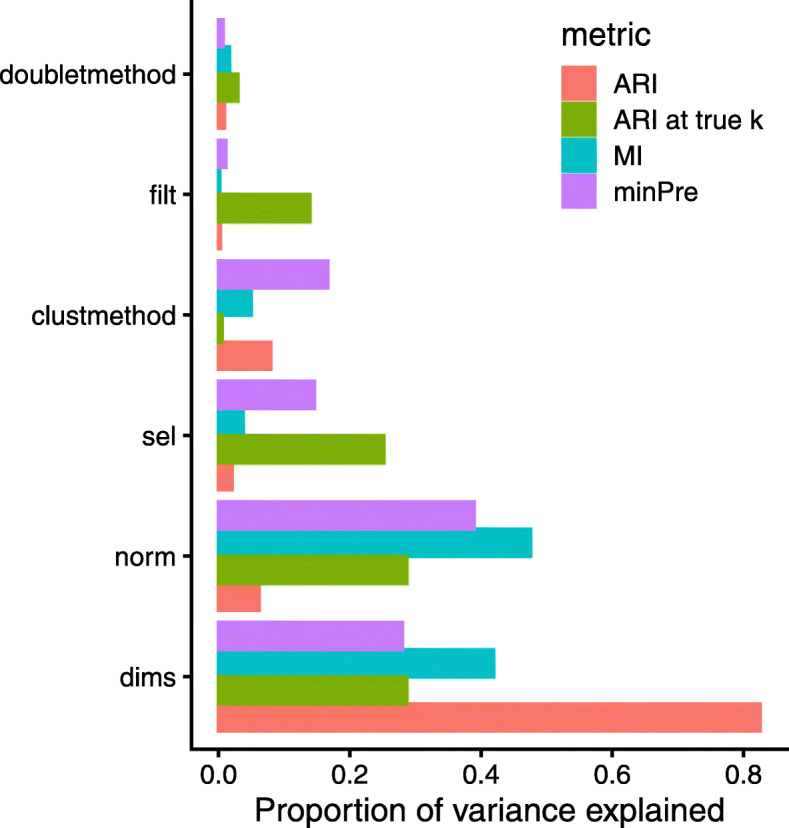
Variance in clustering metrics explained by the parameters. Proportion of variance explained by the parameters of the main analysis steps. The proportions are calculated from an ANOVA using the model metric ∼ dataset*resolution+doubletmethod+filt+norm+clustmethod+dims and substracting the variance attributable to differences due to datasets, resolutions, and their interactions

We selected the most important alternatives for each parameter (see the “Methods” section) in order to explore their interactions. Unsupervised clustering on the evaluation metrics reveals general patterns in the results as well as the relative importance of the parameters (Additional File 1: Figure S29), with the clustering method having the most important impact on the results. This is however chiefly due to number of clusters called: an ANOVA on all analyses after accounting, in the model, for baseline differences between datasets and the impact of *Seurat*’s resolution parameter, attributed relatively little importance to the clustering method, and instead attributed the largest proportion of variance in the main cluster metrics to the normalization method and the number of dimensions used (Fig. 8). Part of the impact of the estimated dimensionality depended on its effect on the number of clusters detected, and it indeed explained less variance in ARI at the true number of clusters (Fig. 8). Nevertheless, the choice of dimensionality (as well as feature selection) came closely behind normalization, suggesting that more attention should be paid to this relatively neglected step.

The greater importance of normalization, dimensionality, and feature selection over filtering steps is also visible in the clustering (Additional File 1: Figure S29). The top methods (bottom of Additional File 1: Figure S29) confirm the results of the previous sections, highlighting especially *sctransform*, deviance-based feature selection, and *maxLikGlobal* dimensionality estimates as top methods. Interestingly, while the combination of *sctransform* with *Seurat* clustering was particularly performant for most datasets, for the simulated “simMix1” dataset, standard *Seurat* normalization combined with *scran*-based clustering proved superior.

We next investigated whether the benefits of some methods were conditional on the choices at other steps. Although analysis of variance identified some significant interactions (Additional File 1: Figure S30), we found that the benefits brought by performant methods were robust to differences in other parts of the pipeline (e.g., Additional File 1: Figure S31). For instance, we confirmed that doublet removal and *sctransform* normalization were beneficial independently of other steps and that deviance-based feature selection outcompeted *Seurat*’s default selection method independently of changes at other steps (Additional File 1: Figure S31). ANOVA did reveal some significant (negative) interactions (Additional File 1: Figure S30): the top two-way interaction was between *scran* normalization and the elbow method for selection the number of dimensions, and indeed that combination was involved in all of the 30 lowest-ranking pipelines. Furthermore, all significant interactions involved the elbow method, suggesting that some methods were less robust to underestimated dimensionality.

### Further extensions to the pipeline: imputation/denoising

The basic pipeline presented here can be extended with additional analysis steps while keeping the same evaluation metrics. To demonstrate this, we evaluated various imputation or denoising techniques based on their impact on classification. Since preliminary analysis showed that all methods performed equally well or better on normalized data, we applied them after filtering and normalization, but before scaling and reduction. Although some of the methods (e.g., *DRImpute_process* and *alra_norm*) did improve the separability of some more elusive subpopulations, no method had a systematically positive impact on the average silhouette width across all subpopulations (Additional File 1: Figure S32A). When restricting ourselves to clustering analyses that yielded the “right” number of clusters, all tested methods improved classification compared to a scenario with no imputation step (“none” label, Additional File 1: Figure S32B). However, the situation was not so straightforward with alternative metrics, where some methods (e.g., *ENHANCE*) consistently underperformed. 10X datasets, which are typically characterized by a lower per-cell coverage and feature detection rate, benefited more from imputation and, in this context, *DrImpute* and *DCA* tended to show the best performance. On the contrary, imputing on normalized counts originating from Smart-seq technology was instead rather deleterious to the clustering accuracy.

### Application of *pipeComp* to a different context

**Fig. 9:**
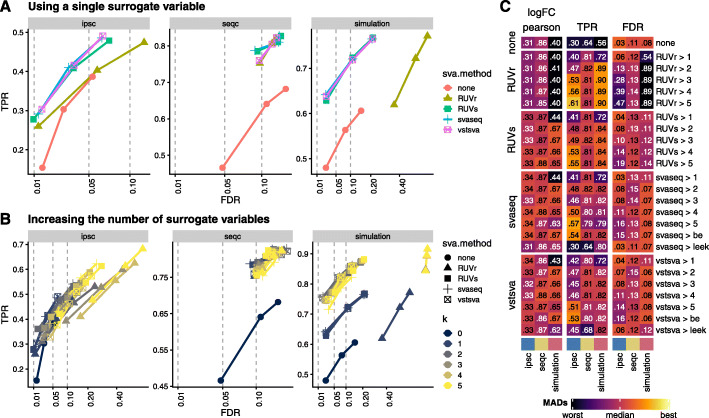
Evaluation of the impact of surrogate variable analysis (SVA) on (bulk) RNAseq differential expression analysis using *pipeComp*. *RUVr* and *RUVs* refer to the two *RUVSeq* functions by the same name; *svaseq* refers to the function of the same name in *sva*, while *vstsva* refers to the original *sva* method applied no variance-stabilized data. **a** True positive rates (TPR) and false discovery rates (FDR) of the SVA-based methods (using a single surrogate variable), averaged across DEA methods. **b** TPR and FDR of the different SVA-based methods (averaged across the different DEA methods) using increasing numbers of variables. In **a**, **b**, the *x*-axis is square root transformed to improve readability, and three dots for each combination of methods refer to the nominal FDR thresholds of 0.01, 0.05, and 0.1. **c** Correlation of estimated logFC with expected ones, as well as TPR and FDR (at nominal FDR threshold of 0.05) of the different methods with increasing numbers of variables. As in Fig. 4c, the color mapping tracks the number of (matrix-wise) median absolute deviations from the (column-wise) median, while the printed numbers represent the raw metric values. *be* and *leek* refer to the two methods implemented in the *sva* package for estimating the number of surrogate variables

To illustrate the applicability of *pipeComp* to a completely different problem, we benchmarked the impact of surrogate variable analysis (SVA)-based methods on bulk RNAseq differential expression analysis (DEA). The rationale behind such approaches is that the identification of factors determining genome-wide variation (i.e., surrogate variables) and their inclusion in the DEA’s generalized linear model might improve DEA, especially in the presence of sources of variation unrelated to the experimental variables. *sva* [47, 48] and *RUVseq* [49] are two popular packages each implementing different methods to infer these variables. We therefore created a PipelineDefinition with three steps: (1) feature filtering, (2) removal of unwanted variation (i.e., SVA), and (3) DEA using the most established methods [50]. The package’s “pipeComp_dea” vignette details the creation process. We developed 3 benchmark datasets: (1) the ipsc data is a random selection of 10 vs 10 samples from [51], which are very heterogeneous, and foldchanges were manually added to 300 genes; (2) the seqc dataset contains 5 vs 5 samples of two mixtures of the SEQC consortium with different ERCC spike-in mixes [52], and includes a partially correlated batch effect; (3) a simulation of 8 vs 8 samples with two technical vectors of variation (see the “Methods” section for more details). As previously reported [52], we found that more stringent filtering improved DEA (Additional File 1: Figure S33A–B) and that the different DEA methods had similar performances, especially after using SVA-based methods (Additional File 1: Figure S33B). Including a SVA-based step increased sensitivity across all datasets (Fig. 9a and Additional File 1: Figure S33B), particularly in datasets with technical vectors of variation (*seqc* and *simulation*), and benefited all DEA methods in a similar fashion (Fig. 9c and Additional File 1: Figure S33C–34A). With the exception of the RUVr method, which led to a high false discovery rate (FDR), all other methods showed comparable gains in sensitivity while maintaining a decent error control (if a little above the nominal FDR), even when increasing the number of surrogate variables used (Fig. 9b–c). While the FDR did increase in the *ipsc* dataset, we suspect that much of the effect could be due to spurious differences [53] between the groups which we wrongly assume to be false positives. Nevertheless, the FDR remained acceptable with up to three variables. These results therefore suggest that these methods are robust and can be widely applied.

## Discussion

### Practical recommendations

On the basis of our findings, we can make a number of practical analysis recommendations, also summarized in Additional File 1: Figure S35:

1. Filtering
  Doublet detection and removal is advised and can be performed at little computing cost with software such as *scDblFinder* or *scds*.
  Distribution-based cell filtering fails to capture doublets and should use relatively lenient cutoffs (e.g., 5 MADs, or 3 MADs in at least 2 distributions—see for instance our *default* filters in the the “Methods” section) to exclude poor-quality cells while avoiding bias against some subpopulations.
  Features filtering based on feature type did not appear beneficial.
2. Normalization and scaling
  Most normalization methods tested yielded a fair performance, especially when combined with scaling, which tended to have a positive impact on clustering.
  *sctransform* offered the best overall performance in terms of the separability of the subpopulations, as well as removing the effect of library size and detection rate.
  The common practice of regressing out cell covariates, such as the detection rate or proportion of mitochondrial reads nearly always had a negative impact, leading to increased correlation with covariates and decreased clustering accuracy. We therefore advise against this practice.
3. Feature selection
  Deviance [40] offered the best ranking of genes for feature selection.
  Increasing the number of features included tended to lead to better classifications, plateauing from 4000 features in our datasets.
4. Denoising/imputation
  Denoising appeared beneficial to the identification of some subpopulations in 10x datasets, but not in Smart-seq datasets.
  We found especially *ALRA* (with prior normalization), *DrImpute* (with prior processing), and *DCA* to offer the best performances, although none of these methods was beneficial over all subpopulations.
5. PCA
  Similarly to previous reports [14], we recommend the irlba-based PCA using weighting of the components, as implemented in *Seurat*.
  We advise against the commonly used elbow method (because it is too conservative) and the jackstraw method (low performance at great computational costs) for deciding on the components to include and recommend a method like the *global maximum likelihood based on translated Poisson mixture model* method (e.g., implemented in the maxLikGlobalDimEst function of intrinsicDimension, in our case using the 10 or 20 nearest neighbors).
6. Clustering
  In cases where prior knowledge can guide the choice of a resolution, *Seurat* can be useful in affording manual control of it while, in the absence of such knowledge, *scran*-based *walktrap* clustering provided reasonable estimates, although at greater computational costs.

### Limitations and open questions

In this study, we evaluated tools commonly used for the processing of scRNAseq with a focus on droplet-based datasets, namely from the 10x technology. Although this platform has been used in almost half of the scRNAseq studies in 2019 [2], other popular technologies such as Drop-seq, InDrops, or Smart-seq2/3 were not represented in the present benchmarking. Differences between such protocols, even among droplet-based technologies, can have a very large impact on cell capture efficiency, cell numbers, and clustering [54–56]. Although most top-ranking methods in our comparison performed well on both Smart-seq and 10x datasets that we tested, future benchmarking efforts should strive to include less represented technologies. In addition, we did not compare any of the alignment and/or quantification methods used to obtain the count matrix, which was for instance discussed in [18]. Some steps, such as the implementation of the PCA, were also not explored in detail here as they have already been the object of recent and thorough study elsewhere [14, 17]. We also considered only methods relying on Euclidean distance, while correlation was recently reported to be superior [57] and would require further investigation.

Among all tested pipelines, performance varied greatly across datasets. The ZhengMix8eq and simMix1 datasets were the most challenging datasets across all preprocessing steps that we tested. The average silhouette width of half of their subpopulations was systematically lower than the other subpopulations/datasets and the ARI score at true number of clusters, if ever achieved, was always poor. It is likely that these two datasets are challenging due to their combined sparsity and complexity (see Additional File 1: Figure S1). The challenge of distinguishing subpopulations of T-cells in the Zhengmix8eq dataset was previously discussed [6]. In contrast, while the Koh dataset harbors as many populations, they originate from more distinct groups (from hESC in different differentiation stages), while the Kumar and mixology datasets harbor a very low subpopulation complexity and most methods applied on them performed well.

Our recommendations are in line with the results of several other benchmarking studies. For instance, *Seurat* and *scran* methods were already shown to yield good clustering performance [7] and *DrImpute* to improve the quality of downstream analyses [21]. A previous systematic comparison [18] instead found that the use of *SAVERX* for denoising data and *scran* for clustering yielded better performance on the ability to recover differentially expressed genes, whereas we focused on the ability to recover cell populations. Another disagreement with the present recommendations come from the study of Hou et al. [10] that recommends *SAVERX* and discourage the use of *DCA* for better clustering, whereas our study found opposite results for these tools. Several differences in the design of our study could explain this contrast, such as the use of different metrics (ARI at true number of clusters, mean precision/ recall), of different datasets (Koh, Kumar, simMix simulations) and the use of different filtering and normalization strategies. *ALRA* however yielded good results in both studies and may be the most conservative option. The study from Hou et al. also highlighted the usefulness of *ALRA* when followed by differential and trajectory analysis both in UMI and Fluidigm technologies.

The performance of *scVI* in our evaluation was lower than in the original publication presenting the method [38]. Our results are however in line with other studies comparing silhouette score [58] and clustering accuracy [10] of *scVI*’s latent space. These studies, like ours, used datasets with relatively few cells (i.e., fewer than genes), for which *scVI* was reported by its authors to be less adapted. We think that further work will be required to assess its performance on larger datasets.

Here, we chose to concentrate on what could be learned from datasets with known cell labels (as opposed to labels inferred from the data, as in [54]). In contrast to Tian et al. [15], who used RNA mixtures of known proportions, we chose to rely chiefly on real cells and their representative form of variability. Given the limited availability of such well-described datasets, however, several aspects of single-cell analysis could not be compared, such as batch effect correction or multi-dataset integration. For these aspects of scRNAseq processing that are critical in some experimental designs, we refer the reader to previous evaluations [16, 59]. In addition, a focus on the identification of the subpopulations might fail to reveal methods that are instead best performing for tasks other than clustering, such as differential expression analysis or trajectory inference. Several informative benchmarks have already been performed on some of these topics [5, 11, 13, 19, 22, 60]. Yet, such evaluations could benefit from considering methods not in isolation, but as parts of a connected workflow, as we have done here. We believe that the *pipeComp* framework is modular and flexible enough to integrate new steps in the pipeline, as shown by an example with imputation/denoising methods.

We developed *pipeComp* to address the need of a framework that can simultaneously evaluate the interaction of multiple tools. The work of Vieth and colleagues[18] already offered an important precedent in this respect, evaluating the interaction of various steps in the context of scRNAseq differential expression analysis, but did not offer a platform for doing so. Instead, the *CellBench* package was recently proposed to address a similar need [61], offering an elegant piping syntax to combine alternative methods at successive steps. A key additional feature of *pipeComp* is its ability to perform evaluation in parallel to the pipeline and offering on-the-fly evaluation at multiple steps. This avoids the need to store potentially large intermediate data for all possible permutations and thus allowing a combinatorial benchmark. This, along with the set-up simplicity (demonstrated here by our separate SVA-DEA benchmark), also distinguishes *pipeComp* from general workflow frameworks such as *drake* [62]; indeed, we estimate that the benchmarks discussed here would have taken approximately 16TB of storage had all non-redundant intermediates been saved (see the Additional File 2 for more detailed explanations of the framework and comparison to *CellBench* and *drake*). In addition, the fact that benchmark functions are stored in the PipelineDefinition makes the benchmark more smoothly portable, making it easy to modify, extend with further methods, or apply to other datasets.

With respect to the methods themselves, we believe there is still space for improvement in some of the steps. Concerning cell filtering, we noticed that the current approach based on whole-population characteristic (e.g., MAD cut-off) can be biased against certain subpopulations, suggesting that more refined methods expecting multimodal distributions should be used rather than relying on whole-population characteristics. In addition, most common filtering approaches do not harness the relationship between cell QC properties. Finally, imputation had a varied impact on the clustering analysis and seemed to be linked to the technology that was used to generate the data. Also, the good performance of *DrImpute* is in line with a previous study focused on the preservation of data structure in trajectory inference from scRNAseq [21] (*ALRA* was however not included in this previous benchmark). Our results also align with the hypothesis of this study on the respective strengths of linear and non-linear models and their use to different types of data, such as the highest performance of non-linear methods in developmental studies.

## Conclusions

*pipeComp* is a flexible R framework for evaluating methods and parameter combinations in a complex pipeline, computing on-the-fly multilevel evaluation metrics. Applying this framework to scRNAseq clustering enabled us to make clear recommendations on the steps of filtering, normalization, feature selection, denoising, dimensionality reduction, and clustering (Additional File 1: Figure S35). We demonstrate how a diversity of multilevel metrics can be more robust, more sensitive, and more nuanced than simply evaluating the final clustering performance. We further showed that the *pipeComp* framework can be applied to extend the current field of benchmarking, as well as to investigate pipelines other domains with multi-step computational analyses.

## Methods

### Simulated datasets

The simMix1 dataset was based on the human PBMC CITE-seq data deposited under accession code GSE100866 of the Gene Expression Omnibus (GEO) website. Both RNA and ADT count data were downloaded from GEO and processed independently using *Seurat*. We then considered cells that were in the same cluster both in the RNA-based and ADT-based analyses to be real subpopulations and focused on the 4 most abundant ones. We then performed 3 sampling-based simulations with various degrees of separation using *muscat* [22] and merged the three simulations. The simMix2 dataset was generated from the mouse brain data published in [22] in a similar fashion. The specific code is available on https://github.com/markrobinsonuzh/scRNA_pipelines_paper.

### Default pipeline parameters

Where unspecified, the following default parameters or parameter sets were used:

- *scDblFinder* was used for doublet identification
- The default filter sets (see below) were applied
- Standard *Seurat* normalization was employed
- *Seurat* (≥3.0) variable feature selection was employed, selecting 2000 genes
- Standard *Seurat* scaling and PCA were employed
- To study the impact of upstream steps on clustering, various *Seurat* clustering analyses were performed, selecting different numbers of dimensions (5, 10, 15, 20, 30, and 50) and using various resolution parameters (0.005, 0.01, 0.02, 0.05, 0.1, 0.15, 0.2, 0.3, 0.4, 0.5, 0.8, 1, 1.2, 1.5, 2, 4). The range of resolution parameters was selected to ensure that the right number of clusters could be obtained in all datasets.

### Denoising/imputation

Most imputation/denoising methods were run with the default parameters with the following exceptions; *DrImpute* documentation advises to process the data prior to imputation by removing lowly expressed genes and cells expressing less than 2 genes. As it is not clear if this step is a hard requirement for the method to perform well, *DrImpute* was run with and without prior processing (*DrImpute_process* and *DrImpute_noprocess* labels, respectively). The *DCA* method only accepts integers counts, while two of the datasets had non-integer quantification of expected counts. For these datasets, we rounded up the counts prior to imputation. *ALRA* is designed for normalized data but as we are evaluating normalization downstream to imputation, we used the method on both non-normalized (*alra* label) and normalized counts (*alra_norm* label). *scImpute* requires an estimation of the expected number of clusters with the input data. As the estimation of the true number of cluster may not be known by the user, we evaluated the tool using the true number of clusters (*scImpute* label) and using an over/under-estimation of this number (*scImpute_plus5* and *scImpute_min5* labels, respectively). *ENHANCE* uses a k-nearest neighbor aggregation method and automatically estimates the number of neighbors to merge prior to the imputation. With the smallest datasets, this parameters was estimated to be 1, which lead to an early stop of the function. In such cases, we manually set this parameter to 2 for the method to work.

### Dimensionality estimates

For the methods that produce local dimensionality estimates, we used the maximum. For the elbow method, we implemented an automatic procedure by taking the farthest point from a line drawn between the variance explained by the first and last (i.e., 50th) components calculated. For the JackStraw method, since the *Seurat* documentation advises not to use a *p* value threshold (which would typically yield a very large number of dimensions) but rather look for a drop in significance, we applied the same farthest point algorithm on the log10(*p* values), which in our hands reproduced manual threshold selection.

For the molecular cross-validation approach, we relied on a R implementation by Matthew Young (https://github.com/constantAmateur/MCVR), with minor modifications (see wrapper in the *pipeComp* package). Briefly, since there is no a priori relationship between the dimensionality of the count data and that of the PCA based on the normalized data, MCVR does not use the original approach based Poisson loss, but the mean squared error of the normalized data, and uses balanced 50-50 splits. Because of the 50% split, dimensionality may be underestimated, and titration did suggest that was the case for some of our datasets.

### Filter sets

The *default* set of filters excludes cells that are outliers according to at least two of the following thresholds: log10_total_counts >2.5 MADs or <5 MADs, log10_total_features >2.5 MADs or <5 MADs, pct_counts_in_top_20_features > or <5 MADs, featcount_dist (distance to expected ratio of log10 counts and features) > or <5 MADs, pct_counts_Mt >2.5 MADs and >0.08.

The *stringent* set of filters uses the same thresholds, but excludes a cell if it is an outlier on any single distribution. The *veryStringent* set of filters excludes any cell that is an outlier by at least 2 MADs (lower or higher) on any of the following: log10_total_counts, log10_total_features, pct_counts_Mt, or pct_counts_in_top_20_features. The *lenient* set of filters excludes cells that are outliers on at least two distributions by at least 5 MADs, except for pct_counts_Mt where the threshold is >3 MADs and >0.08.

For cluster-wise filters, clusters were first identified with *scran::quickCluster* and the filters were then applied separately for each cluster.

The “pca.all” and “pca.sel” clusters refer to the multivariate outlier detection methods implemented in *scater*, running *runPCA* with use_coldata=TRUE, detect_outliers=TRUE. “pca.all” uses all covariates, while “pca.sel” uses only the log10(counts), log10(features), proportion mitochondrial, and proportion in the top 50 features.

### Variance and deviance explained

Unless specified otherwise, the variance in gene expression explained by subpopulations was calculated on the data normalized and transformed through *sctransform*. For each gene, we fitted a linear model using the subpopulation as only independent variable (∼*subpopulation*) and used the R-squared as a measure of the variance explained. The same method was used for principal components.

The deviance explained by subpopulations was calculated directly on counts using the getDevianceExplained function of *pipeComp*. The function uses *edgeR* to fit two models, a full (∼*librarySize*+*population*) and a reduced one (∼*librarySize*). For each gene, the deviance explained is then the difference between the deviance of each models, divided by the deviance of the reduced model. In the rare cases where this resulted in a negative deviance explained, it was set to zero.

To estimate the correlation between the principal components and covariates such as library size, we first fitted a linear model on the subpopulations and correlated the residuals of this model with the covariate of interest.

### *scVI* normalization and LD

The evaluation of *scVI* algorithm was performed separate to the other pipeline combinations since the model training on our reference datasets was especially resource-consuming and follows a different workflow from the other pipelines. In particular, for datasets with fewer cells than genes, *scVI* requires a feature selection to be performed prior to the normalization step; furthermore, its dimensional reduction steps rely directly on raw counts. We therefore evaluated it separately and compared it to a representative subset of pipelines using *sctransform* normalization and *Seurat*’s PCA dimension reduction method. The resulting evaluation is available in the Additional File 1: Figure S13.

Using *scVI*, we considered as normalized values the normalized mean of the negative binomial distribution modeling the counts (accessed through the *get_sample_scale* method). Following *scVI*’s online documentation, we used the *VAE* model, using a train size of 1, a training on 400 epochs, and a learning rate of 0.001. As a major limitation of *scVI* is the training on dataset with low gene to cell ratio, the features were subsampled to 2000 using the *subsample_genes* function, which resembles the *Seurat* V3 VST gene selection method. All other parameters were left unmodified.

Two models of the *scVI* package computing a dimension reduction on the data were tested. First, we evaluated the mean of the posterior distribution for the latent space, still based on the *VAE* model (accessed through the *get_latent* function). All other parameters were set as described above or left as default. The second model that we evaluated was the linearly decoded VAE of *scVI* (*LDVAE* model). It was trained on 250 epochs, a learning rate of 0.001 and computed 50 dimensions. For some smaller datasets, the training phase aborted. In such cases, we followed the recommendations of the package and reduced the learning rate by a factor 10 until the model could properly converge. Given that the other methods that we evaluated returned PCs ordered by their variance explained, we applied to the same reordering to the latent space returned by *scVI*. For both models. we relied on the feature selections results from the pipeline and not on the *subsample_genes* function. All other parameters were left unmodified.

### Overall run for interactions across steps

The set of alternatives found for the overall run (Fig. 8 and Additional File 1: Figure S29-31) can be found in the paper’srepository . The analysis of variance was performed with the following model:

metric ∼ dataset*resolution+doubletmethod+filt+norm+clustmethod+dims

### SVA-DEA benchmark

The full process of creating the SVA-DEA PipelineDefinition is described step by step in the pipeComp_deavignette .

The three benchmark datasets are available in https://github.com/markrobinsonuzh/scRNA_pipelines_paper/blob/master/svadea/datasets/, along with all code used to generate and prepare them. Briefly:

- For the *ipsc* dataset, we took the quantification of the GSE79636 dataset (fairly heterogeneous) from the *iPSCpower* package [53] and selected two random sets of 10 samples. No further variation was added, and log-foldchanges ranging from 0.25 to 1.25 were added to the counts of 300 random genes. All other genes were assumed to be negatives.
- For the *seqc* dataset, we used data from the *seqc* package [52]. For both groups (mixtures C and D, which also contain two different spike-in mixes), we selected samples from the ILM_refseq_gene_AGR and ILM_refseq_gene_CNL batches, which have clear technical differences; we selected 5 samples per group with a weak correlation with the batch (3:2 vs 2:3). Since the true differences between mixtures are not entirely known, the analysis was performed on all genes but only the spike-ins were considered for benchmarking.
- For the *simulation*, we simulated counts using the *polyester* package [63] and the means/dispersions from the GSE79636 dataset (restricted to a single batch and biological group), with 500 DEGs (log-foldchanges ranging from 0.5 to 2) and two batch effects: (1) a technical batch partially correlated with the group (6:2) and affecting 1/3 of the genes and (2) a linear vector of variation uncorrelated with the groups and affecting 1/3 of the genes.

Filtering was performed with edgeR’s filterByExpr function, changing the min.count argument. For *vstsva*, we first applied the variance-stabilizing transformation from *DESeq2* [64] before applying sva::sva. *edgeR.QLF* refers to the quasi-likelihood version [65] of *edgeR*’s fit. The exact wrappers used can be found in the package at https://github.com/plger/pipeComp/blob/master/inst/extdata/dea_wrappers.R.

### Software and package versions

Analyses were performed in R 3.6.0 (Bioconductor 3.9) and the following packages were installed from GitHub repositories: *Seurat* (version 3.0.0), *sctransform* (0.2.0), *DoubletFinder* (2.0.1), *scDblFinder* (1.1.4), *scds* (1.0.0), *SAVERX* (1.0.0), *scImpute* (0.0.9), *ALRA* (commit 7636de8), *DCA* (0.2.2), *DrImpute* (1.2), *ENHANCE* (R version, commit 1571696), and *SAVERX* (1.0.0). The glmPCA was performed using the *glmpca* package, while the code for deviance calculation was obtained from https://github.com/willtownes/scrna2019 (commit 1ddcc30ebb95d083a685f12fe81d35dd1b0cb1b2).

## Supplementary information

**Additional file 1** Supplementary Figures (PDF 16 MB)

**Additional file 2** More detailed description of *pipeComp* and comparison with other tools. (PDF 0.5 MB)

**Additional file 3** Review history.

## Data Availability

All analyses were performed through the pipeComp R package, which implements the pipeline framework described here. scVI results and results of the clustering section were produced with *pipeComp* 0.99.4, while all other results were produced with version 0.99.2 (*biorxiv*) (branch of the repository). All code to reproduce the simulations and figures is available in the https://github.com/markrobinsonuzh/scRNA_pipelines_paper repository, which also includes the basic datasets with a standardized annotation. All wrappers for methods used in the study can be found in the external data of the *pipeComp* package (https://github.com/plger/pipeComp/tree/master/inst/extdata). In addition, both the package and attending code are archived on [66]. The gene counts for the two mixology datasets were downloaded from the CellBenchrepository, commit 74fe79e. For the other datasets, we used the unfiltered counts from [6], available on the corresponding repository https://github.com/markrobinsonuzh/scRNAseq_clustering_comparison. For simplicity, all starting *SingleCellExperiment* objects with standardized metadata are available on [67]. All data and code are released under a GNU General Public License.
