# Supplementary material for: pipeComp, a general framework for the evaluation of computational pipelines, reveals performant single cell RNA-seq preprocessing tools
Source: Genome Biol. 2020 Sep 1;21:227. doi: 10.1186/s13059-020-02136-7 (PMC7465801; doi:10.1186/s13059-020-02136-7)
Supplement: Supplementary file 1 — Additional file 1 Supplementary Figures (PDF 16 MB) [file 13059_2020_2136_MOESM1_ESM.pdf]

pipeComp, a general framework for the evaluation of computational pipelines, reveals performant single-cell RNA-seq preprocessing tools

Additional File 1 - Supplementary Figures

Pierre-Luc Germain
Anthony Sonrel
Mark D. Robinson

04 August, 2020

Fig S1

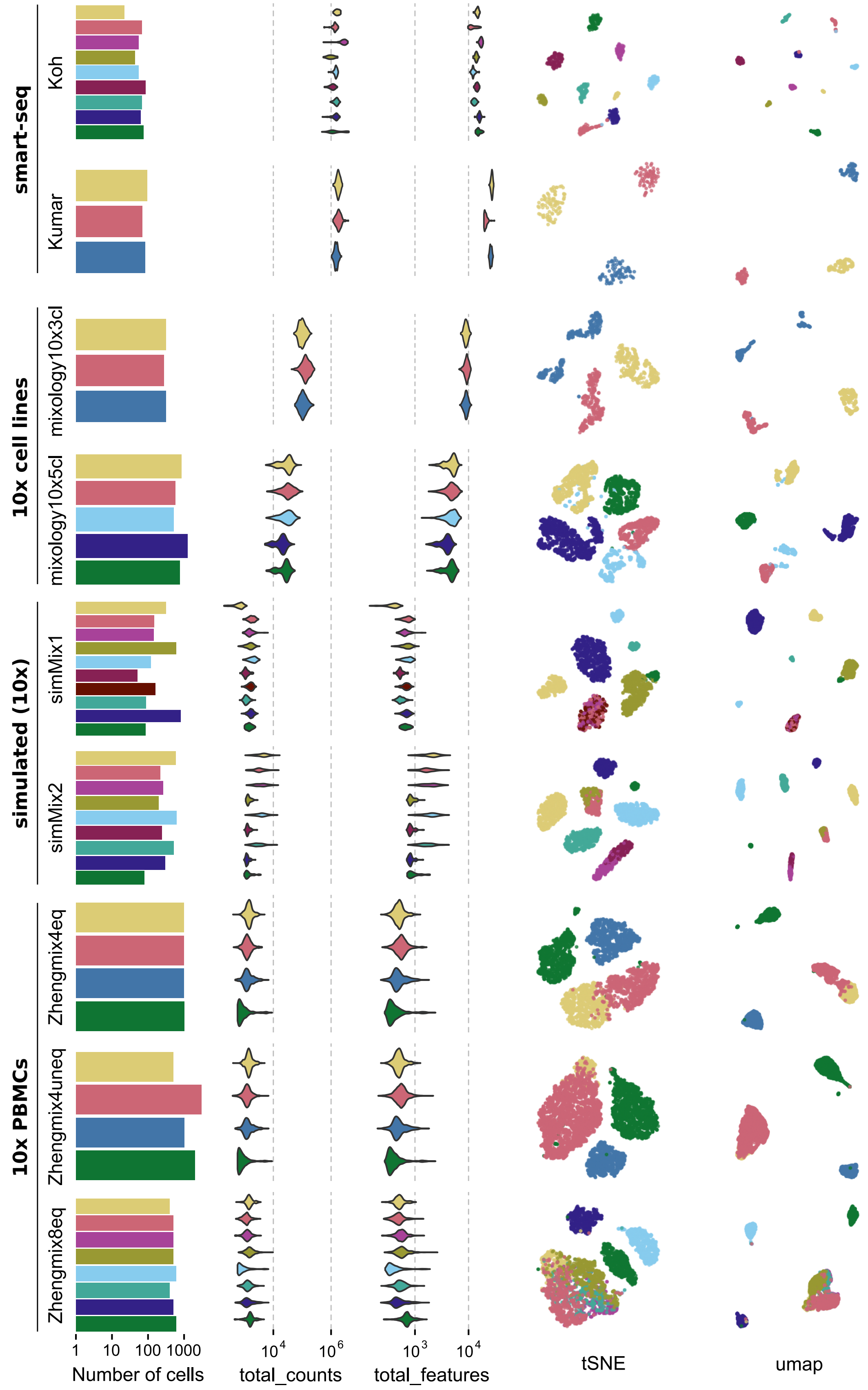

Fig S1: Overview of the benchmark datasets

Fig S2

```
## Warning: Removed 169 rows containing missing values (geom_point).
```

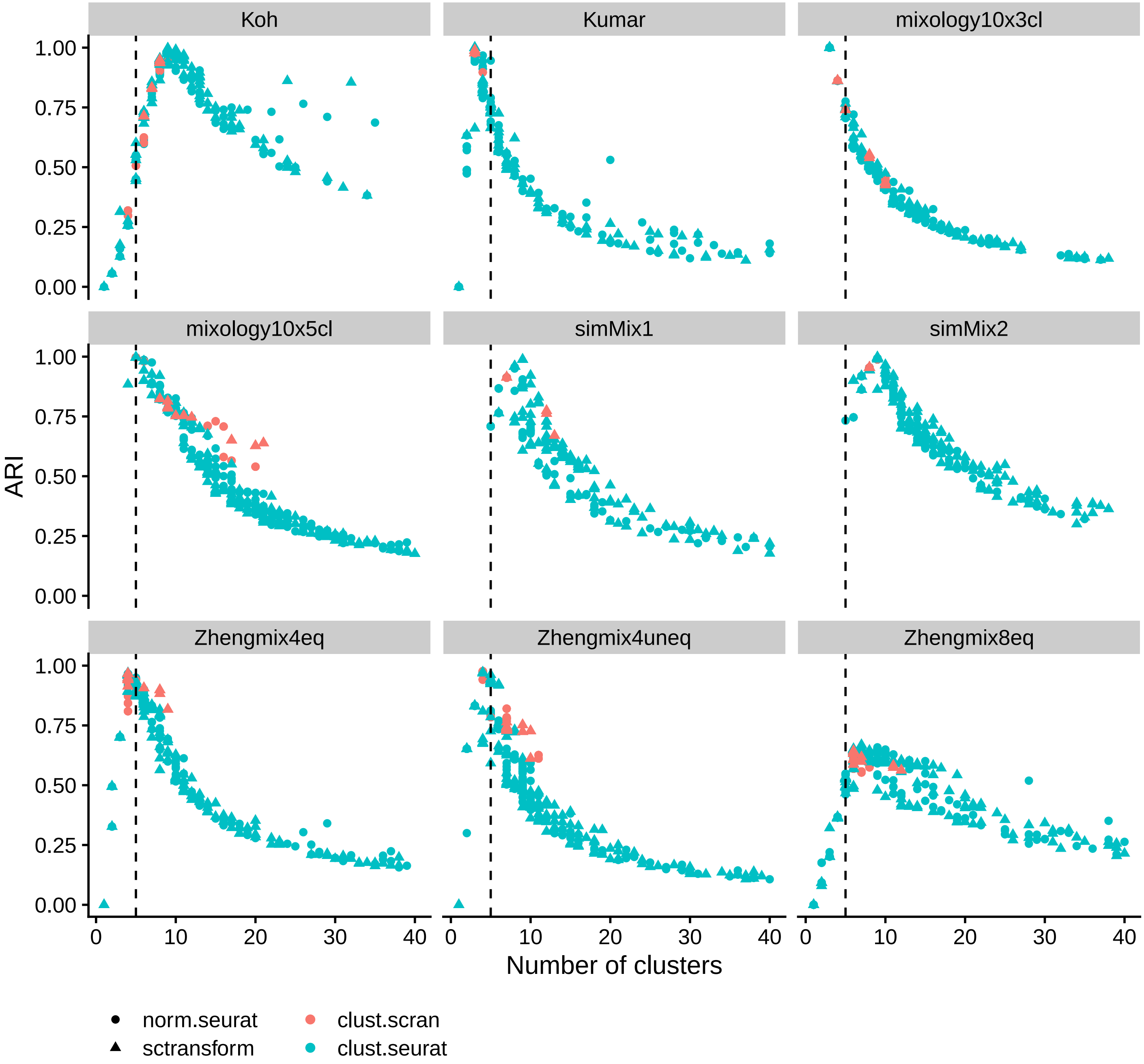

Fig S2

The number of clusters called has a much bigger impact on the Adjusted Rand Index (ARI) than differences between methods. The dashed line indicates the true number of clusters.

Fig S3

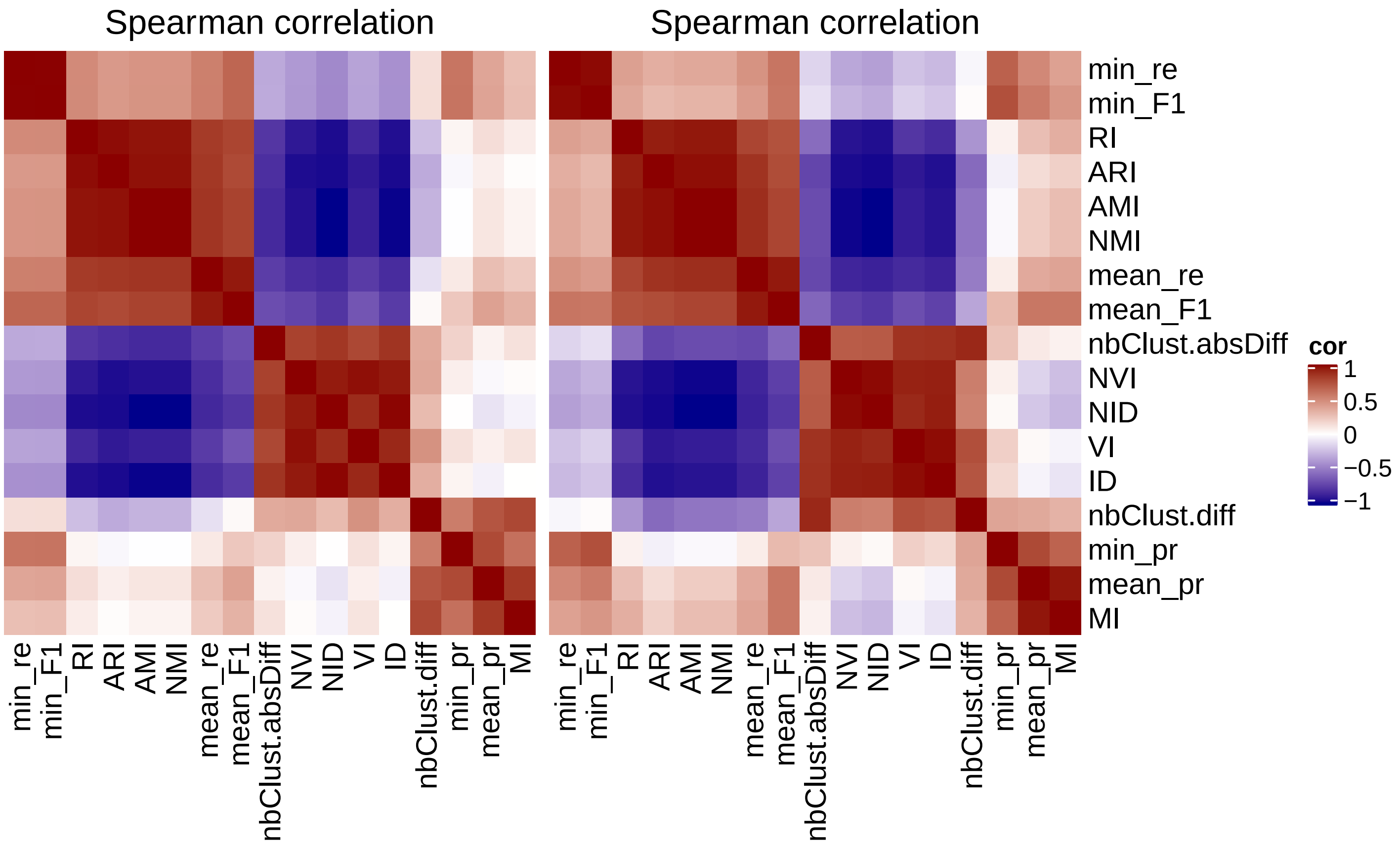

Fig S3

Relationship of various metrics of clustering accuracy between each other and with variations in the number of clusters called (`nbClust.diff` and `nbClust.absDiff`). Correlations were calculated for each dataset separately across various clustering runs and averaged (the `mixology10x3cl` dataset was excluded due to insufficient variation among the results). Information distance metrics (ID, NID, VI, NVI) are highly correlated with the absolute difference between the true and called number of clusters, while the Adjusted Rand Index (ARI) and similar metrics were strongly anticorrelated to it. Precision (mean_pr) and recall (mean_re) were slightly less correlated with discrepancies in the number of clusters. Mutual information (MI) was not at all correlated with the absolute difference in number of clusters (`nbClust.absDiff`), but positively correlated with the difference (`nbClust.diff`), i.e. favouring clusterings calling a higher number of clusters. We therefore recommend using complementary metrics such as ARI and MI, and potentially mean F1 per subpopulation.

Fig S4

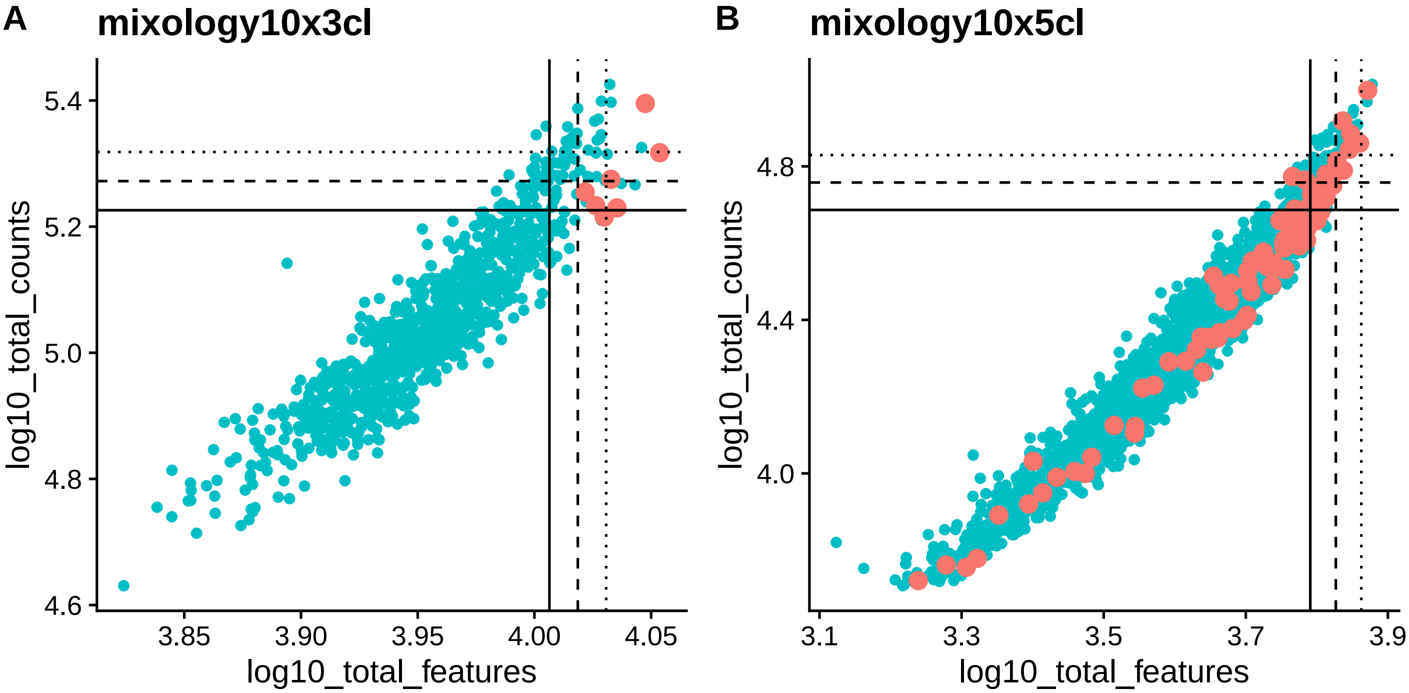

Fig S4

The total counts and total features per cell of doublets (red) versus other cells. We used the demuxlet annotation of doublets (based on SNPs) made available through CellBench. The lines indicate, respectively, 2, 2.5, and 3 median absolute deviations. While doublets tend to have a higher total count and especially number of detected features, these features alone are not always sufficient for their identification.

Fig S5

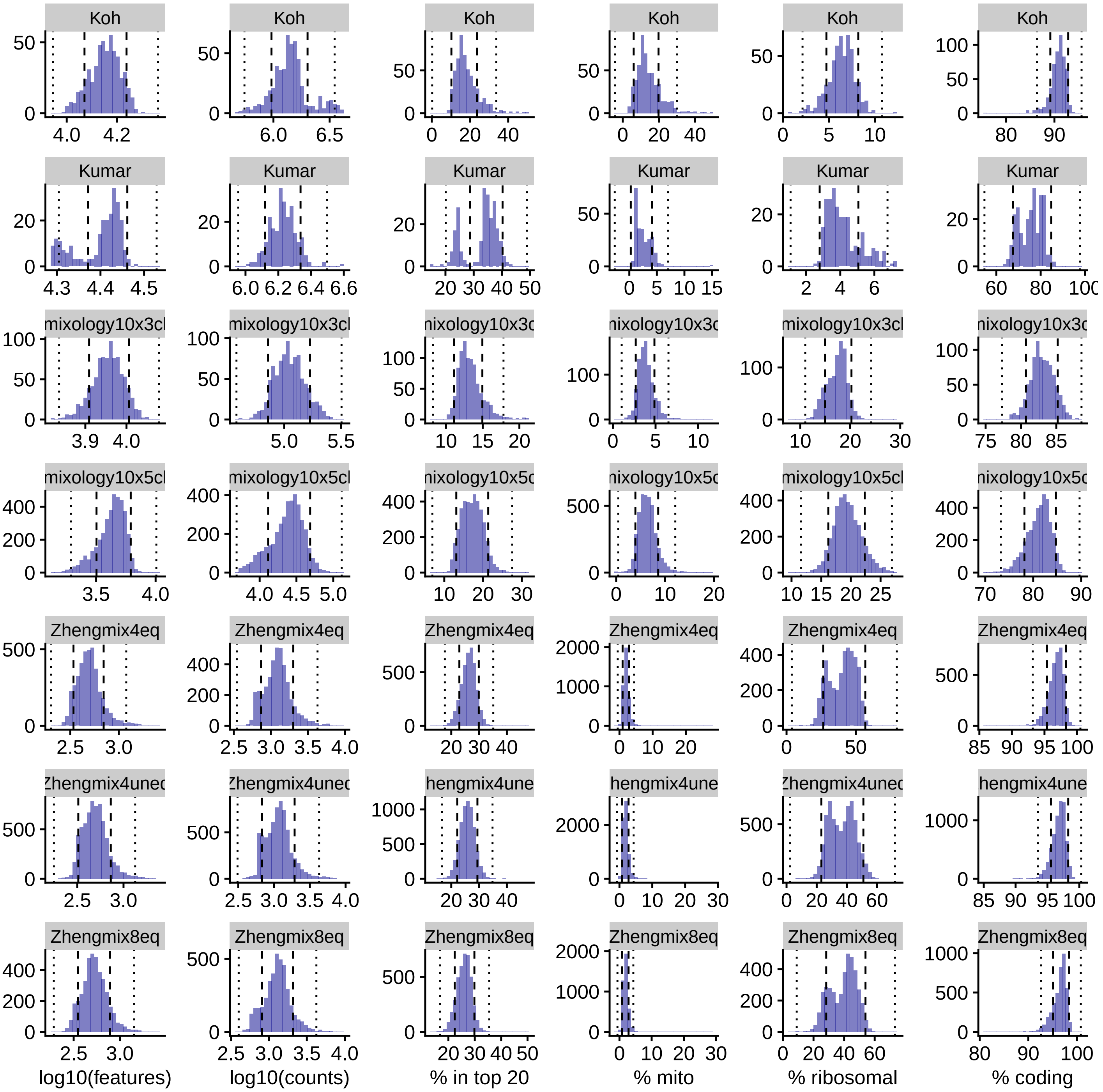

Fig S5

Distribution across cells of various control properties in the different datasets. The lines indicate respectively 2 and 5 median absolute deviations (MADs).

Fig S6

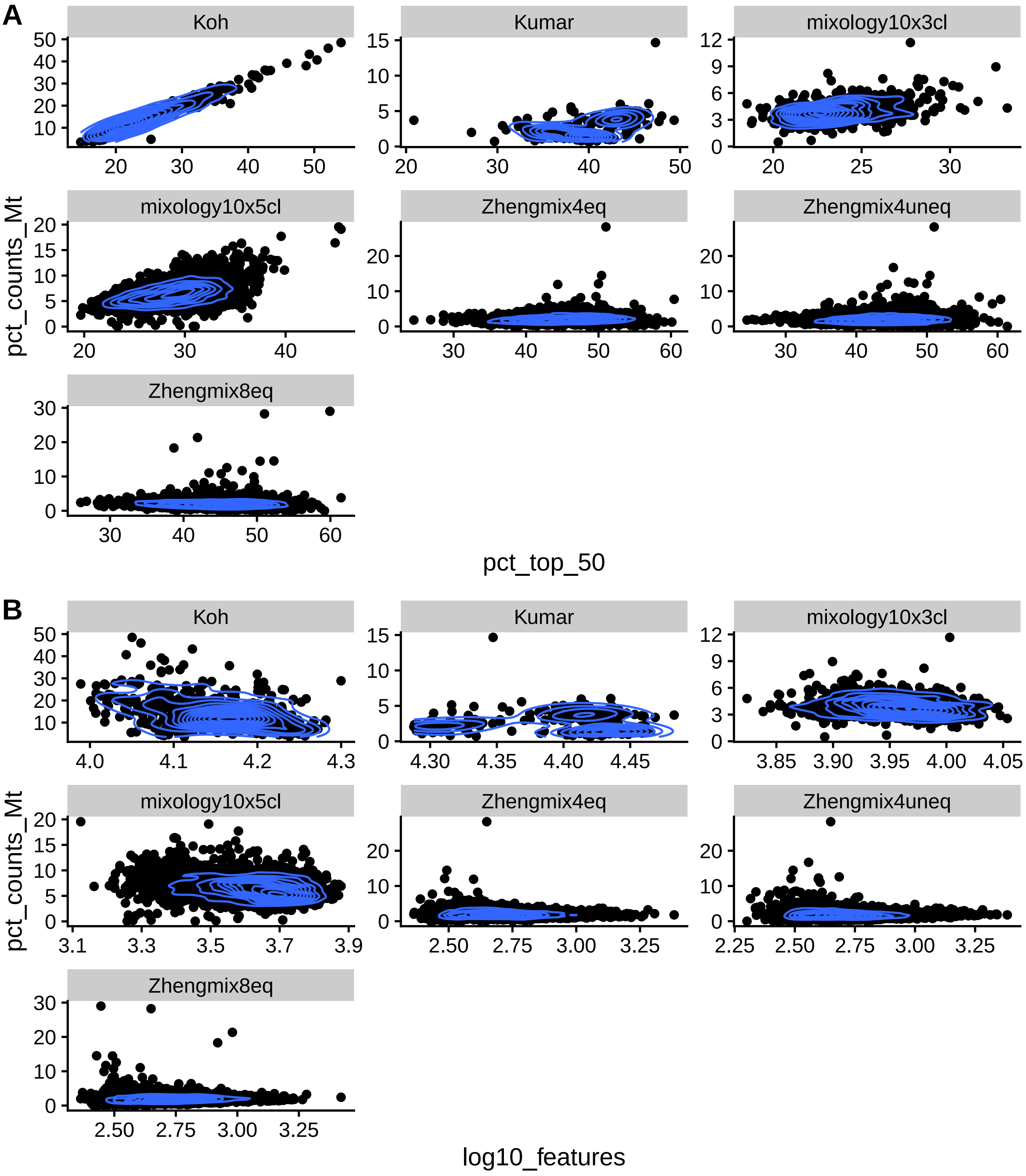

Fig S6

Relationship between selected cell-level QC metrics.

Fig S7

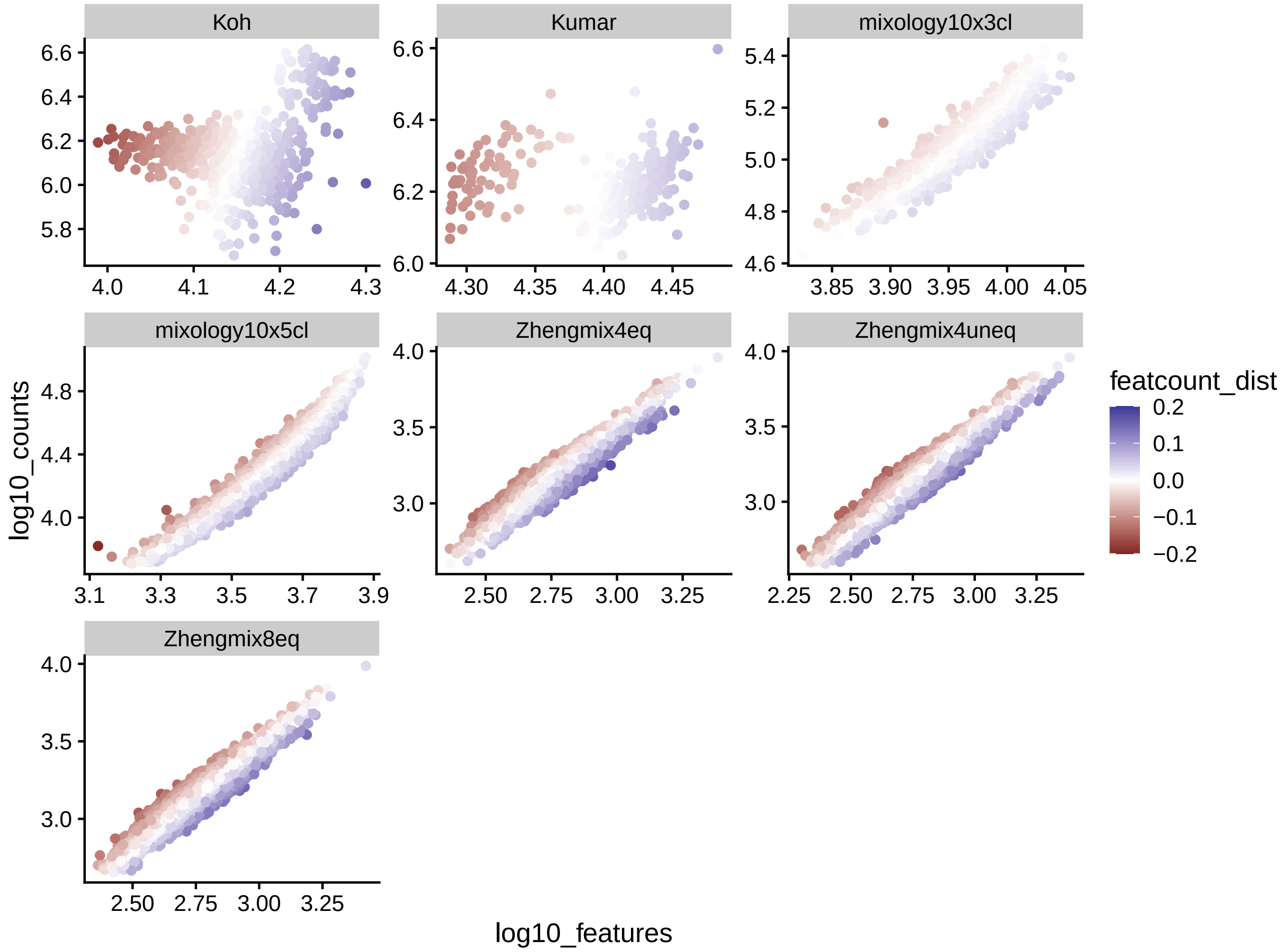

Fig S7

There is a tight relationship, in 10x datasets (i.e. not the `Koh` and `Kumar` datasets), between the total counts of a cell and its number of detected features. We therefore include, among control variables, deviation from this ratio.

Fig S8

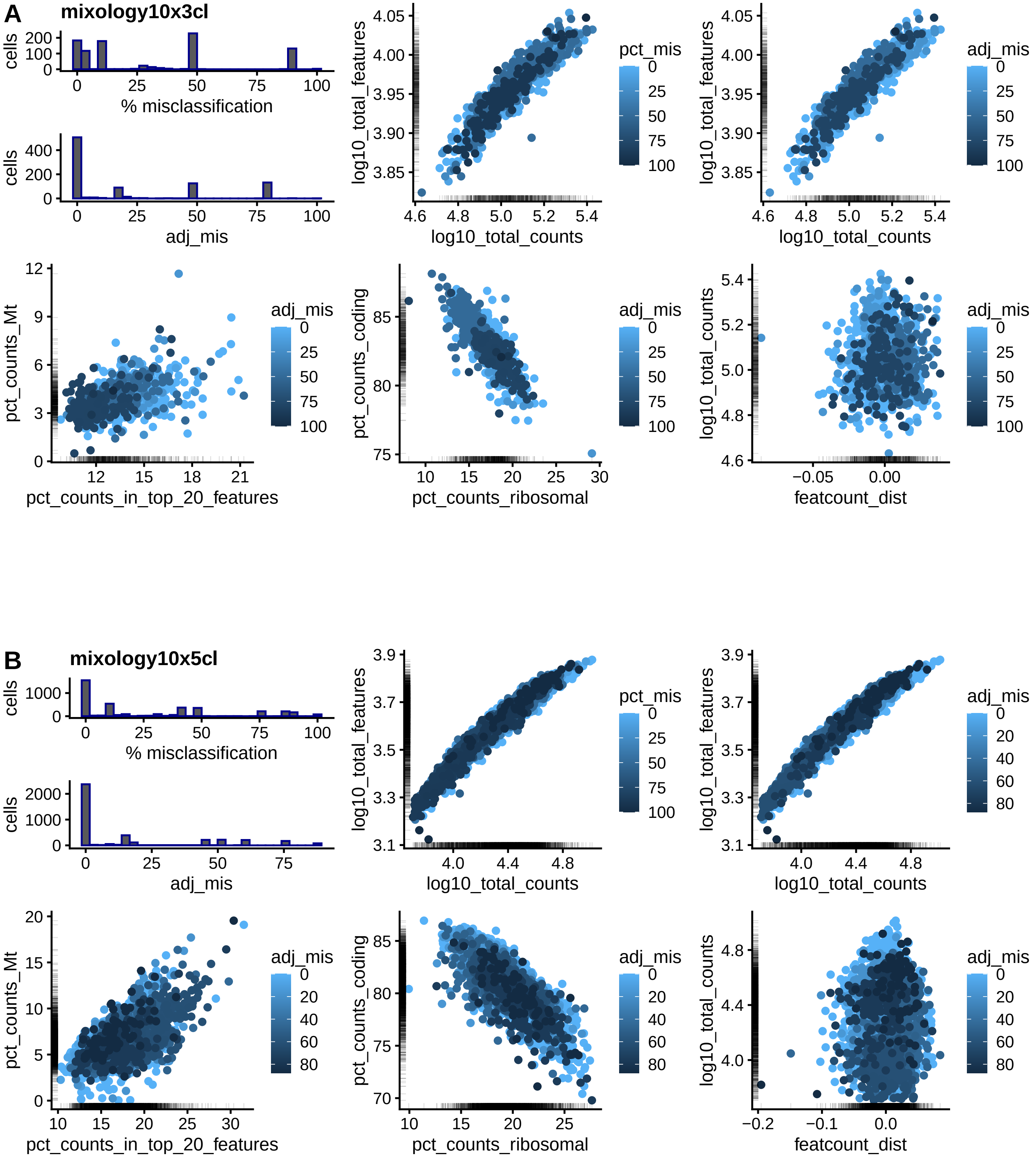

Fig S8

Relationship between various cellular properties and the frequency of cluster mis-assignment for the mixology10x3cl (A) and mixology10x5cl (B) datasets. The percentage of misclassification refers to the frequency with which a given cell is assigned the wrong cluster (using the Hungarian algorithm for cluster matching) across several hundred clustering runs with varying parameters. Since some subpopulations tend to be more misclassified than others, the adjusted rate of misclassification (`adj_mis`) is substracted for the subpopulation median misclassification rate.

Fig S9

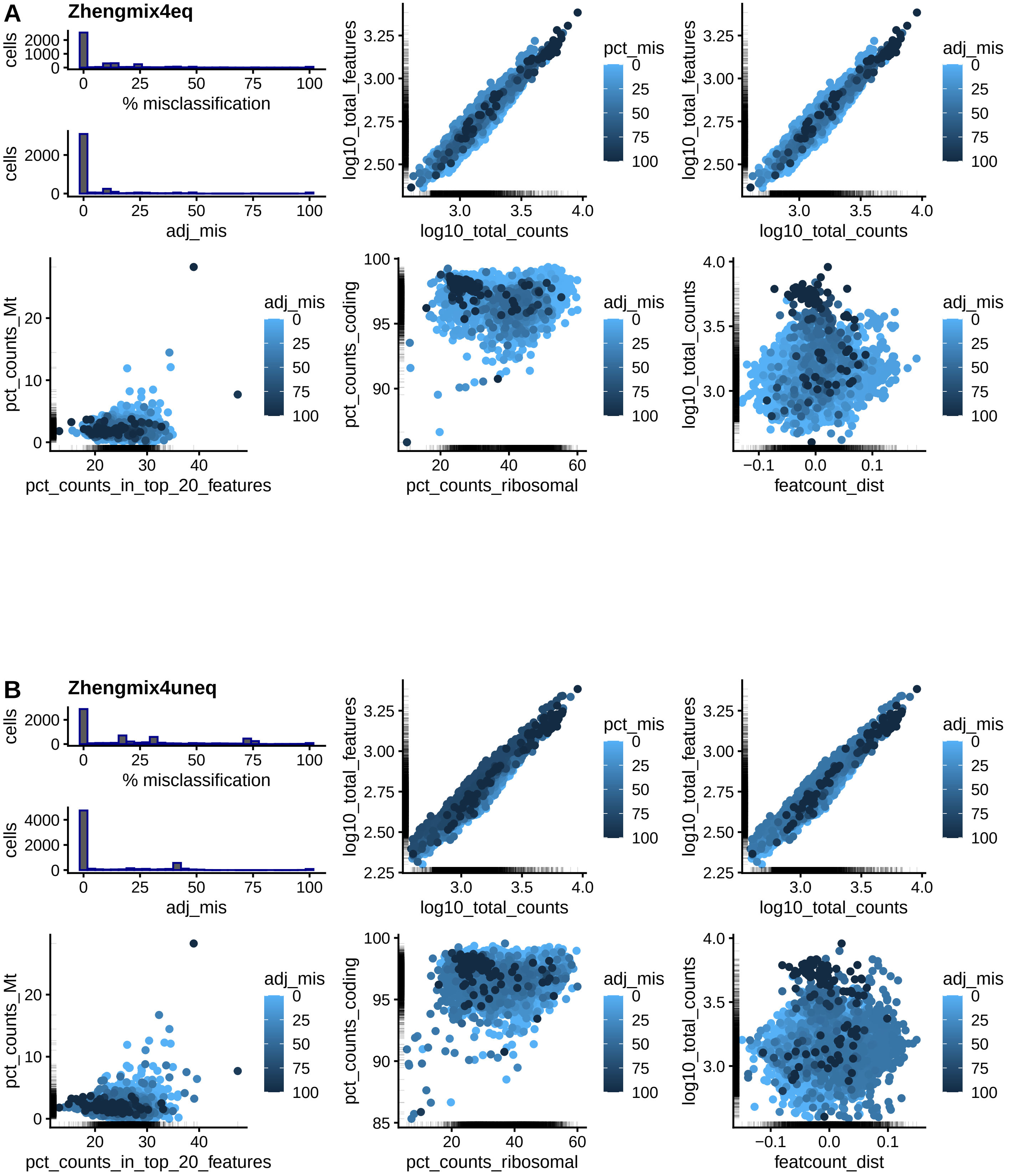

Fig S9

Relationship between various cellular properties and the frequency of cluster mis-assignment for the Zheng equal (A) or unequal (B) mixtures of four cell types. See Supplementary Figure 8 for more information. The only clear pattern is that cells with a high number of reads or features tend to have a higher misclassification rate.

Fig S10

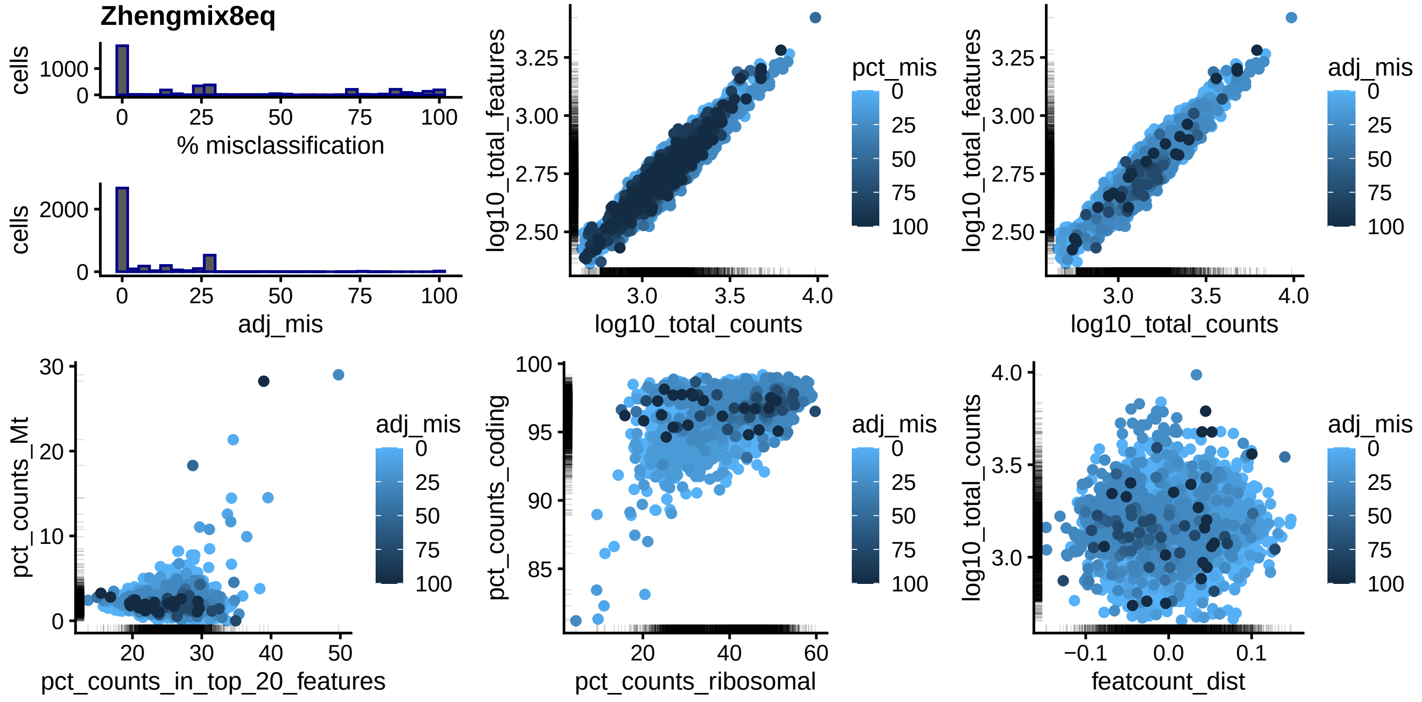

Fig S10

Relationship between various cellular properties and the frequency of cluster mis-assignment for the Zheng mixture of 8 cell types. See Supplementary Figure 8 for more information.

Fig S11

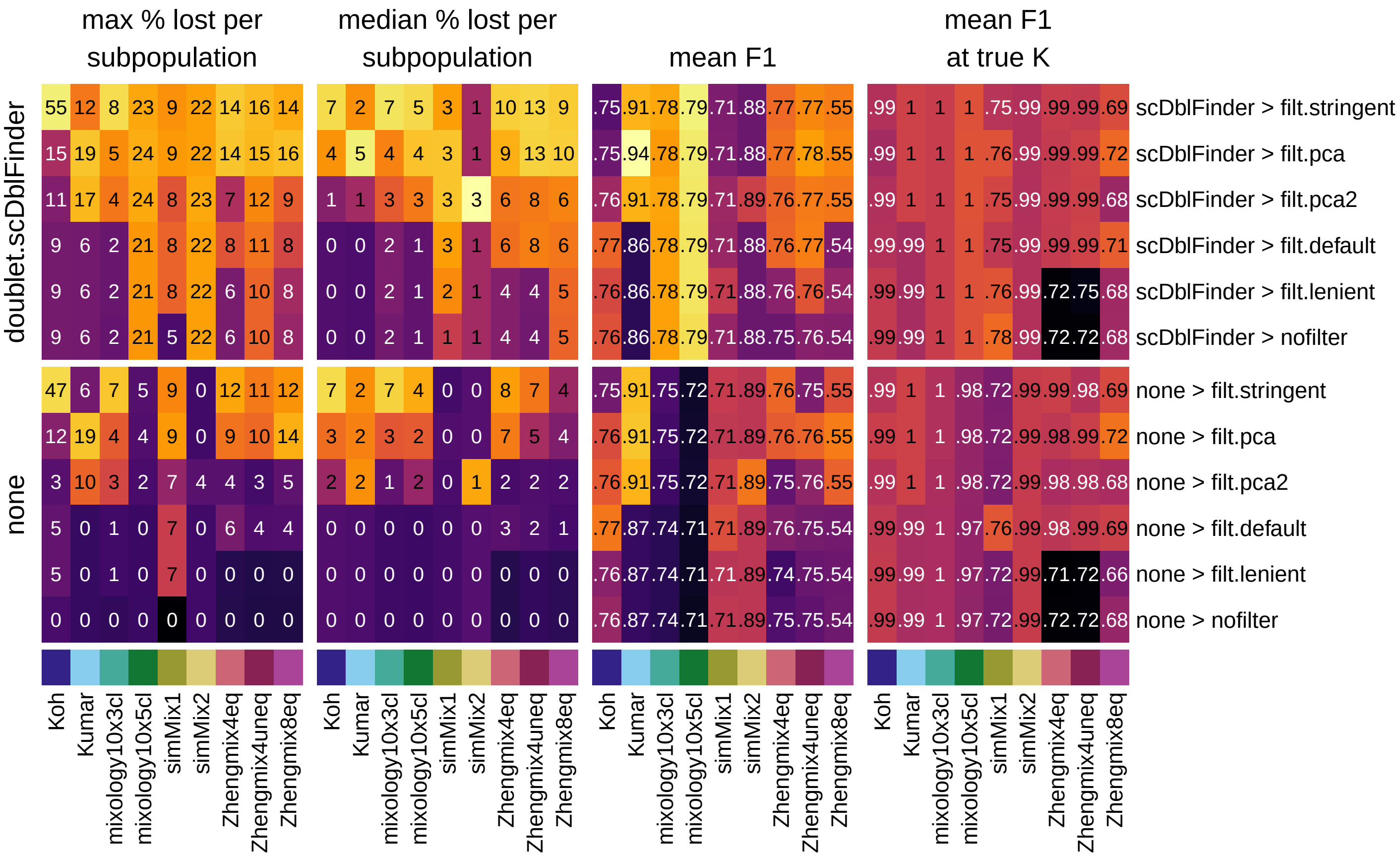

Fig S11

Mean clustering F1 score per subpopulation, mean F1 at true number of clusters, as well as maximum and median proportion of excluded cells per subpopulation across various filtering strategies. Doublet removal generally improves clustering accuracy with relatively mild increases exclusion rates, even in datasets that do not have heterotypic doublets. Stringent distribution-based filtering creates large cell type biases.

Fig S12

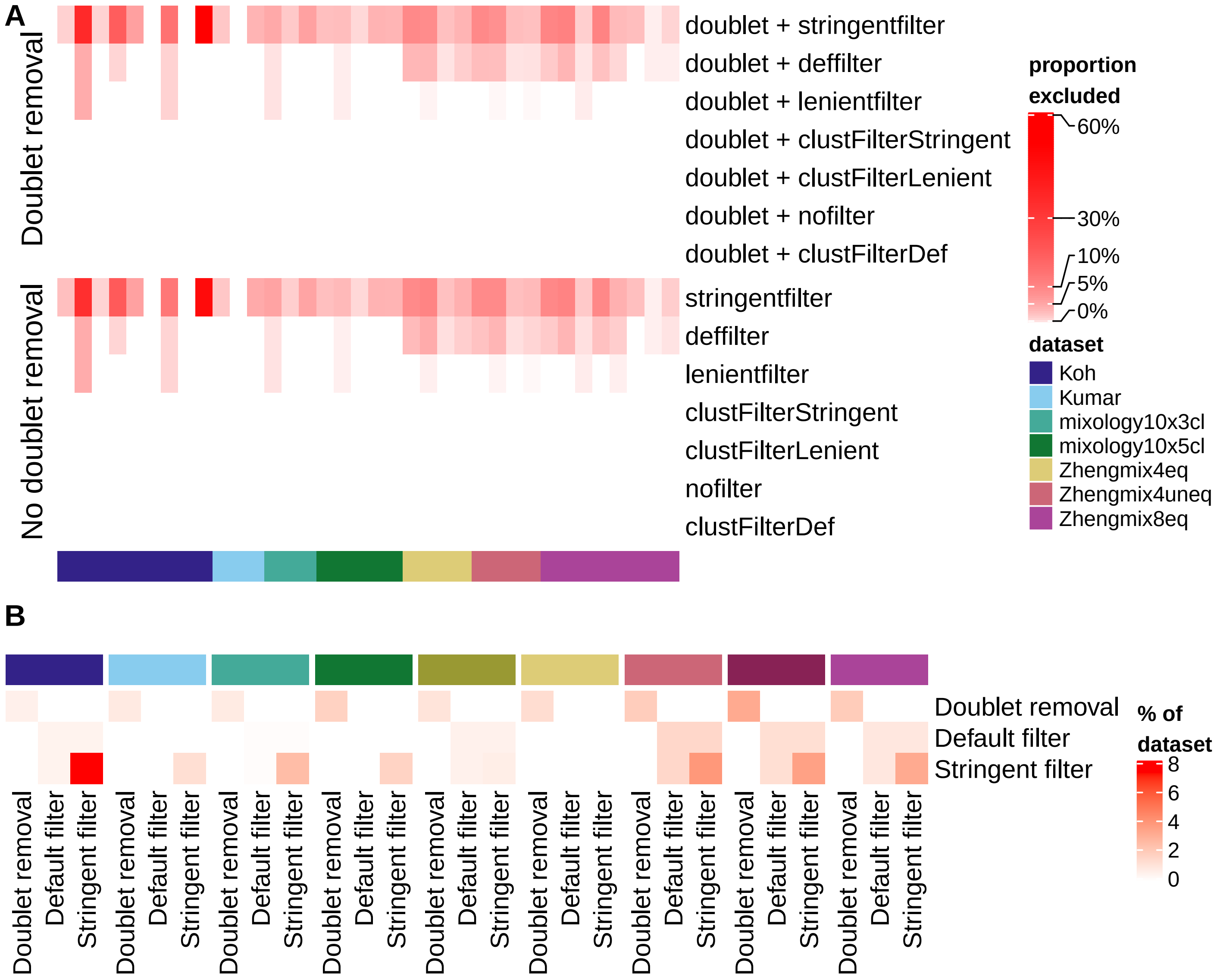

Fig S12

A: Proportion of cells filtered out by subpopulation. Applying the same filters in a cluster-wise fashion (using `scran::quickCluster`, and desginated here with `clustFilter*`) leads to virtually no cell exclusion. The color-mapping is square-root transformed to improve the visibility of differences at low proportions. **B:** Overlap between cells excluded by doublet removal (`scDblFinder`) and those excluded by MAD-based filters (without doublet removal; the filters are described in the methods), expressed as a proportion of the dataset. The cells excluded as doublets do not tend to be excluded by (even stringent) MAD-based filtering.

Fig S13

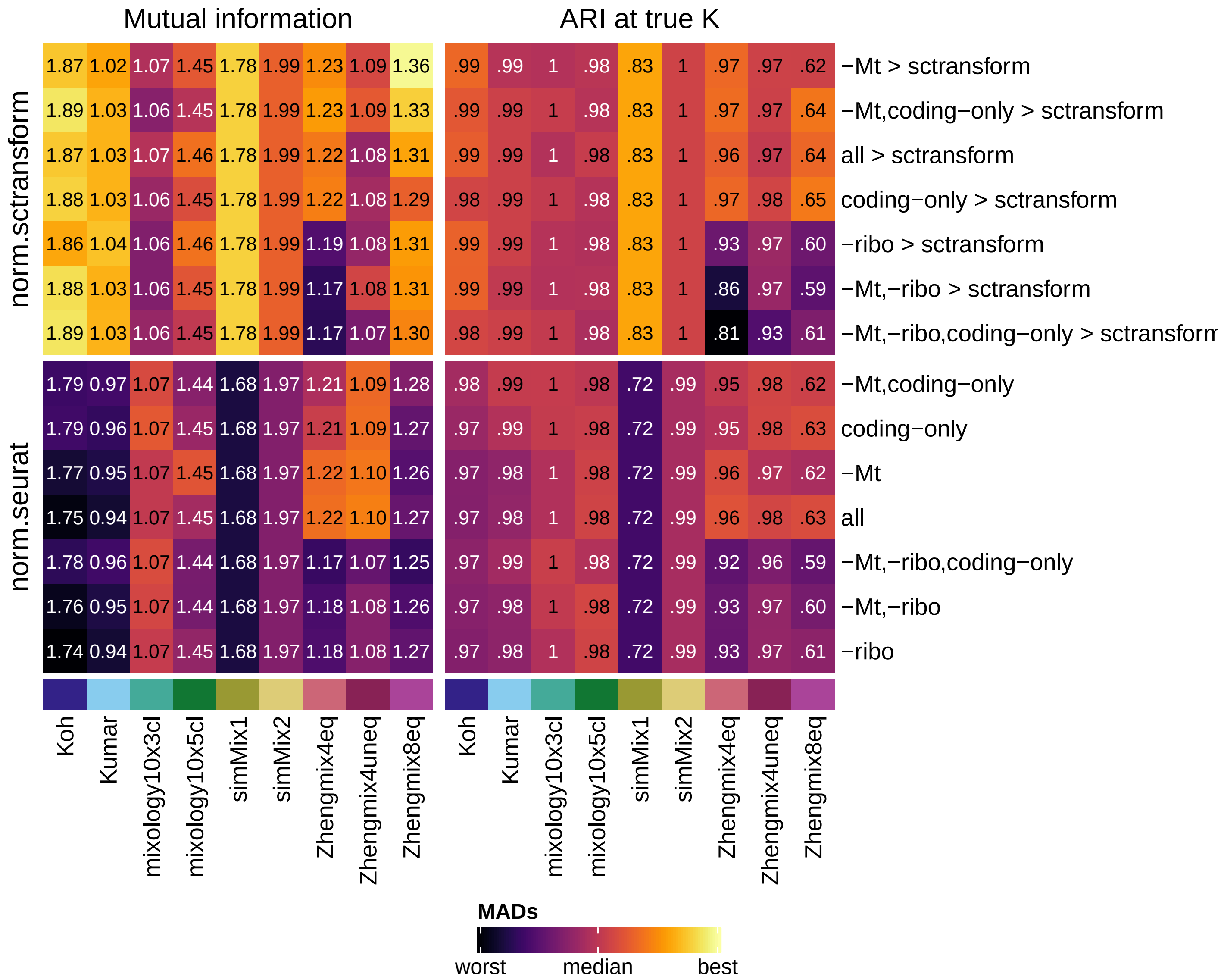

Fig S13

Impact of restricting the type of features used on the Mutual Information (MI, left) and Adjusted Rand Index (ARI, right) of the clustering. `all` indicates that all features were used, `-Mt` stands for the exclusion of mitochondrial genes, `-ribo` the exclusion of ribosomal genes, and `coding-only` a restriction to protein-coding genes. The features were filtered out prior to normalization.

Fig S14

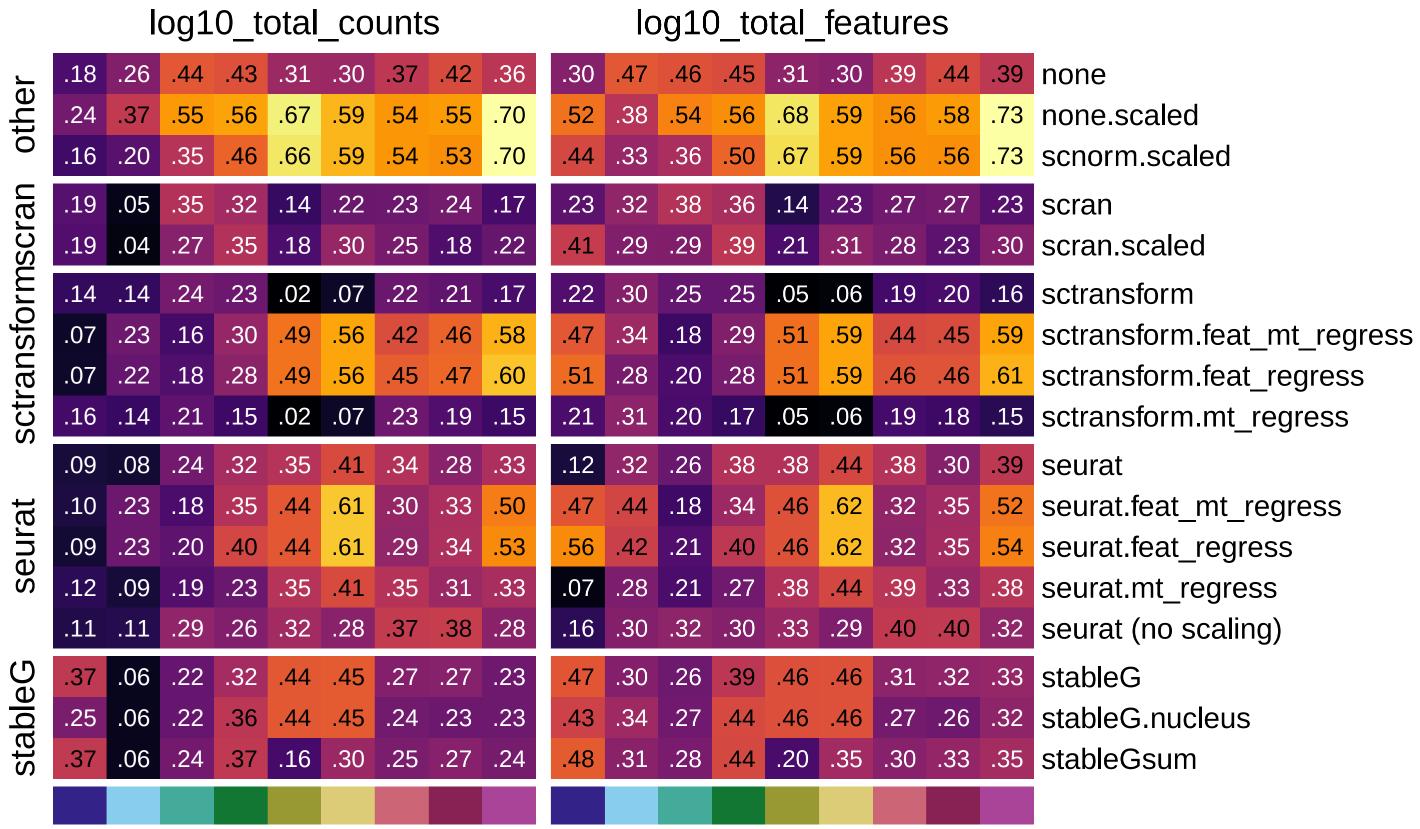

Fig S14

Mean per-subpopulation absolute correlation of the first 5 components with library size (left) and the number of detected features (right) across normalization procedures.

Fig S15

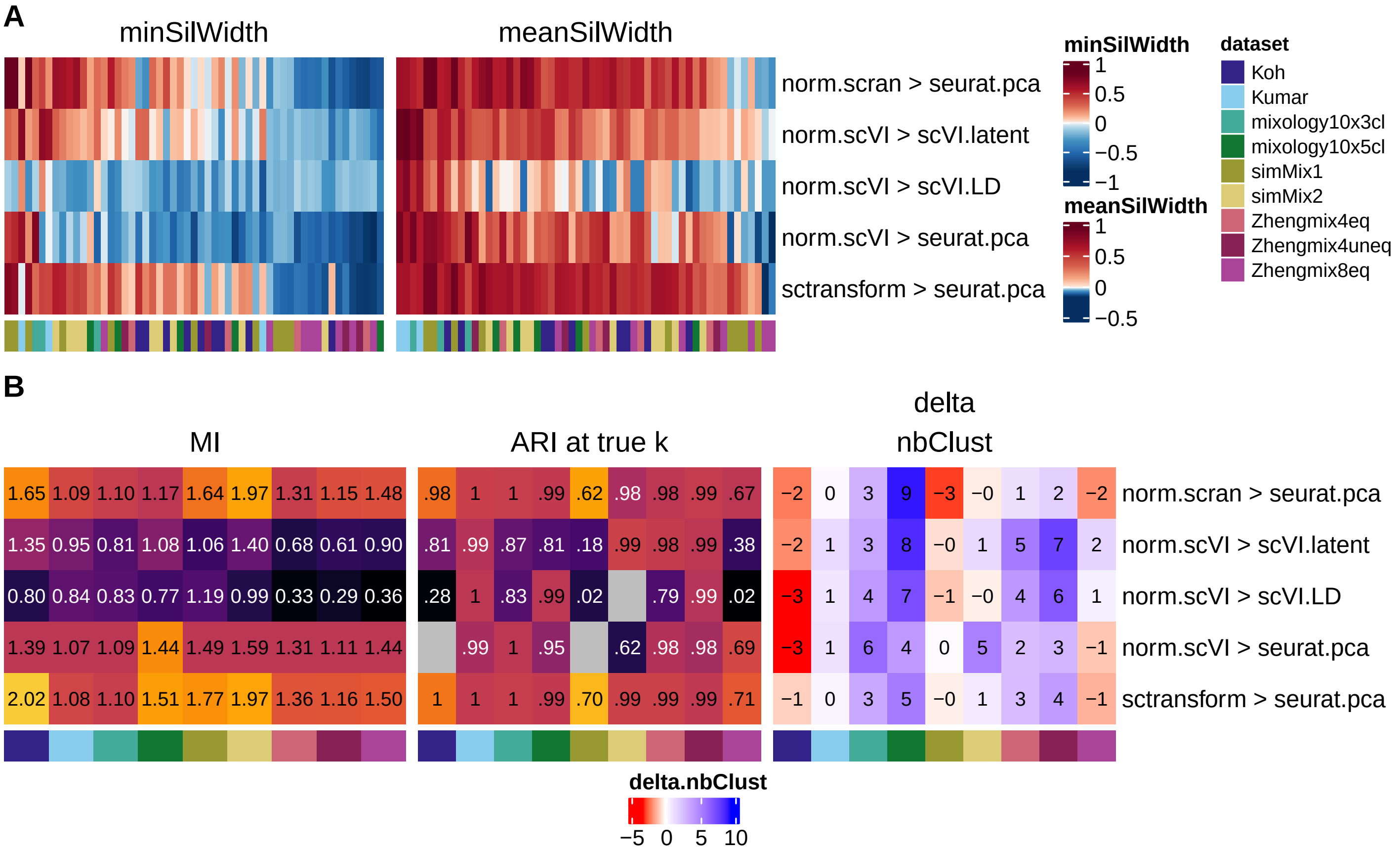

Fig S15

scVI evaluation. A: Average silhouette width per subpopulation using either sctransform, scran or scVI normalization followed by Seurat PCA, or the scVI latent space (latent) or imputed values (LD) of the linear decoder. **B:** Clustering accuracy across the same methods followed by Seurat clustering.

Fig S16

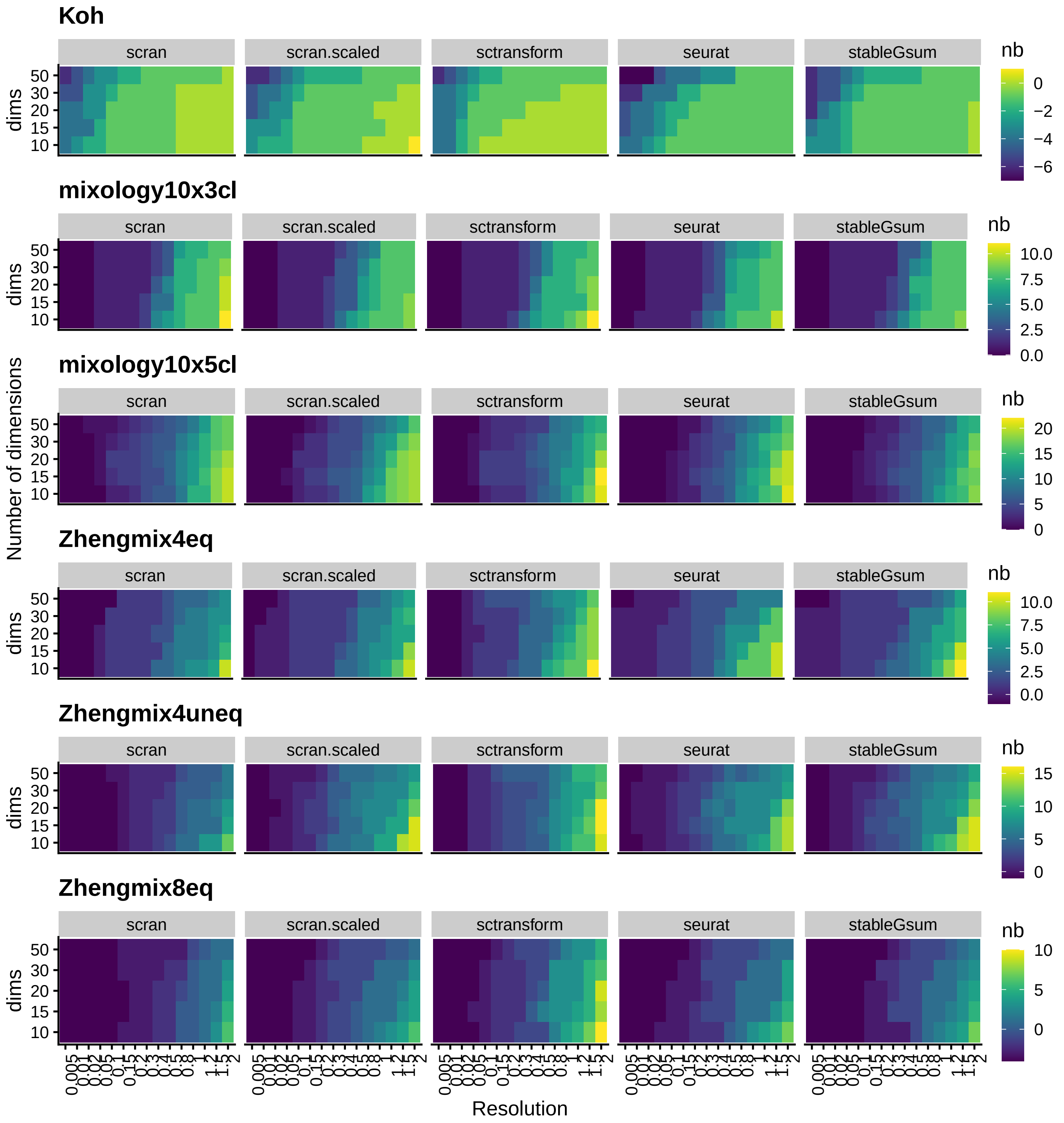

Fig S16

Mean difference between the number of detected clusters and the number of real subpopulations, depending on the normalization method, the resolution and the number of dimensions used. The Kumar dataset is not shown here due to a lack of variation in the number of clusters detected. A rough ANOVA on `nbClusters~dataset+norm+dims+resolution` suggests that seuratvst (sctransform) is associated with a higher number of clusters (p~0).

Fig S17

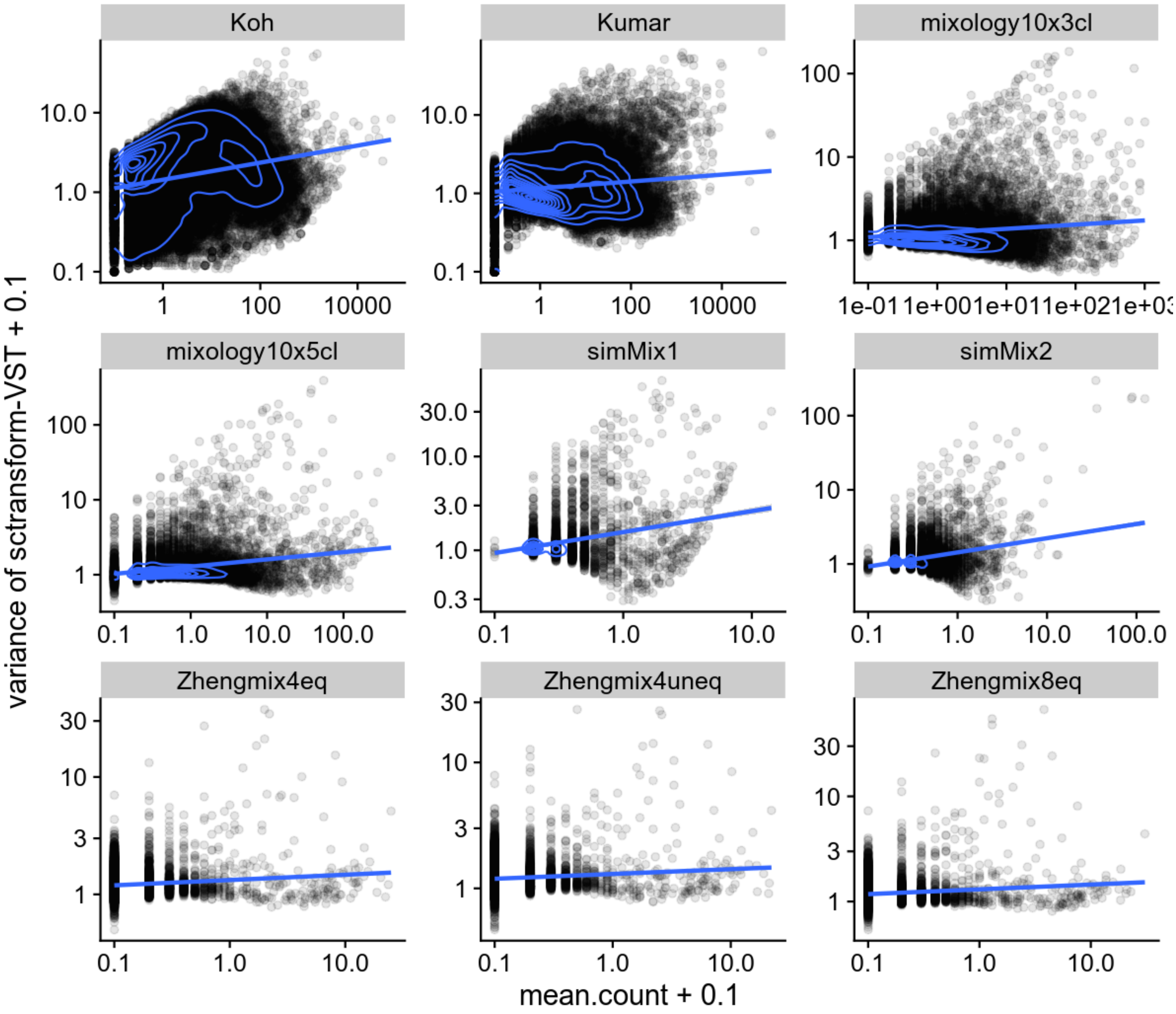

Fig S17

Relationship of the variance with mean count after `sctranform`'s variance stabilizing transformation.

Fig S18

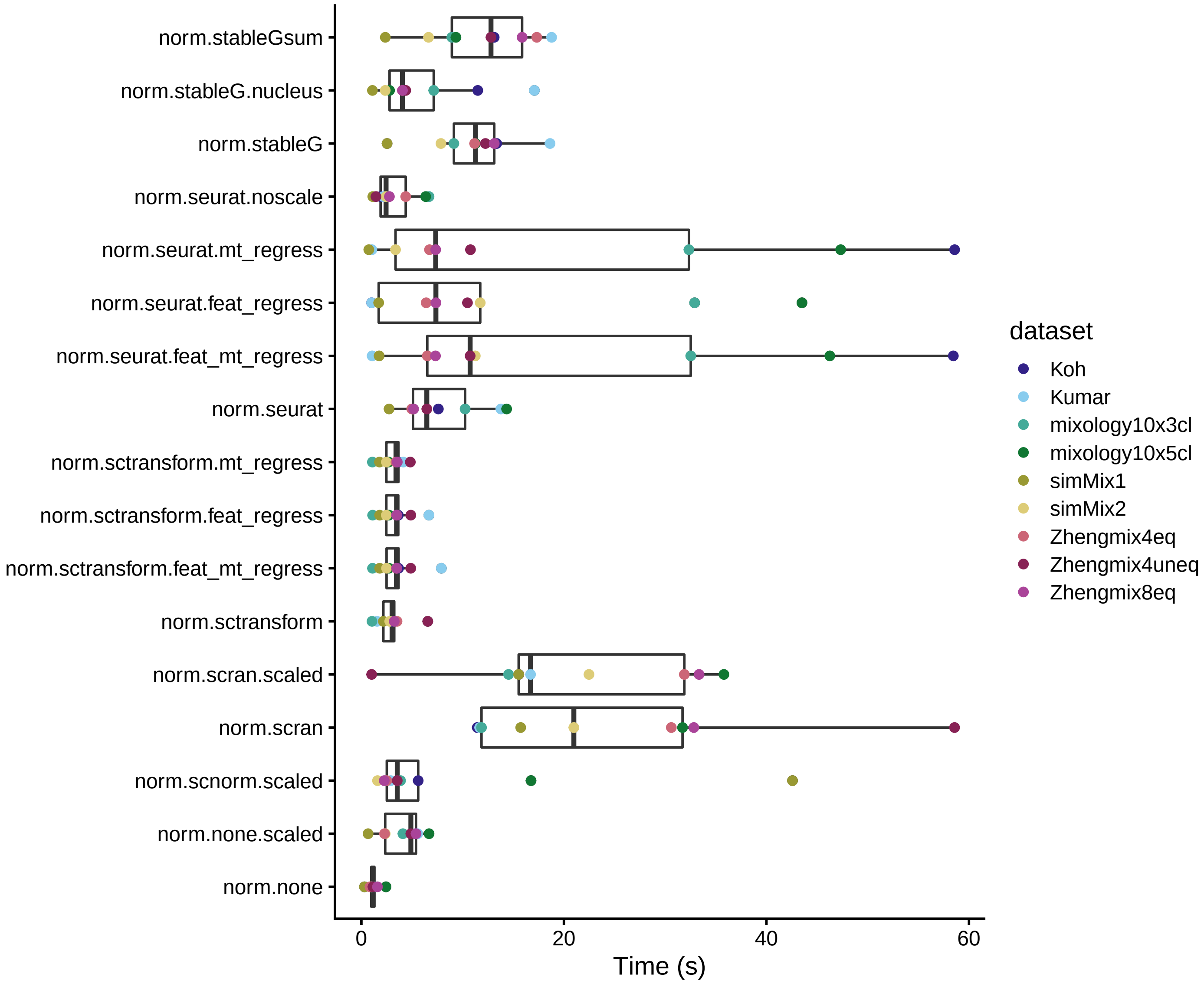

Fig S18

Running time of the normalization methods.

Fig S19

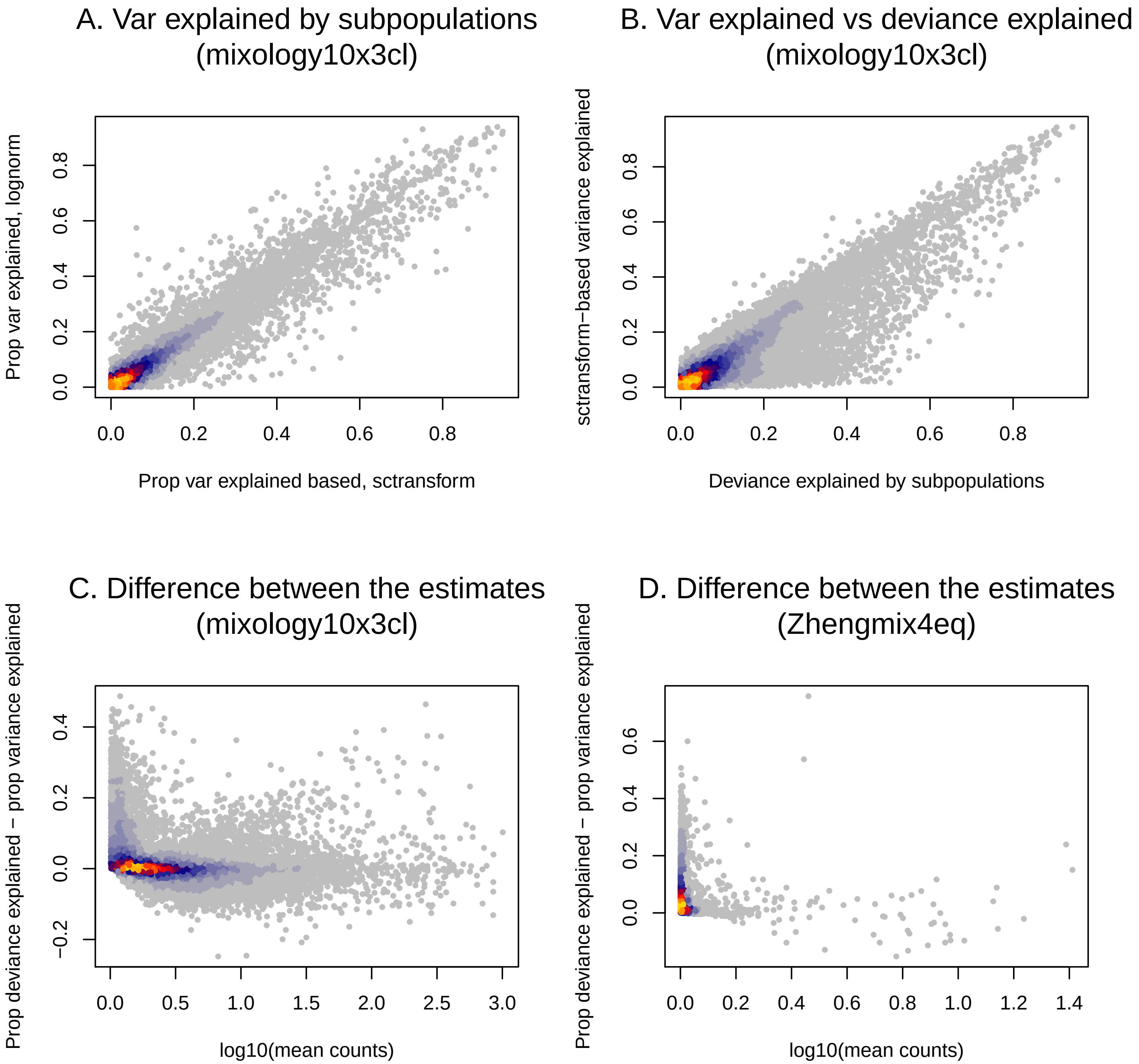

Fig S19

A: Comparison of the gene-wise proportion of variance explained by real subpopulations based on Seurat's standard log normalization and on `sctransform` variance-stabilizing transformation. Across 10x datasets, there is a good agreement between the two, the correlation ranging between 0.92 and 0.97. **B:** There is also a good agreement between *variance* and *deviance* explained, with some genes having a higher deviance explained. **C-D:** Relationship between mean expression and the difference between the proportion of deviance explained and the proportion of variance explained in two datasets. Genes that have a higher proportion of the deviance explained than of the variance explained are generally the lowly-expressed ones.

Fig S20

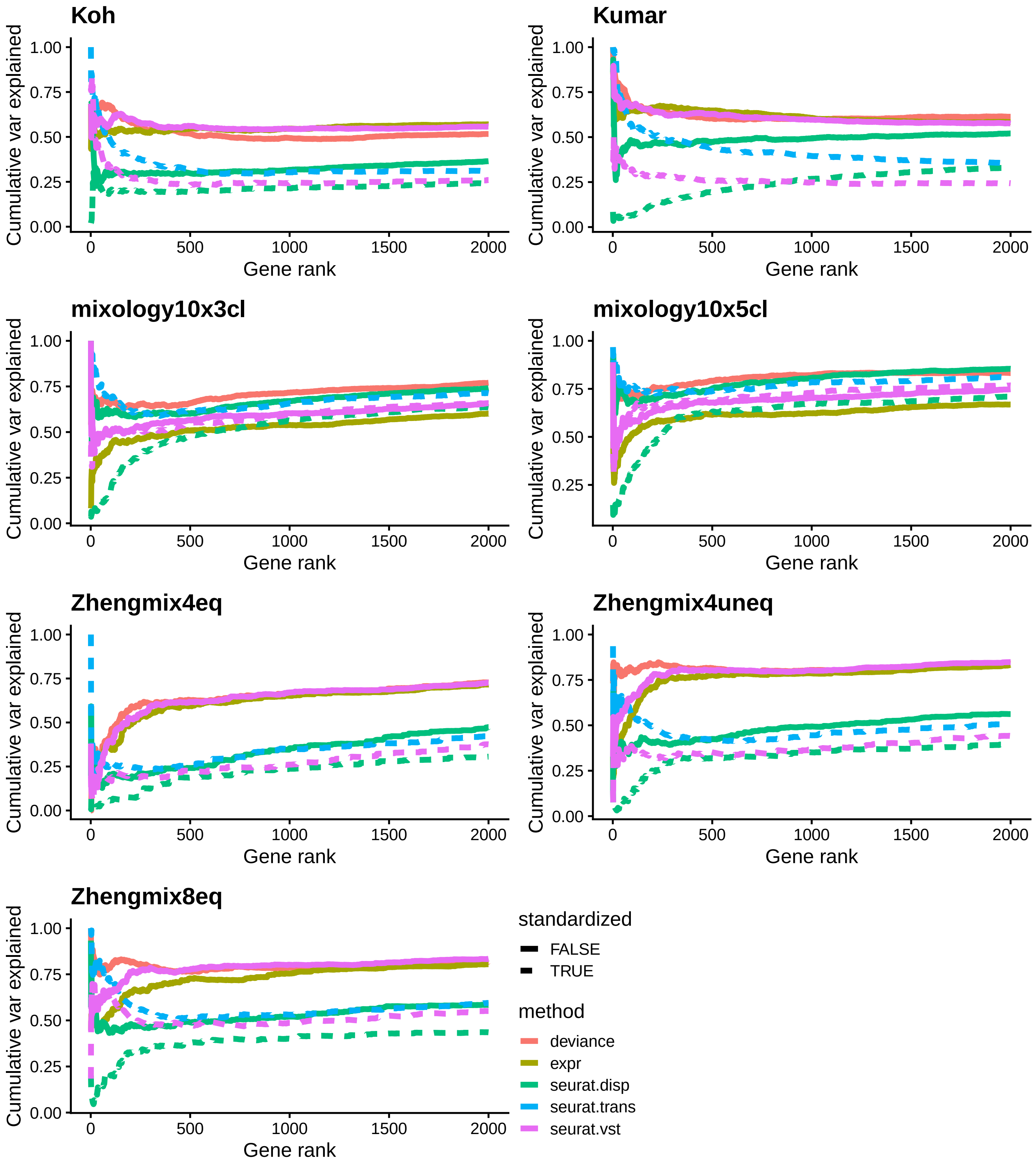

Fig S20

Proportion of the cumulative *variance* explained by real subpulations that is retrieved through the selection. For each gene, we compute the proportion of the variance explained by real subpopulations. For each rank X, we sum this proportion for the X genes selected by a given method, and divide it by the sum when selecting the X genes with the highest variance explained. An ideal selection would therefore be a horizontal line at 1.

Fig S21

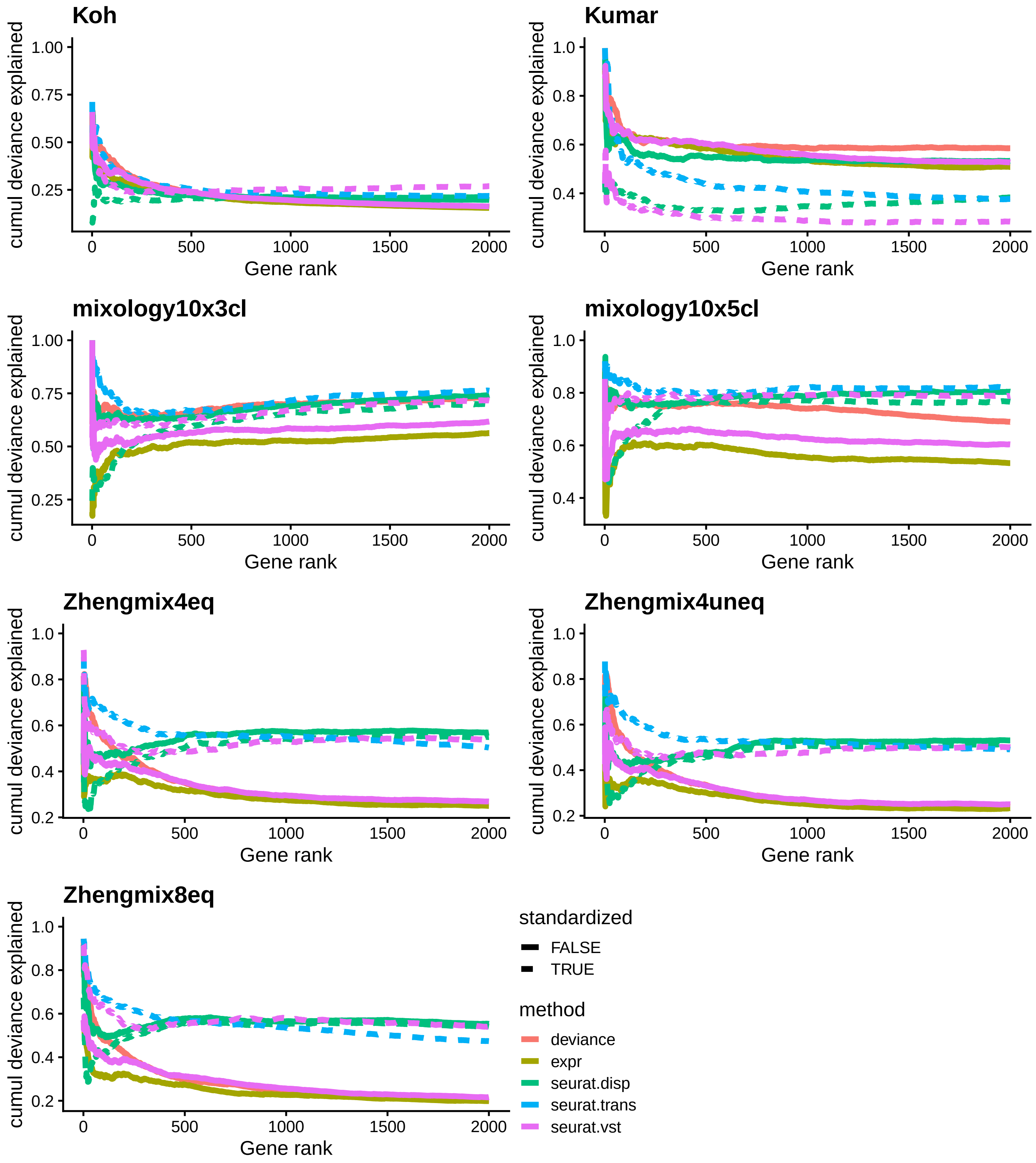

Fig S21

Proportion of the cumulative *deviance* explained by real subpulations that is retrieved through the selection. For each gene, we compute the proportion of the variance explained by real subpopulations. As for Supplementary Figure 20, except using deviance explained.

Fig S22

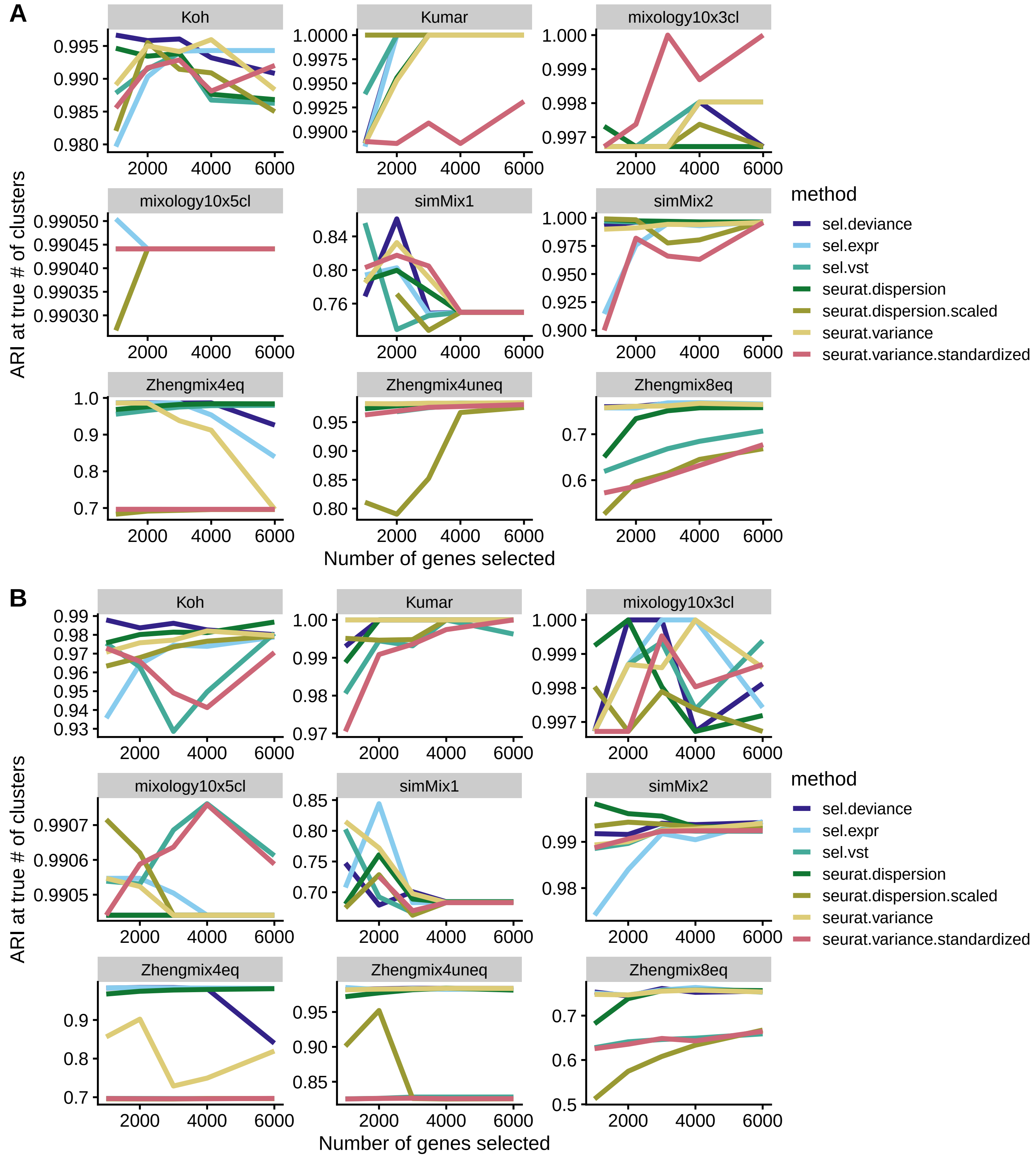

Fig S22

Clustering accuracy according to the number of genes selected using various ranking/selection methods. **A:** Based on sctransform, **B:** Based on standard Seurat normalization.

Fig S23

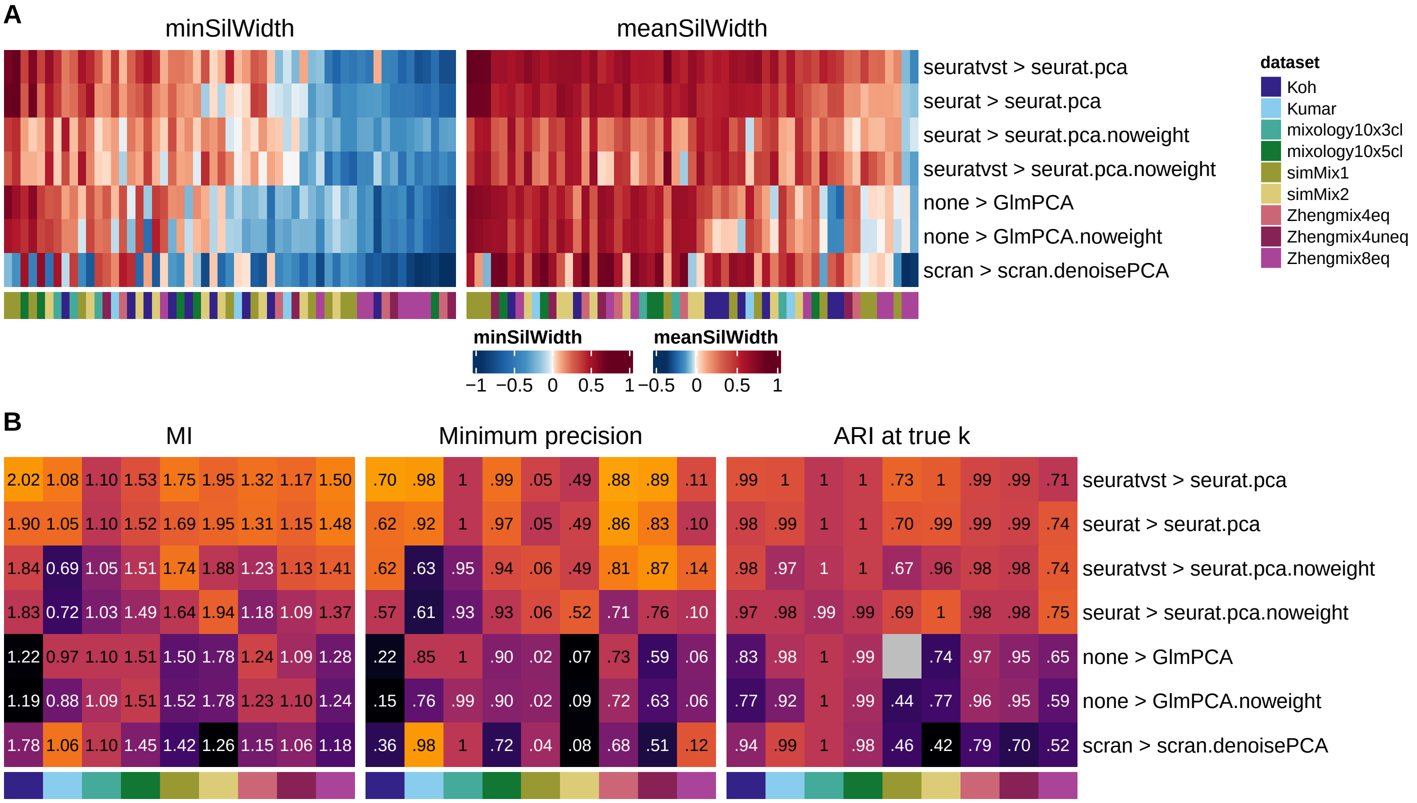

Fig S23

Evaluation of common dimensionality reduction methods. A: Minimum (left) and average (right) silhouette width per subpopulation resulting from combinations of normalization and dimension reductions. **B:** Clustering accuracy, measured by mutual information (MI), minimum subpopulation precision, and adjusted Rand index (ARI) at the true number of clusters.

Fig S24

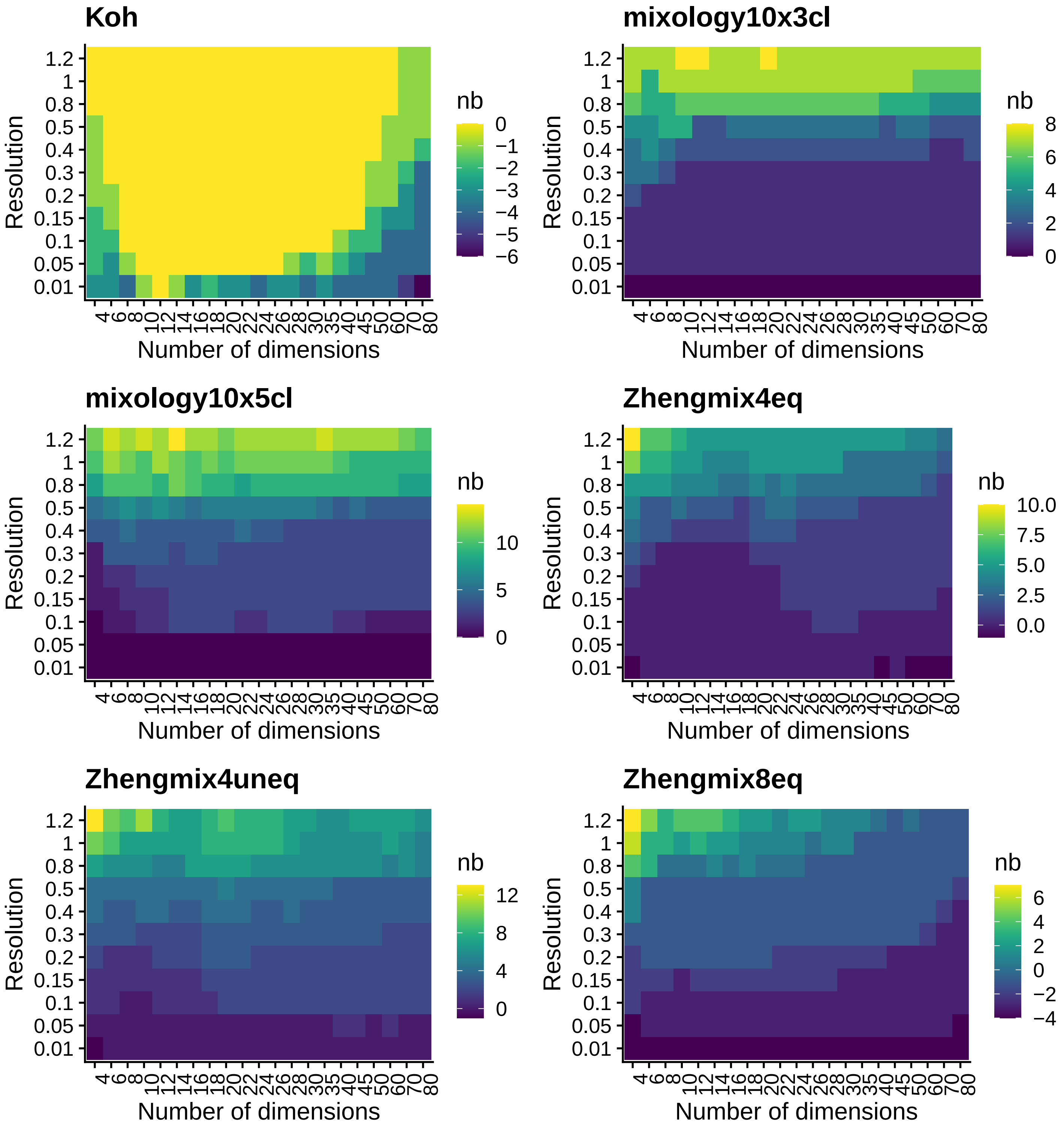

Fig S24

Mean difference between the number of detected clusters and the number of real subpopulations, depending on the resolution and number of dimensions used. Based on sctransform and seurat PCA. Increasing the number of dimensions tends to decrease the number of identified clusters, especially at resolutions around the default value.

Fig S25

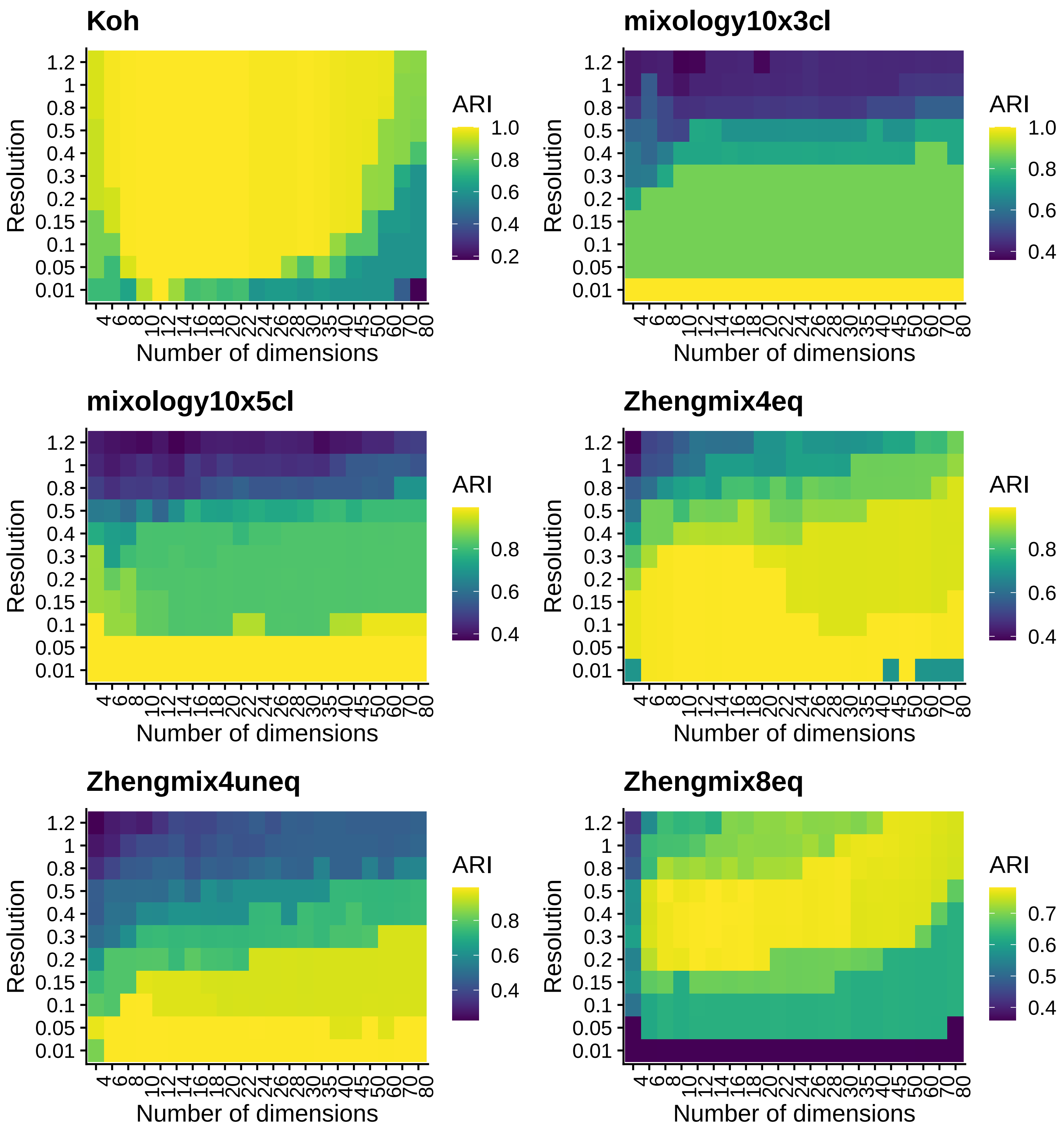

Fig S25

Adjusted Rand Index of clustering depending on the resolution and number of dimensions used. Based on sctransform and seurat PCA.

Fig S26

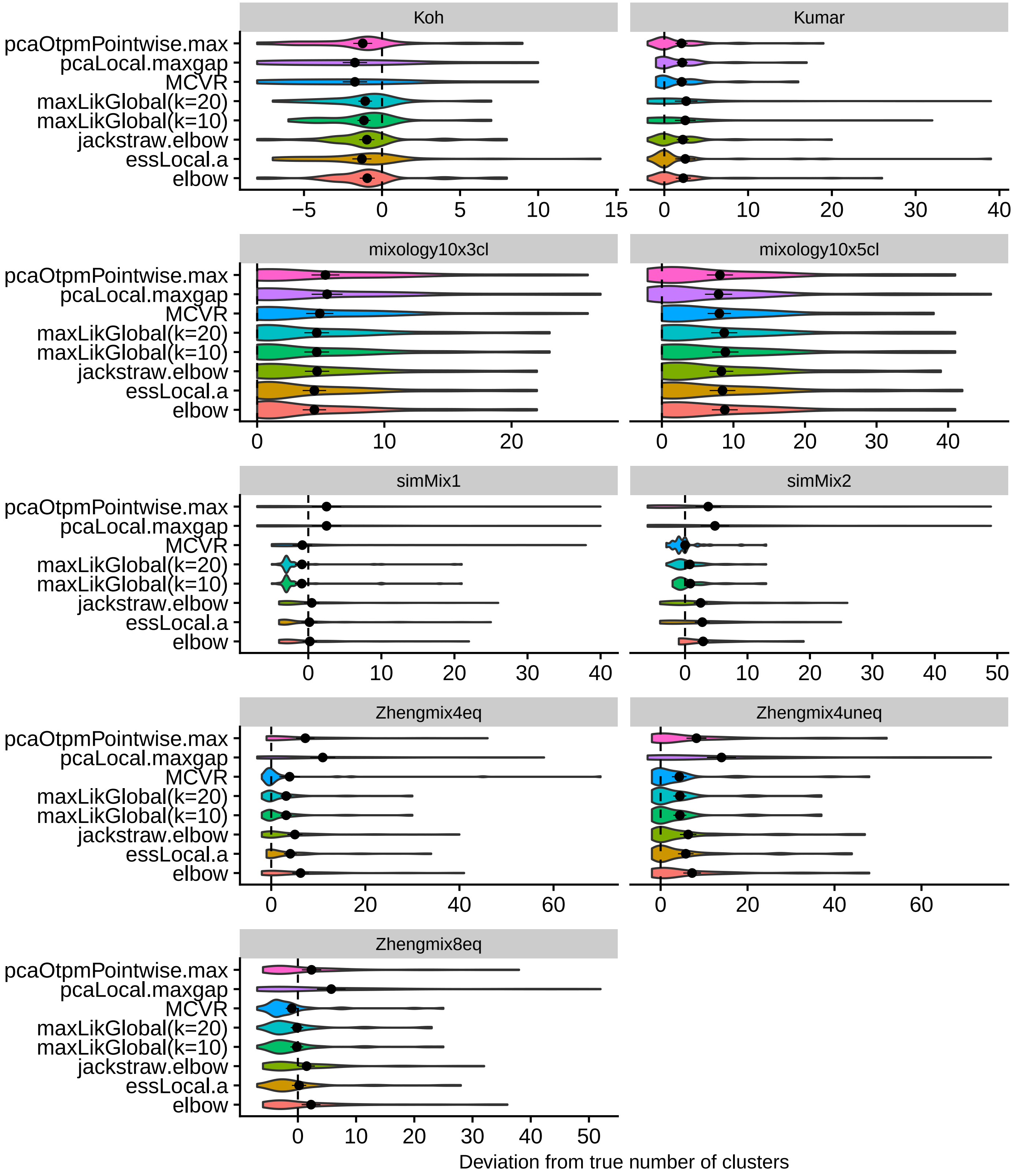

Fig S26

Deviation from the true number of clusters using different number of principal components (based the indicated dimensionality estimates) of the same Seurat-based PCA. Default pipeline parameters were used for the other steps, and the distributions represent the different resolutions of Seurat clustering. Across datasets, MCVR and maxLikGlobal appear to depart less from the true number of clusters.

Fig S27

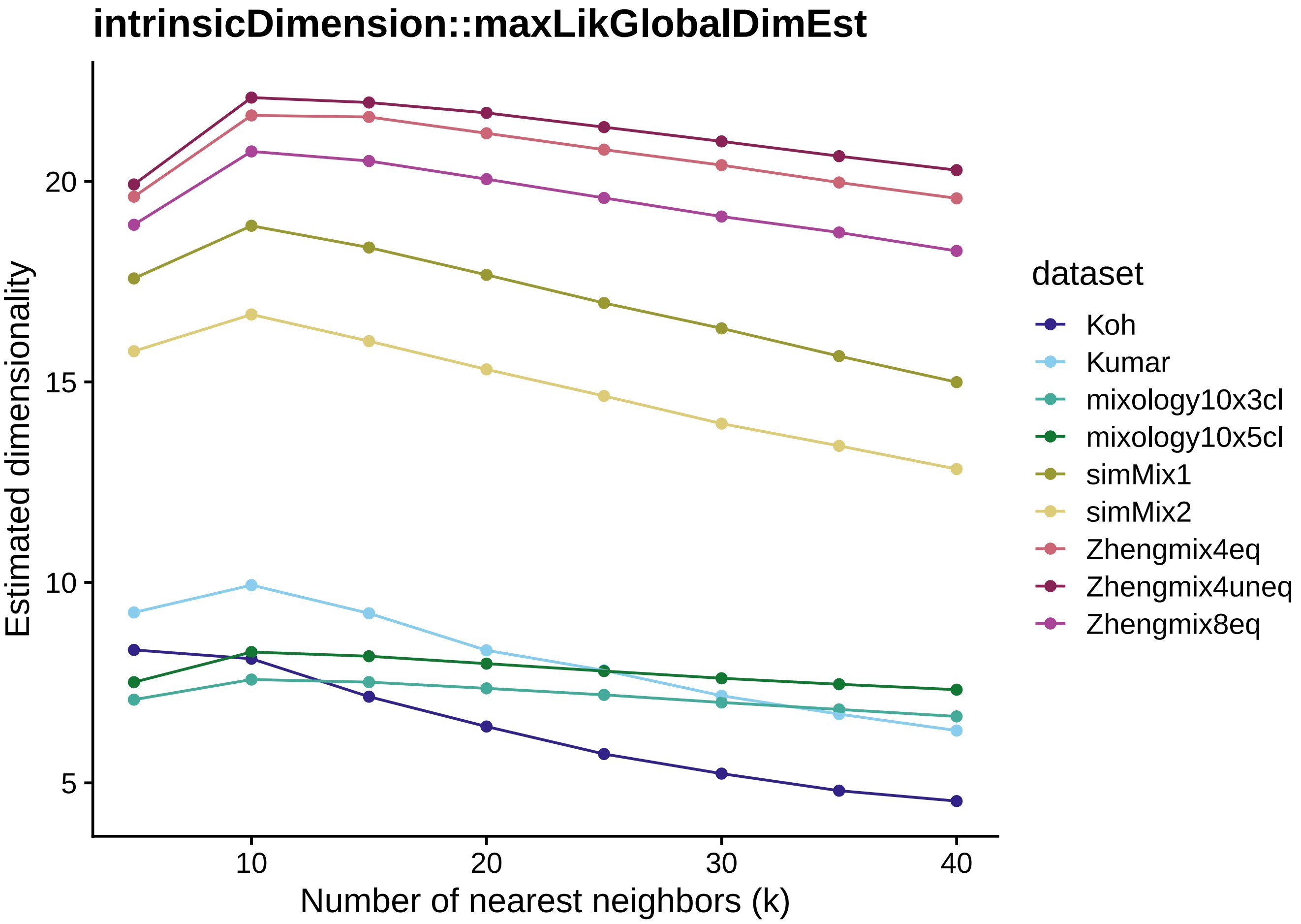

Fig S27

Estimates of dimensionality by the `intrinsicDimension::maxLikGlobalDimEst` method using various 'reasonable' numbers of nearest neighbors (`k` parameter).

Fig S28

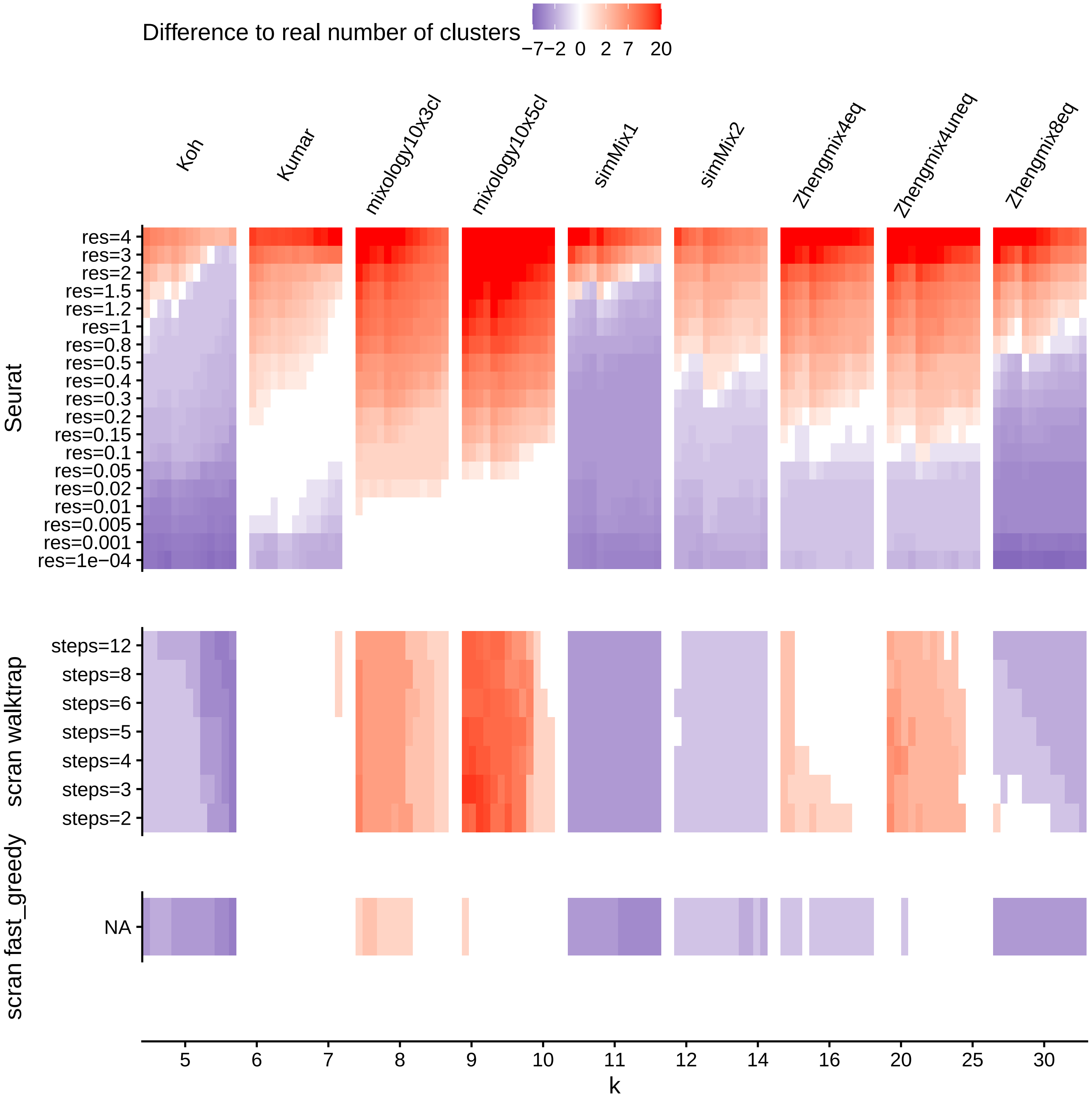

Fig S28

Difference between the number of detected clusters and the number of real subpopulations according to different clustering paramters.

Fig S29

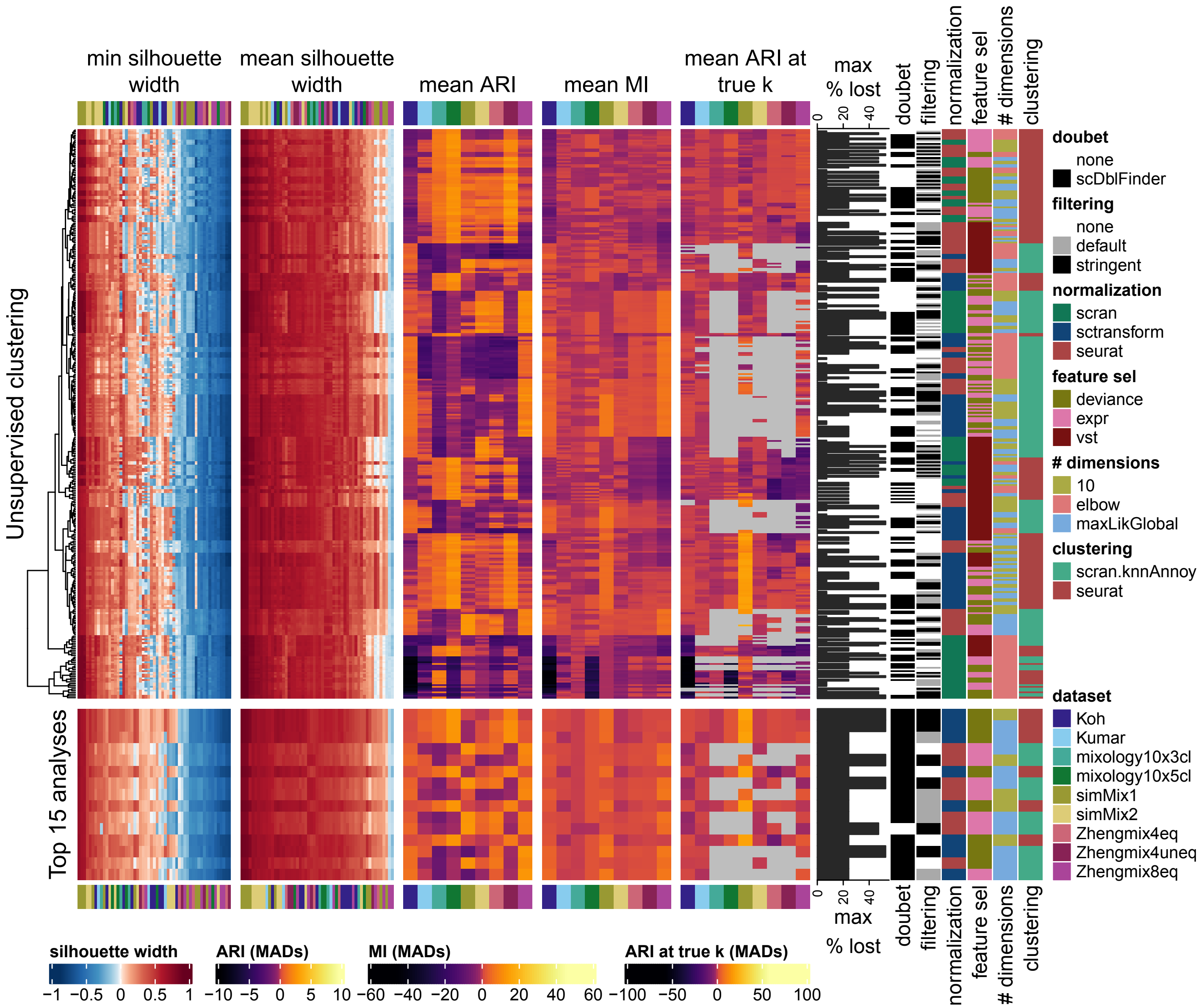

Fig S29

Overview of the combination of main alternative parameters. Unsupervised clustering of the results (above) and top results (below). The color-mapping schemes are the same as described in the main figures.

Fig S30

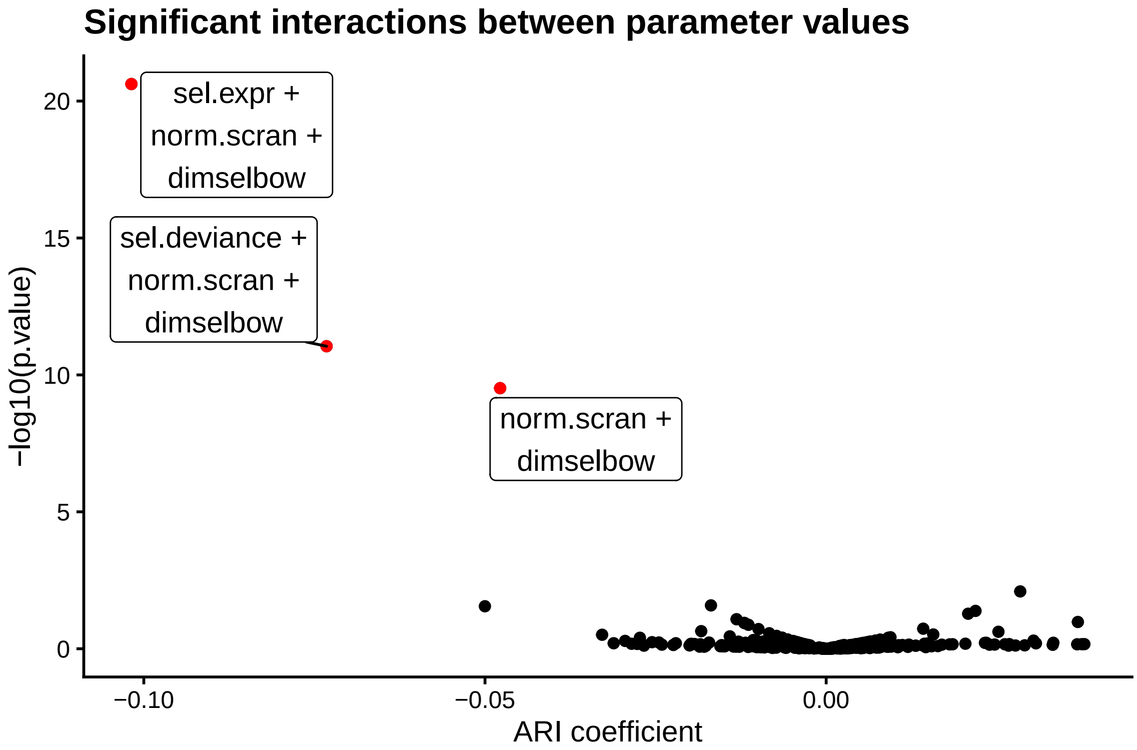

Fig S30

Estimated coefficient and -log10 significance of all interaction coefficients in the linear model defined by

`ARI~dataset*resolution+doubletmethod*sel*filt*norm*clustmethod*dims`

Those terms significant at a FDR < 0.05 are highlighted. The top interaction terms are:

	Estimate	SE	p.value	FDR
sel.expr:norm.scran:dimselbow	-0.102	0.011	0.000	0.00
sel.deviance:norm.scran:dimselbow	-0.073	0.011	0.000	0.00
norm.scran:dimselbow	-0.048	0.008	0.000	0.00
sel.expr:norm.sctransform:dimselbow	0.028	0.011	0.008	0.63
norm.sctransform:dimselbow	-0.017	0.008	0.026	1.00
clustmethodclust.scran.knnAnnoy:dimselbow	-0.050	0.023	0.028	1.00
sel.deviance:norm.sctransform:dimselbow	0.022	0.011	0.041	1.00
filtfilt.stringent:norm.scran:dimselbow	0.021	0.011	0.053	1.00
sel.expr:dimselbow	-0.013	0.008	0.083	1.00
clustmethodclust.scran.knnAnnoy:dimsmaxLikGlobal	0.037	0.023	0.105	1.00

Fig S31

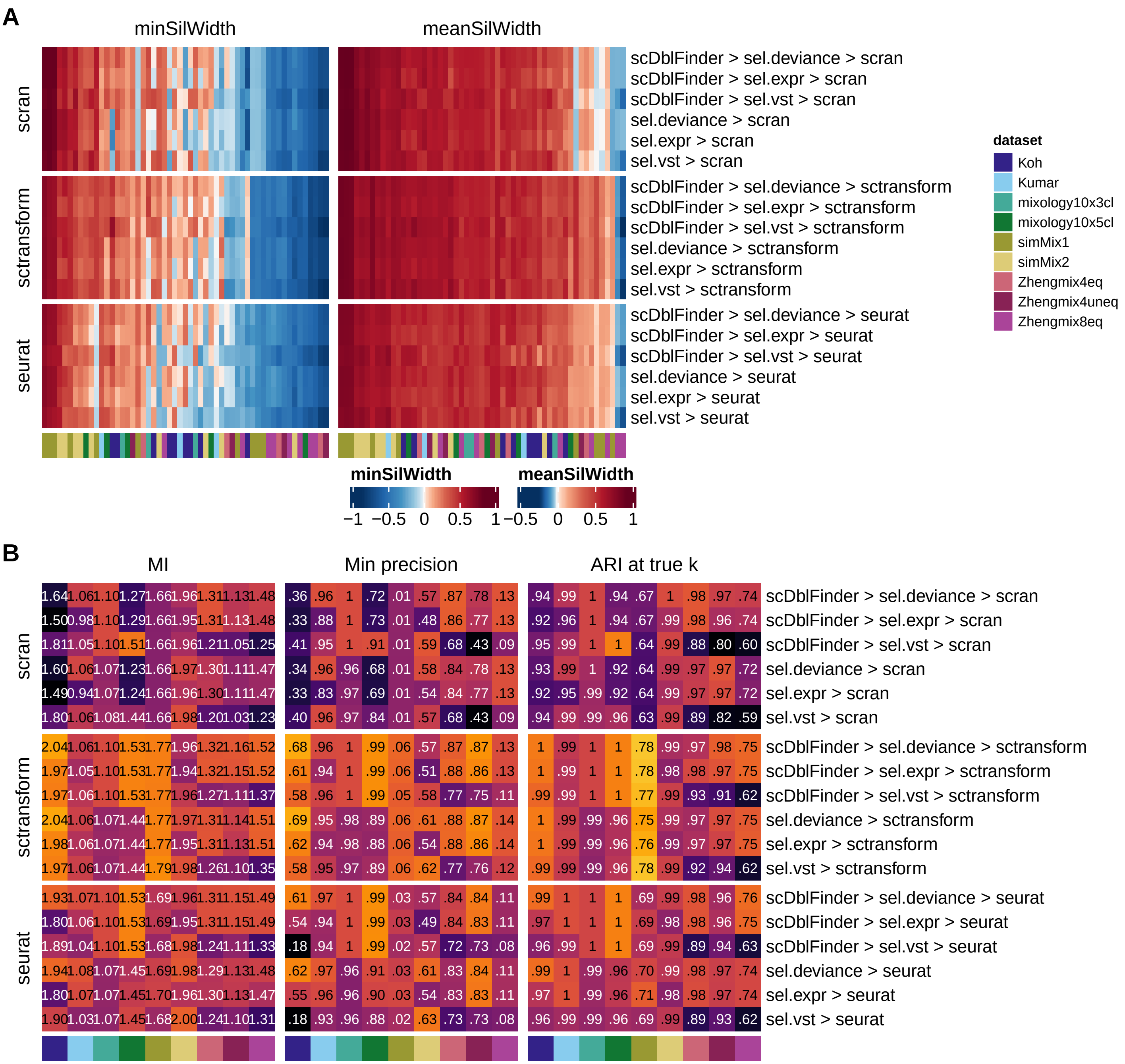

Fig S31

Silhouette widths of the real subpopulations (**A**) and clustering accuracy (**B**) using different combinations of methods.

Fig S32

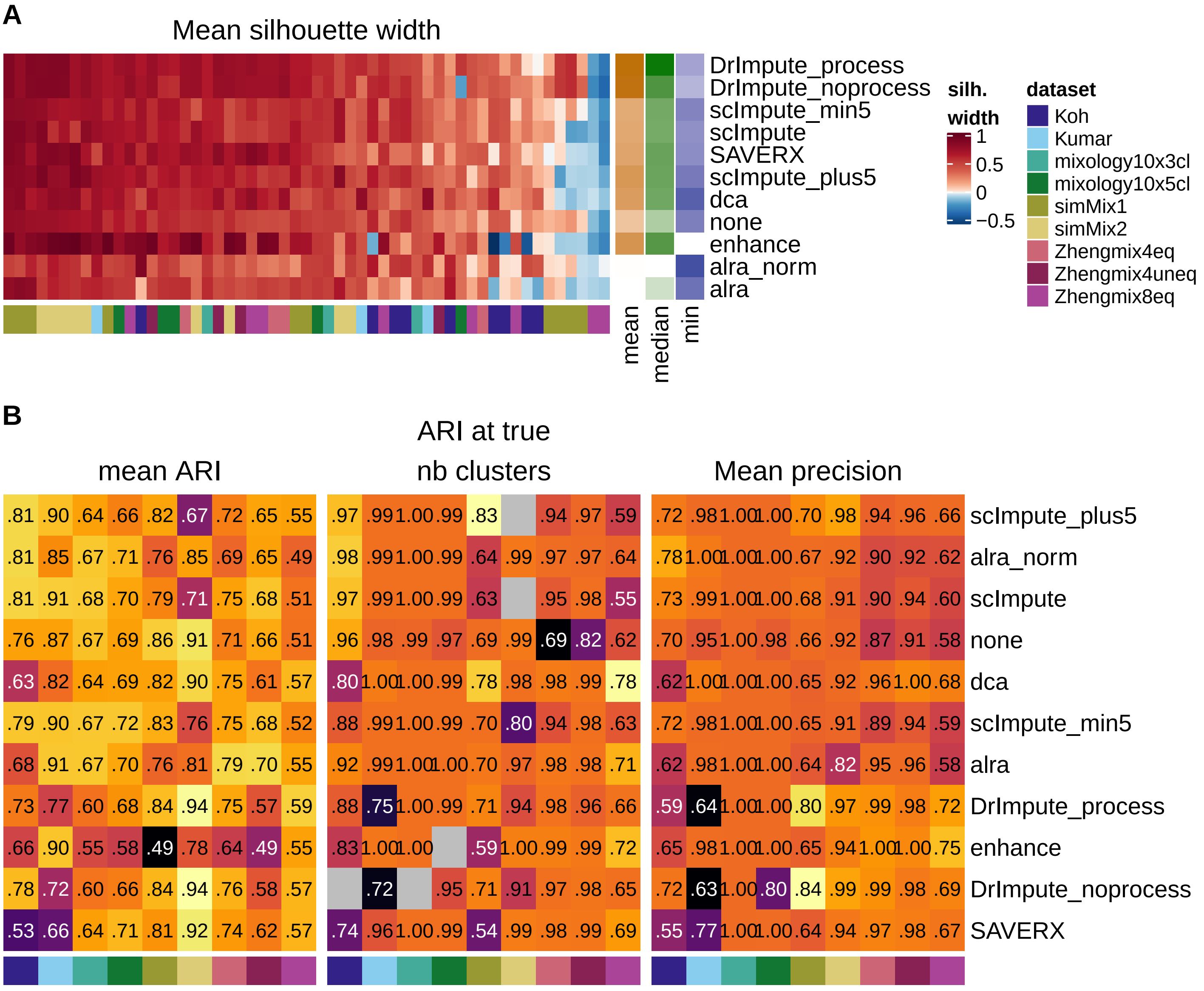

Fig S32

Evaluation of imputation/denoising methods. Average silhouette width (**A**) and clustering accuracy (**B**) with or without (indicated as *none*) application of a denoising/imputation method.

Fig S33

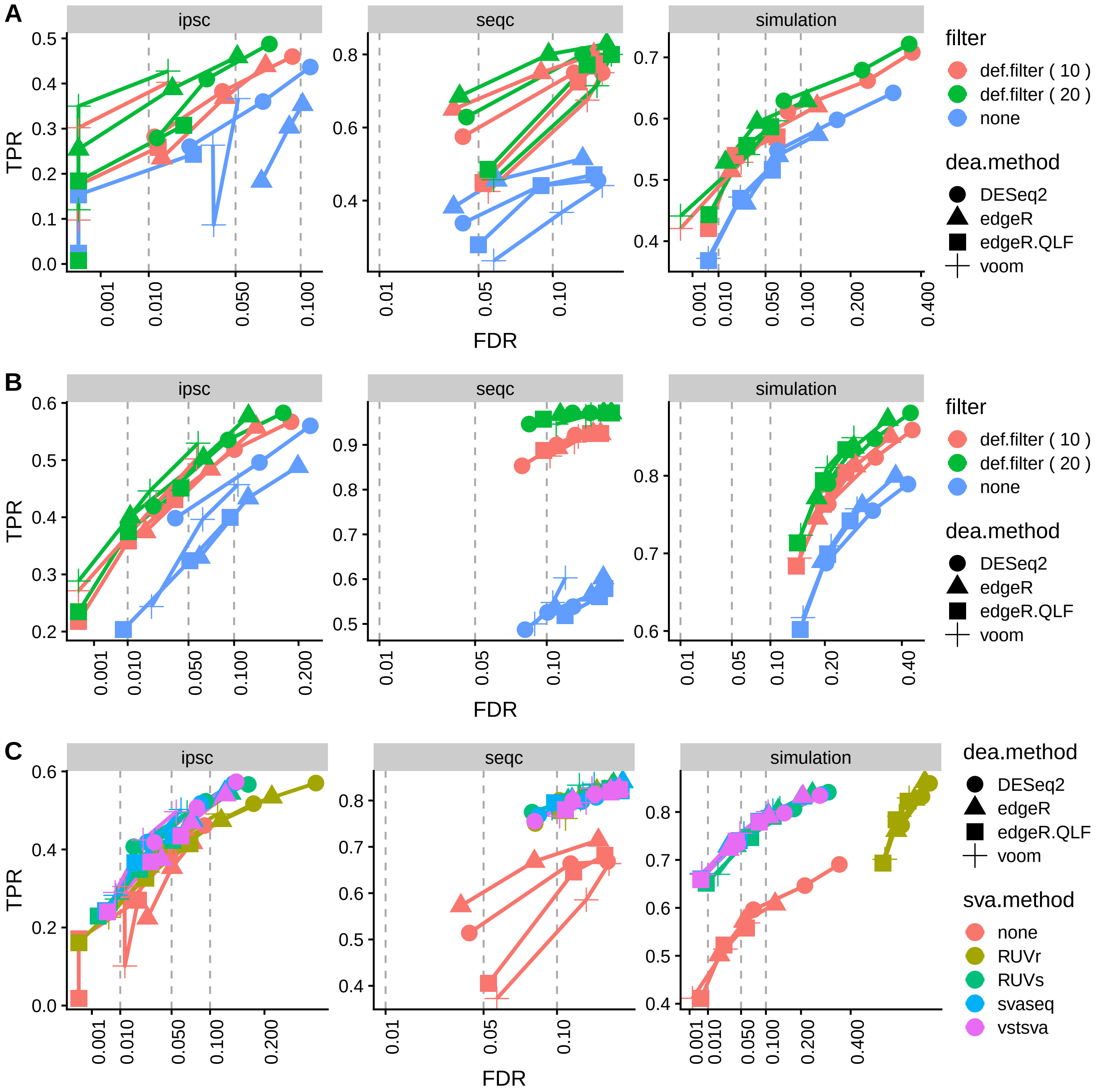

Fig S33

Accuracy of the differential expression analysis across combinations of: **A:** filters and DEA methods (without any SVA-step), **B:** filters and DEA methods (average across the different SVA strategies using either 1 or 2 surrogate variables), **C:** DEA and SVA methods (using one or two surrogate variables).

Fig S34

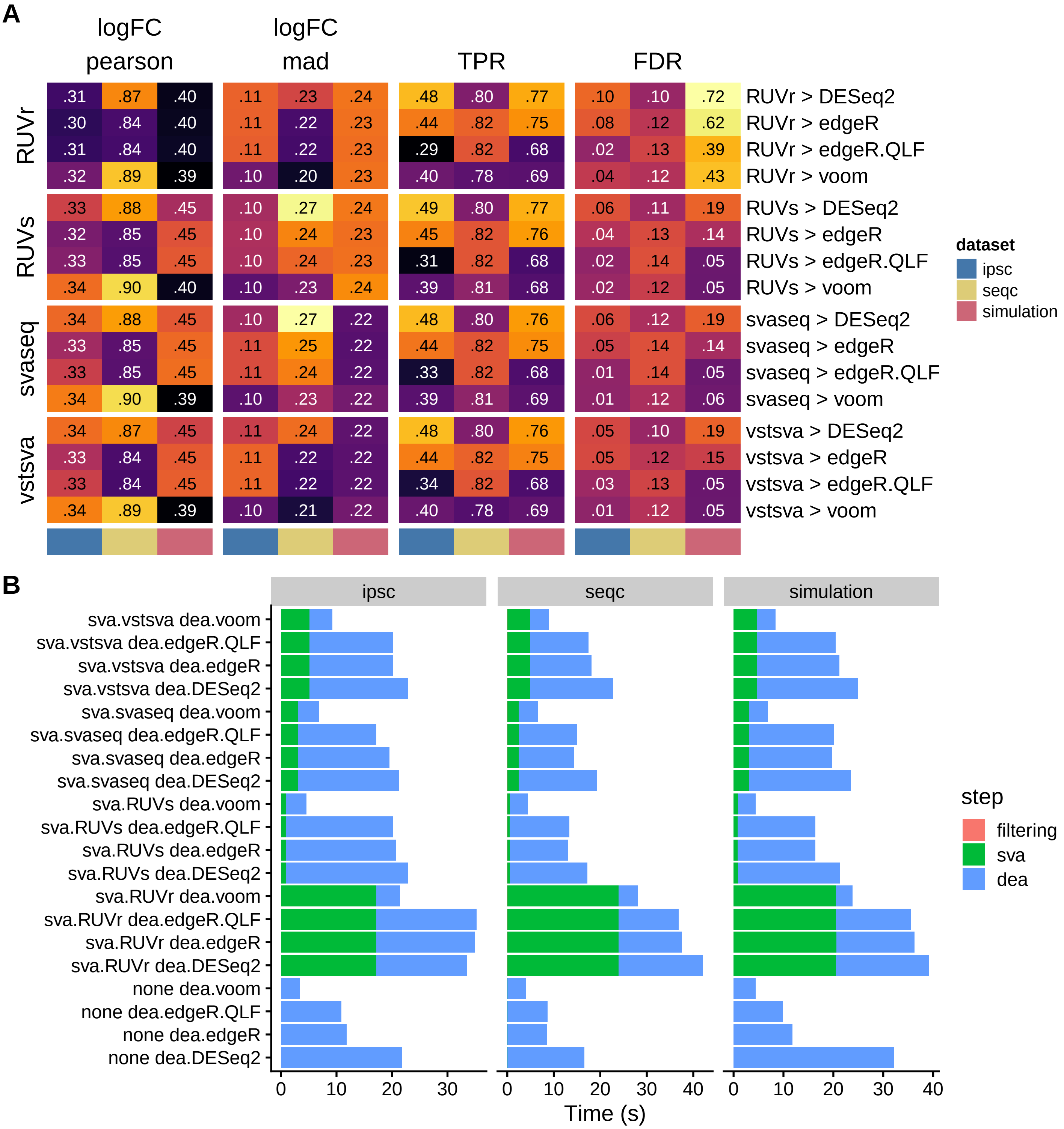

	logFC pearson ipsc	logFC pearson seqc	logFC pearson simulation	logFC mad ipsc	logFC mad seqc	logFC mad simulation	TPR ipsc	TPR seqc	TPR simulation	FDR ipsc	FDR seqc	FDR simulation
RUVr > DESeq2	.31	.87	.40	.11	.23	.24	.48	.80	.77	.10	.10	.72
RUVr > edgeR	.30	.84	.40	.11	.22	.23	.44	.82	.75	.08	.12	.62
RUVr > edgeR.QLF	.31	.84	.40	.11	.22	.23	.29	.82	.68	.02	.13	.39
RUVr > voom	.32	.89	.39	.10	.20	.23	.40	.78	.69	.04	.12	.43
RUVs > DESeq2	.33	.88	.45	.10	.27	.24	.49	.80	.77	.06	.11	.19
RUVs > edgeR	.32	.85	.45	.10	.24	.23	.45	.82	.76	.04	.13	.14
RUVs > edgeR.QLF	.33	.85	.45	.10	.24	.23	.31	.82	.68	.02	.14	.05
RUVs > voom	.34	.90	.40	.10	.23	.24	.39	.81	.68	.02	.12	.05
svaseq > DESeq2	.34	.88	.45	.10	.27	.22	.48	.80	.76	.06	.12	.19
svaseq > edgeR	.33	.85	.45	.11	.25	.22	.44	.82	.75	.05	.14	.14
svaseq > edgeR.QLF	.33	.85	.45	.11	.24	.22	.33	.82	.68	.01	.14	.05
svaseq > voom	.34	.90	.39	.10	.23	.22	.39	.81	.69	.01	.12	.06
vstsva > DESeq2	.34	.87	.45	.11	.24	.22	.48	.80	.76	.05	.10	.19
vstsva > edgeR	.33	.84	.45	.11	.22	.22	.44	.82	.75	.05	.12	.15
vstsva > edgeR.QLF	.33	.84	.45	.11	.22	.22	.34	.82	.68	.03	.13	.05
vstsva > voom	.34	.89	.39	.10	.21	.22	.40	.78	.69	.01	.12	.05

Fig S34

A: Accuracy of the estimated logFC (correlation and median absolute deviation from expected logFCs) and of the differential expression analysis (TPR stands for True Positive Rate, and FDR for False Discovery Rate) across the different combinations of SVA and DEA methods (using max 1 dimension). **B:** Running times of the different methods.

Fig S35

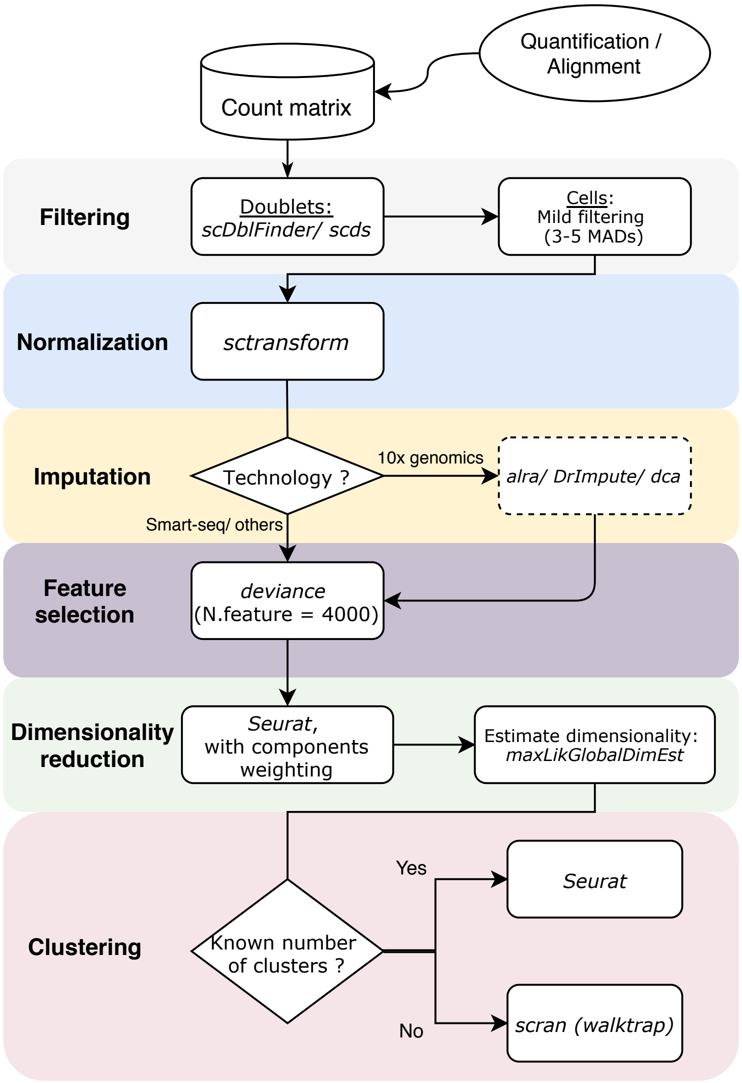

Fig S35: Summary of the recommendations.
