# Supplementary material for: pipeComp, a general framework for the evaluation of computational pipelines, reveals performant single cell RNA-seq preprocessing tools
Source: Genome Biol. 2020 Sep 1;21:227. doi: 10.1186/s13059-020-02136-7 (PMC7465801; doi:10.1186/s13059-020-02136-7)
Supplement: Supplementary file 3 — Additional file 3 Review history. [file 13059_2020_2136_MOESM3_ESM.docx]

Review History

**First round of review**

**Reviewer 1**

**Were you able to assess all statistics in the manuscript, including the appropriateness of statistical tests used?**

Yes: We were able to assess all statistics in the manuscript, including the appropriateness of statistical tests used

**Were you able to directly test the methods?**

Yes

**Comments to author:**

The authors present a framework in R, pipeComp, for organizing and benchmarking computational analyss pipelines. While other benchmarking tools exist for evaluating individual steps, pipeComp considers the impact of combinations of steps on an analysis. The framework is demonstrated through a comparison of tools for pre-processing and performing clustering on scRNA-seq data. While the paper is motivated by an important and challenging question (how compositions of analysis steps impact a full analysis), it is not completely clear how and whether this is actually possible with the pipeComp framework. Major and minor issues regarding (1) the current benchmark analysis and (2) the pipeComp framework are given below.

Major Issues

(1)A. Most results focus on comparisons of individual steps. The paper is motivated by the authors' observation that it is "critical to evaluate not only the single effect of a preprocessing method but also its positive or negative interaction with all parts of a workflow." However, the results presented in the paper largely focus on the marginal impact of each preprocessing step in isolation. Some interactions are considered, e.g. doublet removal and filtering, and normalization and dimension reduction, but these only consider a subset of methods that the authors marginally identify as being "best". How is this different from the benchmarks presented in other papers? Given the combinatorial complexity of evaluating all possible interactions, it is understandable that the authors considered a subset of variations on the pipeline based on a stepwise process, but as a result, it is not clear what benefits are obtained that would not be possible in a marginal benchmark setting. The similar scRNA-seq differential expression comparison of Vieth et al. (2019) already appears to cover many interactions in more detail. More complete analysis of interactions is needed to demonstrate the value of the proposed approach.

(1)B. Conclusions drawn in the text are not clear from the referenced figures, and language is imprecise. For example, when comparing filtering results, the authors recommend "the use of doublet removal followed by relatively mild filtering such as is implemented in our 'default' filtering". What methods are "relatively mild filtering"? Is "pca" or "pca2" "relatively mild"? The results also do not appear to be consistent across datasets. How did the authors reach a single general conclusion? This needs to be clarified.

(1)C. There are generally few attempts to explain inconsistencies in results across methods and datasets. Often, as with filtering and several other workflow steps, the methods showed variable performance across the datasets tested, but there was usually limited investigation as to why this variable performance occurred. What features of the datasets resulted in some methods performing better or worse than on others? Most of the recommendations given were phrased in general terms, which appears to sometimes be an oversimplification based on the presented results.

(2)A. Descriptions and comparisons of the pipeComp framework are lacking. The authors claim that a key contribution of their work is that unlike Vieth et al. (2019), the paper provides a platform for performing pipeline comparisons. How does pipeComp compare to more general workflow tools, e.g. drake? While the pipeline allows for "compiling evaluations on the fly" for benchmarks, how is this formalized in the framework? What is the abstraction that makes pipeComp better than more general tools? Very little detail is provided on the actual framework. For example, are the different methods compared at each step in this workflow, the "parameters" of that step? e.g. would I need to write a single wrapper function for the filtering step that takes the name of the filtering procedure as the input? These details are not explicitly addressed.

(2)B. pipeComp framework appears to be limited in applicability. The software description online only walks through the same use case (clustering to identify sub-populations) described in this paper, rather than other potential goals in scRNA-seq analysis. It would be useful to understand how easily a user could apply this framework to other use cases. Furthermore, many computational pipelines require calling code outside of R, and these functions can be computationally intensive. The pipeComp framework appears to run all combinatorial sets of parameters specified by a user, which can quickly explode. Does this framework allow for parallelization or saving checkpoints? Do all (combinatorial) computations have to occur as one process on one machine?

Minor Issues

- Figure 3A plots are missing labels; it is unclear which datasets are in which plot.

- How much overlap is there between cells removed by 'filtering' and 'doublet removal'? The proportions reported in Figure 4B are hard to interpret without considering this overlap.

- The coloring used in the heatmaps needs to be clarified. The same values appear to have different colors even within the same heatmap (e.g. Figure 5C or Supplementary Figure 10). Are these colored by the rank in rows or columns? More details need to be included in Figure legends.

- Some plots contain too much information, and it is difficult to understand the major takeaways. For example, heatmaps as in Figures 5C, 7B, 8D, etc. are very cluttered, and the ordering of the rows make it hard to easily compare variations on a single method (e.g. Seurat was far away from Seurat.mt_regress and Seurat.feat_regress).

**Reviewer 2**

**Were you able to assess all statistics in the manuscript, including the appropriateness of statistical tests used?**

Yes: Statistics used seemed appropriate

**Were you able to directly test the methods?**

Yes

**Comments to author:**

The paper by Germain et al. introduces the pipeComp R package that facilitates benchmarking of single cell RNA-seq data analysis pipelines. The software is applied to a series of data sets where the true cell labels are known to compare the performance of different analysis tasks including filtering, doublet detection, normalization and clustering to name a few. Metrics that track the performance of methods at these various steps are used to assess how different combinations of tools work together at the pipeline level and general recommendations are made in terms of methods that are most performant.

Comments and questions:

I liked the responsive nature of this study - knowledge from previous benchmarks was applied to give extra focus to this work to examine tasks that had not been previously studied in detail (e.g. doublet detection), explore newer methods or restrict comparisons to classes of methods that had been shown to perform well in previous studies.

Conclusions drawn were clear and well justified by the analysis performed and a lot of effort has been invested to make the analysis reproducible through through an extensive code repository available on GitHub.

My main questions relate to the pipeComp software, which looks like a very useful framework. Can pipeComp be used to benchmark other types of analysis in Bioinformatics or is it heavily specialised for single cell RNA-seq? (from Figure 1A it looks like it is general purpose). Are there plans to release the software as a Bioconductor package? I would recommend this as a way to increase visibility and use of the software in other benchmarking studies and since it uses S4 classes and SingleCellExpression objects, this would seem like a logical step. If however pipeComp is highly specialised and intended only for use in this analysis, then releasing it on GitHub is appropriate.

The pipeComp software is highlighted in the title and abstract, however relatively little attention is paid to it in the main text and I found myself looking up the GitHub page to better understand the flexibility of the pipeline and how it was used. I wonder if an extra Supp Figure could be introduced to make this clearer to the reader e.g. what are the main inputs and functions in a pipeComp analysis and how easy is it to add an extra step such the imputation/denoising analysis featured at the end of the Results section?

Minor points:

Page 2, line 9 Missing 'the' or 'an'? 'all aspects of 'the' scRNAseq workflow.'

Page 5, line 23 'no cell' -> 'no cells' ?

Page 5, line 50, check throughout. Is the method sctransform or SCtransform?

Page 8, line 37 'Elbow' -> `elbow'

Page 8, line 41 `elbow` -> `elbow'

Page 10, line 36, 'Concrete' is perhaps a bit informal / non-scientific (unless you are into building structures)

Page 13, line 24, 'concrete' (see above - perhaps `clear' - I'm sure there are other words)

Table 1, is it possible to add how many cells in total are available per data set (while it is possible to work this out approx from the barplot in Figure 1, it would be easier to list these values in the Table)

Figure 3C, reduce fonts size of data set labels at top of plots as they are cut-off

**Authors Response**

**Point-by-point responses to the reviewers’ comments:**

*We thank the reviewers for their feedback. In addition to the changes requested (described below), we have moved some of the many figures into supplementary in order to improve the overall clarity and put the focus on the main messages. We have also added an entirely different benchmark showcasing the flexibility and wide applicability of the framework.*

*Sections altered were highlighted in red in the manuscript for the reviewers’ convenience.*

*Below are point-by-point responses to the reviewers.*

*******

REVIEWER #1:

MAJOR ISSUES

"(1)A. Most results focus on comparisons of individual steps. The paper is motivated by the authors' observation that it is "critical to evaluate not only the single effect of a preprocessing method but also its positive or negative interaction with all parts of a workflow." However, the results presented in the paper largely focus on the marginal impact of each preprocessing step in isolation. Some interactions are considered, e.g. doublet removal and filtering, and normalization and dimension reduction, but these only consider a subset of methods that the authors marginally identify as being "best". How is this different from the benchmarks presented in other papers? Given the combinatorial complexity of evaluating all possible interactions, it is understandable that the authors considered a subset of variations on the pipeline based on a step-wise process, but as a result, it is not clear what benefits are obtained that would not be possible in a marginal benchmark setting. The similar scRNA-seq differential expression comparison of Vieth et al. (2019) already appears to cover many interactions in more detail. More complete analysis of interactions is needed to demonstrate the value of the proposed approach. "

*ANSWER (1)A: While it is easy, with pipeComp, to evaluate a large number of combinations, the challenge is rather in the presentation of the results: as noted by the reviewer the figures already contain a lot of information, and exploring in detail more combinations would only make it more difficult. The focus on individual steps was driven by considerations on the presentation of the results. This being said, the results reported are already aggregations across common parameter ranges (see methods), and although we do not always discuss these explicitly, these variations are important to establish that observed gains from a given method are robust. Some of the alternatives (e.g. normalization) we discuss throughout; and we have now added a new section and supplementary figures from an overall perspective.*

"(1)B.Conclusions drawn in the text are not clear from the referenced figures, and language is imprecise. For example, when comparing filtering results, the authors recommend "the use of doublet removal followed by relatively mild filtering such as is implemented in our 'default' filtering". What methods are "relatively mild filtering"? Is "pca" or "pca2" "relatively mild"? The results also do not appear to be consistent across datasets. How did the authors reach a single general conclusion? This needs to be clarified. "

*ANSWER (1)B: We clarified that passage, as well as others, and added more specific references of the figures. In that figure (Figure 3), most of the apparent inconsistency between datasets is due to i) the fact that some of the datasets should not, due to their design, have doublets (or even very low-quality cells, in the case of the simulations), ii) some of the differences in F1 scores across the filtering are very mild. Nevertheless, there is irreducible variability across the datasets, which we have tried to explain where possible; where we could not, we had to draw less precise conclusions.*

"(1)C. There are generally few attempts to explain inconsistencies in results across methods and datasets. Often, as with filtering and several other workflow steps, the methods showed variable performance across the datasets tested, but there was usually limited investigation as to why this variable performance occurred. What features of the datasets resulted in some methods performing better or worse than on others? Most of the recommendations given were phrased in general terms, which appears to sometimes be an oversimplification based on the presented results."

*ANSWER (1)C: The text now better addresses the inconsistencies between datasets and suggests explanations for these in the Filtering, Normalization and Dimensionality reduction sections. We also added a new paragraph in the Limitations and open questions section to explain the systematic inconsistencies with two datasets (ZhengMix8eq and simMix1). We also highlighted the differences between methods in the Dimensionality reduction and Normalization and scaling paragraphs.*

"(2)A. Descriptions and comparisons of the pipeComp framework are lacking. The authors claim that a key contribution of their work is that unlike Vieth et al. (2019), the paper provides a platform for performing pipeline comparisons. How does pipeComp compare to more general workflow tools, e.g. drake? While the pipeline allows for "compiling evaluations on the fly" for benchmarks, how is this formalized in the framework? What is the abstraction that makes pipeComp better than more general tools? Very little detail is provided on the actual framework. For example, are the different methods compared at each step in this workflow, the "parameters" of that step? e.g. would I need to write a single wrapper function for the filtering step that takes the name of the filtering procedure as the input? These details are not explicitly addressed."

*ANSWER (2)A: We added a supplementary document describing the framework in more detail and making explicit comparisons with drake and CellBench, and also mentioned key differences in the discussion. We have also added new vignettes to the package. Yes, the methods need to be wrapped as functions which are then provided as parameter values to a step, along with other possible parameters (e.g. arguments of the wrapper function). This is briefly mentioned in the main text, and is now explicitly described in the supplementary document.*

"(2)B. pipeComp framework appears to be limited in applicability. The software description online only walks through the same use case (clustering to identify sub-populations) described in this paper, rather than other potential goals in scRNA-seq analysis. It would be useful to understand how easily a user could apply this framework to other use cases. Furthermore, many computational pipelines require calling code outside of R, and these functions can be computationally intensive. The pipeComp framework appears to run all combinatorial sets of parameters specified by a user, which can quickly explode. Does this framework allow for parallelization or saving checkpoints? Do all (combinatorial) computations have to occur as one process on one machine?"

*ANSWER (2)B:*

*The pipeComp framework is easily applicable to very different use cases. To illustrate its applicability and flexibility, we added a small, entirely different benchmark of the interaction between filtering, removal of unwanted variation, and differential expression analysis methods in bulk RNAseq data. The whole process of creating the PipelineDefinition, the evaluation metrics, some wrappers, and running the benchmark is now described step-by-step in a new vignette of the package (“pipeComp_dea”). This, as well as the creation of the benchmark datasets, was performed in roughly one day of work, which testifies to the utility of the package. Incidentally, this analysis also provides a much needed evaluation of the robustness of SVA/RUV methods, which is presented at the end of the Results section.*

*We have now added a new supplementary document describing the framework in more details.*

*We agree that many computational pipelines require calling code outside of R. This is entirely possible in our framework by writing R wrappers for the non-R methods (as we did for python-based imputation methods), however we agree that in certain circumstances other frameworks might be easier to work with.*

*By default, pipeComp runs all combinatorial sets of parameters, but as was mentioned in the paper and vignettes, users can also run only subsets of the combinations. We have now tried to clarify this.*

*The different combinations can be parallelized and spread across as many threads/machines as desired. This is implemented via BiocParallel, which enables the use of various parallelization backends. Additional combinations can also be appended to previous results. In addition to being described in the package’s documentation, we have now explained this along with more detailed discussion of the framework in the new supplementary document, which also includes direct comparisons to drake and CellBench (the key differences being highlighted in the main text’s discussion).*

*The package does not currently support saving checkpoints, as drake for instance does. We chose not to use on-disk caching because, as we now explain, it would have required prohibitive amounts of storage for benchmarks such as the ones we produced. However, results from different runs can easily be merged.*

MINOR ISSUES

- "Figure 3A plots are missing labels; it is unclear which datasets are in which plot."

*We added the missing labels.*

- "How much overlap is there between cells removed by 'filtering' and 'doublet removal'? The proportions reported in Figure 4B are hard to interpret without considering this overlap."

*We added a supplementary figure to this effect (there is very little overlap).*

- "The coloring used in the heatmaps needs to be clarified. The same values appear to have different colors even within the same heatmap (e.g. Figure 5C or Supplementary Figure 10). Are these colored by the rank in rows or columns? More details need to be included in Figure legends."

*We now changed the coloring scheme for most metric heatmaps to improve clarify, and described it in the legend. While the numbers printed in the heatmap are the raw metric values, mapping colors directly to them typically leads to the colors highlighting chiefly baseline dataset differences, which is less informative to show within-dataset differences. On the other hand, scaling by column (i.e. dataset), as we previously did, leads to the same differences in color having very different meanings across datasets, and thus nearly meaningless when there is very little variation in a given dataset. We therefore chose to color by number of matrix-wise median absolute deviations from the column-/dataset-wise median. This has the virtue that the same difference in colors indicates changes of the same magnitude, while not being screened off by baseline differences between datasets.*

- "Some plots contain too much information, and it is difficult to understand the major takeaways. For example, heatmaps as in Figures 5C, 7B, 8D, etc. are very cluttered, and the ordering of the rows make it hard to easily compare variations on a single method (e.g. Seurat was far away from Seurat.mt_regress and Seurat.feat_regress)."

*We now used row-splitting to improve the clarity of the figures.*

*******

REVIEWER #2

COMMENTS AND QUESTIONS:

"1. My main questions relate to the pipeComp software, which looks like a very useful framework. Can pipeComp be used to benchmark other types of analysis in Bioinformatics or is it heavily specialised for single cell RNA-seq? (from Figure 1A it looks like it is general purpose). Are there plans to release the software as a Bioconductor package? I would recommend this as a way to increase visibility and use of the software in other benchmarking studies and since it uses S4 classes and SingleCellExpression objects, this would seem like a logical step. If however pipeComp is highly specialised and intended only for use in this analysis, then releasing it on GitHub is appropriate."

*ANSWER 1: pipeComp was developed as a general framework, and the specialized tools for the benchmark of scRNAseq clustering are encapsulated in a specific PipelineDefinition (available through the `scrna_pipeline()` function of the package). To illustrate the applicability and flexibility of the framework, we created another PipelineDefinition tackling a very different problem, namely the interaction between filtering, removal of unwanted variation, and differential expression analysis methods in bulk RNAseq data. A new vignette in the package now illustrates the step by step creation of this pipeline and benchmark (which we set up and performed in roughly one day, including dataset preparation), and the results are included in the paper (incidentally providing a useful evaluation of SVA/RUV methods). We’ve submitted pipeComp to Bioconductor, and added a mention to this effect in the paper.*

"2. The pipeComp software is highlighted in the title and abstract, however relatively little attention is paid to it in the main text and I found myself looking up the GitHub page to better understand the flexibility of the pipeline and how it was used. I wonder if an extra Supp Figure could be introduced to make this clearer to the reader e.g. what are the main inputs and functions in a pipeComp analysis and how easy is it to add an extra step such the imputation/denoising analysis featured at the end of the Results section?"

*ANSWER 2: We have now added a supplementary document including a new figure explaining the inner working of the package and describing the main inputs and functions. It is very simple to add or remove steps to a PipelineDefinition, as documented in the vignettes, however we have chosen not to enter into these details in the paper itself.*

MINOR POINTS:

"Page 2, line 9 Missing 'the' or 'an'? 'all aspects of 'the' scRNAseq workflow.'

Page 5, line 23 'no cell' -> 'no cells' ?

Page 5, line 50, check throughout. Is the method sctransform or SCtransform?

Page 8, line 37 'Elbow' -> `elbow'

Page 8, line 41 `elbow` -> `elbow'

Page 10, line 36, 'Concrete' is perhaps a bit informal / non-scientific (unless you are into building structures)

Page 13, line 24, 'concrete' (see above - perhaps `clear' - I'm sure there are other words)

Table 1, is it possible to add how many cells in total are available per data set (while it is possible to work this out approx from the barplot in Figure 1, it would be easier to list these values in the Table)

Figure 3C, reduce fonts size of data set labels at top of plots as they are cut-off"

*ANSWER: We thank the reviewer, all these have now been changed, and the number of cells/features is now mentioned in the table.*

**Second round of review**

**Reviewer 1**

While the authors have addressed some of my previous concerns with additional text and clarification of results, the paper still does not fully address my primary concern. It is critical that the paper demonstrate not only that the pipeComp framework can be used to benchmark pipelines, but that the results of this sort of complex benchmark can actually be interpreted (despite being difficult).

(1)A. The new section, “Overview of Interactions Between Steps,” is a great improvement towards demonstrating pipeComp’s ability to reveal insights about interactions among multiple steps in a pipeline. The results presented here appear both interesting and important, e.g. the impact of dimensionality vs. clustering method. However, this section still feels like an afterthought, with all figures left to the supplement. Drawing concrete conclusions about interactions may be challenging with so many combinations of parameters under consideration. However, because one of the main contributions of pipeComp and primary differentiators from alternatives is this ability to evaluate interactions, it is necessary to demonstrate that this adds substantial, not merely peripheral, value to the resulting analysis. The discussion of the interactions should be more substantial and placed more prominently in the paper, possibly with a figure in the main text.

(1)B. The added language and expanded sections clarify these concerns.

(1)C. The additional commentary on some of the differentiators among the datasets help clarify the inconsistent results across datasets. These results are particularly interesting and tie closely with the concern raised above. In addition to evaluating across many pipeline parameters, the authors are also considering many datasets. It is understandable that inconsistent results across datasets can make precise conclusions across many pipeline parameters difficult. However, rather than trying to “average” across datasets to find a single “best” set of pipeline parameters, it is more practically useful to highlight these inconsistencies for readers. More explicit connections should be drawn between characteristics of the datasets (e.g. Table 1 features) and the effects of steps throughout the pipeline. Although there has been some good discussion of this added in the paper, it is not always clear how these characteristics should affect the analysis decisions. For example, since some datasets were noted to be especially sparse or to originate from different sequencing technologies, it would be useful to more clearly describe how those characteristics affect the recommendations being made. This additional clarity will be important for practitioners working with datasets that share any of the qualities that led to inconsistencies, especially systematic inconsistencies, in the results.

In a similar vein, it would also be useful to clarify unusual inconsistencies surrounding the methods tested. In particular, it is unexpected that scVI performed poorly in most cases, since previous work has found strong results with this method, and the original paper introducing scVI demonstrated successful analysis on datasets derived from the Zheng data. No details appear to be available on the parameters used when running scVI. This should also be described in the paper.

2(A). The additional supplementary document and comparisons with other benchmark alternatives sufficiently address the original concern.

2(B). The additional use case and corresponding vignette help demonstrate the flexibility of pipeComp. As mentioned in the previous point, the supplementary document also helps clarify and explain the developed framework.

**Authors Response**

**Point-by-point responses to the reviewers’ comments:**

*We thank the reviewer for the further feedback. We made the changes requested (described below), as well as other minor additions (e.g. new testing of molecular cross-validation to estimate dimensionality).*

*Sections altered were highlighted in red in the manuscript for the reviewers’ convenience.*

*Below are answers each of the reviewer’s remaining comments:*

REVIEWER #1:

"While the authors have addressed some of my previous concerns with additional text and clarification of results, the paper still does not fully address my primary concern. It is critical that the paper demonstrate not only that the pipeComp framework can be used to benchmark pipelines, but that the results of this sort of complex benchmark can actually be interpreted (despite being difficult).

(1)A. The new section, "Overview of Interactions Between Steps," is a great improvement towards demonstrating pipeComp's ability to reveal insights about interactions among multiple steps in a pipeline. The results presented here appear both interesting and important, e.g. the impact of dimensionality vs. clustering method. However, this section still feels like an afterthought, with all figures left to the supplement. Drawing concrete conclusions about interactions may be challenging with so many combinations of parameters under consideration. However, because one of the main contributions of pipeComp and primary differentiators from alternatives is this ability to evaluate interactions, it is necessary to demonstrate that this adds substantial, not merely peripheral, value to the resulting analysis. The discussion of the interactions should be more substantial and placed more prominently in the paper, possibly with a figure in the main text."

*ANSWER:*

*We added a plotting function in the package to facilitate the visualization of evaluation metrics across large numbers of combinations, and used it to generate a new main figure. From this, we highlighted the main factors driving the metrics, linking to our previous ANOVA, as well as top combinations. We now also highlighted the statistically significant interactions (all negative).*

REVIEWER #1:

"(1)C. The additional commentary on some of the differentiators among the datasets help clarify the inconsistent results across datasets. These results are particularly interesting and tie closely with the concern raised above. In addition to evaluating across many pipeline parameters, the authors are also considering many datasets. It is understandable that inconsistent results across datasets can make precise conclusions across many pipeline parameters difficult. However, rather than trying to "average" across datasets to find a single "best" set of pipeline parameters, it is more practically useful to highlight these inconsistencies for readers. More explicit connections should be drawn between characteristics of the datasets (e.g. Table 1 features) and the effects of steps throughout the pipeline. Although there has been some good discussion of this added in the paper, it is not always clear how these characteristics should affect the analysis decisions. For example,

since some datasets were noted to be especially sparse or to originate from different sequencing technologies, it would be useful to more clearly describe how those characteristics affect the recommendations being made. This additional clarity will be important for practitioners working with datasets that share any of the qualities that led to inconsistencies, especially systematic inconsistencies, in the results."

*ANSWER:*

*We understand the reviewer’s point and, where possible, we tried to describe relationships between characteristics of the datasets and differences in performances of certain methods. However, we must emphasize that, first, although some datasets were generally more ‘difficult’ than others, most of our observations about top methods did hold across the vast majority of datasets. Indeed, the relative changes to some measures, in particular ARI, might vary across datasets; however as we emphasized in the paper, this is only due to the impact of resolution on the number of clusters called, which is also shown when considering the ARI score alongside MI and ‘ARI at true’ k. We believe that the key messages given by the three metrics together, and highlighted in the paper, are fairly robust across datasets.*

*Moreover, when some datasets did react differently, it is often a single one (e.g. simMix2 in feature selection), making it difficult to attribute the inconsistency to a specific characteristic of the dataset. In such contexts, we refrained from making undue inferences.*

*There were of course notable exceptions, which we tried to discuss in the text. For instance, we explained several discrepancies in the doublet/filtering steps, higher gains (though not always robust) from imputation for shallow datasets, and (as expected) MCVR failing on non-UMI datasets.*

REVIEWER #1:

"In a similar vein, it would also be useful to clarify unusual inconsistencies surrounding the methods tested. In particular, it is unexpected that scVI performed poorly in most cases, since previous work has found strong results with this method, and the original paper introducing scVI demonstrated successful analysis on datasets derived from the Zheng data. No details appear to be available on the parameters used when running scVI. This should also be described in the paper."

*ANSWER:*

*It is true that the difference of performance between our study and the original paper is quite strong. After a discussion with the authors of scVI, we were able to slightly improve the implementation of scVI and its performance (c.f. Supplementary Figure 15 , Normalization and scaling and Dimensionality reduction sections), in particular by reducing the number of features prior to normalization. We integrated a list of limitations of scVI with regard to our study’s design in the Limitations and open questions section. Other studies with the same limitations (i.e. datasets with few cells; Hou et al. 2020, bioRxiv, Aparicio et al. 2020, Patterns) found similar results as ours and we integrated this point in the text (same section and paragraph). We thank the reviewer for highlighting this inconsistency as it lead us to improve scVI implementation and pinpoint its limitations in our study.*

*Concerning the Zheng data, it is true that both the scVI article and our study use the same reference. However two major points differ between our studies and likely have an impact on scVI’s performance with regard to this data: 1) as specified, we used the different samplings of the Zheng data generated by Duo et al. (2018), which are smaller than the 12k cells Zheng dataset; 2) we considered the FACS as a ground truth for cell identity, whereas the study of Lopez et al. was based on the clustering returned by Seurat. It is very likely that these two differences, especially the limitation of scVI to low amount of cells, explain the inconsistency between the two studies.*

*The exact parameters used for scVI are now specified in the scVI normalization and LD section of the Methods. The code for running scVI along all other wrappers used in the study are also still available in the github repositories of the package (see link in the first paragraph of the Code and data availability section of the Methods).*

**Third round of Review**

**Reviewer 1**

I appreciate the extra analyses and level of detail provided by the authors. While this alleviates some of the issues I had flagged, the changes do not fully address all of them.

Regarding (1)A:

A plotting function to visualize evaluation metrics across parameter combinations is a nice contribution. While this will be helpful as a form of exploratory analysis for users, it isn’t a clear summary of the results. The newly included Figures 8 and 9 are busy and difficult to interpret. One of the main results of Figure 8 ("which steps are most important") is shown much more concisely in Supplementary Figure 29. This is a great example of a much clearer summary of the results. Supplementary Figure 29 should be cleaned up (stacking “% variance explained” across methods is odd) and included as a main figure with or in place of the first heatmap in Figure 8.

The concern I raised before is that there is not enough attention paid to concrete examples of interactions, even though analyzing interactions is one of the primary contributions of the method. Figures 8 and 9 in their present form do not show concrete examples; there is a lot of information contained within the figures, and much of it does not appear to follow a convincing trend that can be generalized into a recommendation. If most of the interactions are challenging to generalize, then it would be more useful to present a figure (or figures) demonstrating a few specific and useful examples of interactions that do follow clear trends. For example, the authors mention “some significant interactions” based on an ANOVA analysis at the end of page 11, and this is interesting. This ANOVA analysis of interactions is neither described nor shown in more detail anywhere. The paper only describes an ANOVA analysis without any interactions. This should be added.

Regarding (1)C:

First, the additional clarification and analysis surrounding scVI is very helpful, and my concern regarding the benchmarking of the method has been resolved.

Second, it is a fair point that inconsistencies in a small number of datasets cannot lead to generalizations about characteristics of those datasets. That said, many of the recommendations do not appear to hold true across the vast majority of datasets, and instead the results are quite heterogeneous in the figures. The authors’ recommendations do not always follow clearly from the figures. For example, Figure 3A is a very positive example of a clear figure with an actionable and well-supported recommendation. Figures 3B, 4B, 4C, 6C, 6D, 7A, 8, and 9 are much more challenging to interpret and do not clearly demonstrate (or always agree with) the accompanying recommendation. For instance,

• Figure 3B: the authors state that scDblFinder improved accuracy of clustering across all benchmark datasets, but that isn’t true, e.g. simMix2, Zhengmix4eq.

• Figure 7A: the authors state that better values of MI and min_pr are typically obtained using walktrap, but it is not clear from the heatmap that walktrap broadly works well, nor is it easy to interpret how other choices (e.g. Annoy vs. exact or KNN vs. rank SNN vs. SNN) affect this recommendation.

These are just examples that I could quickly identify (the complex figures do not make it easy). I had previously suggested the authors providing more commentary on specific characteristics of the datasets or otherwise demonstrating a clearer result in an effort to explain the heterogeneity within the results. If this is not possible, then the authors should instead consider paring down the main figures and accompanying recommendations to just those that can be clearly and specifically demonstrated.

**Authors Response**

**Point-by-point responses to the reviewers’ comments:**

Reviewer #1:

Regarding (1)A: A plotting function to visualize evaluation metrics across parameter combinations is a nice contribution. While this will be helpful as a form of exploratory analysis for users, it isn't a clear summary of the results. The newly included Figures 8 and 9 are busy and difficult to interpret. One of the main results of Figure 8 ("which steps are most important") is shown much more concisely in Supplementary Figure 29. This is a great example of a much clearer summary of the results. Supplementary Figure 29 should be cleaned up (stacking "% variance explained" across methods is odd) and included as a main figure with or in place of the first heatmap in Figure 8.

*ANSWER:*

*We modified the presentation of the ANOVA results (formerly Suppl. Fig S29, now Figure 8) as suggested by the reviewer (simplified and removed the stacking), moved it to be a main figure, and instead moved the more complicated Figure 8 to the Supplement (now Suppl. Fig S29).*

Reviewer #1:

The concern I raised before is that there is not enough attention paid to concrete examples of interactions, even though analyzing interactions is one of the primary contributions of the method. Figures 8 and 9 in their present form do not show concrete examples; there is a lot of information contained within the figures, and much of it does not appear to follow a convincing trend that can be generalized into a recommendation. If most of the interactions are challenging to generalize, then it would be more useful to present a figure (or figures) demonstrating a few specific and useful examples of interactions that do follow clear trends. For example, the authors mention "some significant interactions" based on an ANOVA analysis at the end of page 11, and this is interesting. This ANOVA analysis of interactions is neither described nor shown in more detail anywhere. The paper only describes an ANOVA analysis without any interactions. This should be added.

*ANSWER:*

*The only robust, concrete examples of interactions are already discussed in the manuscript, subsection ‘Overview of interactions between steps’. We now show the results of the ANOVA analysis of interactions in Suppl. Fig S30. However, all statistically significant interactions are those already discussed in the manuscript, namely relative to scran normalization when underestimating dimensionality with the elbow method (3rd paragraph of the ‘Overview of interactions between steps’ subsection).*

*Due to the complex nature of the former Figure 8, we moved it to Supplement (Suppl. Fig S29). However, we disagree about Figure 9, which gives very clear, generalizable results (one SVA method showing bad results, and the others systematically improving performance and being robust to increases in the number of SVs). In an attempt to make this message yet clearer, we simplified the first panel of Figure 9, moving some secondary results to the associated supplementary figure (Suppl. Fig S33).*

Reviewer #1:

Regarding (1)C: First, the additional clarification and analysis surrounding scVI is very helpful, and my concern regarding the benchmarking of the method has been resolved.

Second, it is a fair point that inconsistencies in a small number of datasets cannot lead to generalizations about characteristics of those datasets. That said, many of the recommendations do not appear to hold true across the vast majority of datasets, and instead the results are quite heterogeneous in the figures. The authors' recommendations do not always follow clearly from the figures. For example, Figure 3A is a very positive example of a clear figure with an actionable and well-supported recommendation. Figures 3B, 4B, 4C, 6C, 6D, 7A, 8, and 9 are much more challenging to interpret and do not clearly demonstrate (or always agree with) the accompanying recommendation. For instance,

* Figure 3B: the authors state that scDblFinder improved accuracy of clustering across all benchmark datasets, but that isn't true, e.g. simMix2, Zhengmix4eq.

* Figure 7A: the authors state that better values of MI and min_pr are typically obtained using walktrap, but it is not clear from the heatmap that walktrap broadly works well, nor is it easy to interpret how other choices (e.g. Annoy vs. exact or KNN vs. rank SNN vs. SNN) affect this recommendation.

*ANSWER:*

*. Regarding Figure 3B; it is true that our statement did not perfectly fit the results of the figure. We nuanced our conclusion concerning scDblFinder in the last paragraph of the ‘Doublet detection’ subsection. Rather, the examples mentioned by the reviewer do not, due to their design, have any doublets (and the difference for simMix2 is extremely low in magnitude). We added further precision concerning this point in the text (same subsection) and in the legend of Figure 8 and tried to clarify the description of those results.*

*Regarding Figure 7A, we would like to highlight it shows that MI is systematically lower for both fast_greedy and Seurat clustering, among both normalizations and among all datasets. However, it is true that the conclusion is less clear with regard to min_pr (we had meant it to apply to the Annoy KNN - walktrap combination). We have now clarified this passage (3rd paragraph of the ‘Clustering’ subsection). However, we had also refrained from making any strong recommendation from these results, simply pointing out the major differences in computing time and control/arbitrariness of the number of clusters.*
